# Highly flexible infection programs in a specialized wheat pathogen

**DOI:** 10.1002/ece3.4724

**Published:** 2018-12-26

**Authors:** Janine Haueisen, Mareike Möller, Christoph J. Eschenbrenner, Jonathan Grandaubert, Heike Seybold, Holger Adamiak, Eva H. Stukenbrock

**Affiliations:** ^1^ Environmental Genomics Group Max Planck Institute for Evolutionary Biology Plön Germany; ^2^ Environmental Genomics Group Christian‐Albrechts University Kiel Kiel Germany; ^3^ Fungal Biology and Pathogenicity Institute Pasteur Paris France

**Keywords:** confocal microscopy, expression phenotypes, host specialization, infection phenotypes, plant–fungus interactions, *Zymoseptoria tritici*

## Abstract

Many filamentous plant pathogens exhibit high levels of genomic variability, yet the impact of this variation on host–pathogen interactions is largely unknown. We have addressed host specialization in the wheat pathogen *Zymoseptoria tritici*. Our study builds on comparative analyses of infection and gene expression phenotypes of three isolates and reveals the extent to which genomic variation translates into phenotypic variation. The isolates exhibit genetic and genomic variation but are similarly virulent. By combining confocal microscopy, disease monitoring, staining of ROS, and comparative transcriptome analyses, we conducted a detailed comparison of the infection processes of these isolates in a susceptible wheat cultivar. We characterized four core infection stages: establishment, biotrophic growth, lifestyle transition, and necrotrophic growth and asexual reproduction that are shared by the three isolates. However, we demonstrate differentiated temporal and spatial infection development and significant differences in the expression profiles of the three isolates during the infection stages. More than 20% of the genes were differentially expressed and these genes were located significantly closer to transposable elements, suggesting an impact of epigenetic regulation. Further, differentially expressed genes were enriched in effector candidates suggesting that isolate‐specific strategies for manipulating host defenses are present in *Z. tritici*. We demonstrate that individuals of a host‐specialized pathogen have highly differentiated infection programs characterized by flexible infection development and functional redundancy. This illustrates how high genetic diversity in pathogen populations results in highly differentiated infection phenotypes, which fact needs to be acknowledged to understand host–pathogen interactions and pathogen evolution.

## 1 INTRODUCTION

Plants are colonized by a broad diversity of microbial species. Many plant‐associated microbes are commensals that utilize the plant as a scaffold for their proliferation by feeding on readily available nutrients. Other microbial species engage in more intimate interactions with plants, either as mutualistic symbionts where both partners benefit from the interaction or as antagonistic symbionts where the microbial species exploits the plant for growth and reproduction (Zeilinger et al., 2015). Mutualistic and antagonistic interactions between plants and co‐existing microbial species rely on molecular interactions between plant receptors and signaling pathways and microbial molecules including microbe‐associated molecular patterns (MAMPs) and effectors. The successful establishment of such intimate plant–microbe interactions, in one or the other form, depends on the ability of the microbial partner to suppress and interfere with the immune responses of the plant (Dodds & Rathjen, 2010; Jones & Dangl, 2006; Lo Presti et al., 2015) and to colonize and reproduce associated to plant tissues (Haueisen & Stukenbrock, 2016; van der Does & Rep, 2017). These symbioses are therefore considered to be highly specialized and the result of co‐evolution.

In a small number of model systems, key molecules involved in the antagonistic interaction of plants with their specialized pathogens have been characterized, for example, the *Ustilago maydis* effector Tin2 that interferes with the anthocyanin biosynthesis pathway in its host maize (Tanaka et al., 2014) or the tyrosine phosphatase HopAO1 of *Pseudomonas syringae* which inhibits activation of a plant pattern recognition receptor (Macho et al., 2014). While the relevance of these molecular interactions for the studied host–pathogen genotype combinations is undisputed, the consequences at the population level have so far been poorly addressed.

While many plant pathogens are known to be highly specialized to their host, substantial genetic variation is found in many species. Genetic variation can translate into phenotypic variation that can be important for pathogen populations to persist in changing environments (Fisher, Hawkins, Sanglard, & Gurr, 2018; Möller & Stukenbrock, 2017). However, host specialization may require a highly specialized and definite infection program. An intriguing question is which consequences genetic and phenotypic variation entails for the diversity of host interactions in pathogens that are specialized and host‐specific?

In this study, we addressed the extent of phenotypic variation in a fungal plant pathogen characterized by a high level of genomic variability. Our aim was to study phenotypic diversity beyond virulence phenotypes that are limited to the description of quantitative virulence which is based on the quantification of disease symptoms at a particular time of infection. We therefore considered morphological and temporal infection development as well as stage‐specific gene expression to characterize the infection phenotypes of pathogen individuals. We used the wheat pathogen *Zymoseptoria tritici* (syn. *Mycosphaerella graminicola*) as a model to investigate how the development of disease symptoms and the transcriptional program induced during infection vary among three field isolates from geographically distinct locations. *Zymoseptoria tritici* has served as a prominent model in population genetic studies of crop pathogens, and genetic variation has been assessed from local scales in individual lesions on single leaves up to continental scales. The amount of genetic variation in a *Z. tritici* field population is comparable to the variation found on a continental scale suggesting very high levels of genetic variation in local populations and little subdivision between populations (Linde, Zhan, McDonald, & a., 2002; McDonald et al., 2016; Zhan, Pettway, & McDonald, 2003). *Zymoseptoria tritici *has been described as hemibiotroph (Ponomarenko, Goodwin, & Kema, 2011) or latent necrotroph (Sánchez‐Vallet, McDonald, Solomon, & McDonald, 2015) with a prolonged epiphytic stage (Fones, Eyles, Kay, Cowper, & Gurr, 2017). Infections are biphasic and characterized by a long asymptomatic, biotrophic phase, followed by necrotrophic growth where the fungus degrades and takes up nutrients from dead host cells (Ponomarenko et al., 2011; Rudd et al., 2015). In spite of the general importance of this pathogen in wheat fields, the life cycle and infection biology of *Z. tritici* are so far poorly understood.

The haploid genome of *Z. tritici* comprises a high number of accessory chromosomes ranging from 400 kb to 1 Mb in size in the reference isolate IPO323 (Goodwin et al., 2011; Wittenberg et al., 2009). Recent studies provide evidence for the presence of virulence determinants on the accessory chromosomes; however, the genes responsible for these effects have so far not been identified (Habig, Quade, & Stukenbrock, 2017). Furthermore, several genome‐wide association and quantitative trait loci mapping studies have linked a variety of phenotypic traits to genetic variants and candidate genes (Hartmann, Sánchez‐Vallet, McDonald, & Croll, 2017; Lendenmann, Croll, & McDonald, 2015; Lendenmann, Croll, Stewart, & McDonald, 2014; Mirzadi Gohari et al., 2015; Stewart et al., 2017; Zhong et al., 2017).

Here, we investigated how infection of a susceptible host by genetically and morphologically distinct isolates results in similar quantitative virulence. By combining confocal microscopy, disease monitoring, reactive oxygen species (ROS) localization, and transcriptome analyses, we compiled a detailed characterization of infection phenotypes of three *Z. tritici* isolates. We hypothesized that high genetic diversity not only increases the evolutionary potential of the pathogen but also results in a variety of host–pathogen interactions that cause a range of different infection phenotypes. Our combined comparative analyses enabled us to characterize infection morphology and gene expression of the three *Z. tritici* isolates, including a core infection program and isolate‐specific infection phenotypes. We conclude that host specialization of *Z. tritici* entails a substantial amount of variation in terms of the temporal, spatial, and molecular host–pathogen interactions. We speculate that this phenotypic variation is important for the pathogen to rapidly respond to changing environments, and we underline the need of considering variation at this level in the study of pathogen evolution and the development of disease control strategies.

## 2 MATERIALS AND METHODS

### 2.1 Isolates and growth conditions

We used three *Zymoseptoria tritici *isolates for all experiments: Zt05 (Thygesen, Jørgensen, Jensen, & Munk, 2008), Zt09 (≙ IPO323ΔChr18, a derivate of the reference strain IPO323 (Goodwin et al., 2011) that lost chromosome 18 (Kellner et al., 2014)), and Zt10 (Stukenbrock, Banke, Javan‐Nikkhah, & McDonald, 2007) (Supporting Information Table S1). Fungal cells were inoculated from glycerol stocks onto YMS agar (0.4% [w/v] yeast extract, 0.4% [w/v] malt extract, 0.4% [w/v] sucrose, 2% [w/v] bacto agar) and grown at 18°C for 5 days. Single cells were grown in liquid YMS (200 rpm, 18°C) for 2 days and harvested by centrifugation (3,500 rpm for 10 min).

### 2.2 Plant infection experiments

We conducted all plant infection experiments in controlled plant growth chambers and inoculated 14‐day‐old seedlings of the winter wheat (*Triticum aestivum*) cultivar Obelisk (Wiersum Plantbreeding, Winschoten, Netherlands). Fungal inoculum was adjusted to 1 × 10^8^ cells/ml in 0.1% [v/v] Tween 20 (Roth, Karlsruhe, Germany) and brushed onto labeled areas (8 to 12 cm) of the second leaves. The same treatment without fungal cells was applied for mock controls. Plants were incubated for 48 hr at 22°C [day]/20°C [night] and 100% humidity with a 16‐hr light period. Subsequently, humidity was reduced to 70%. Plants were grown for three or four weeks after inoculation, depending on the experiment.

### 2.3 In planta phenotypic assays

To compare quantitative virulence of Zt05, Zt09, and Zt10 on wheat, we performed three independent, randomized infection experiments with blinded inoculation and evaluation. Inoculated leaf areas of 460 leaves (Zt05: 116, Zt09: 118, Zt10: 118, mock control: 108) were evaluated at 28 days post infection (dpi) by scoring the observed disease symptoms based on the percentage of leaf area covered by necrosis and pycnidia (Poppe, Dorsheimer, Happel, & Stukenbrock, 2015). We differentiated six categories (Supporting Information Figure S1): 0 (no visible symptoms), 1 (1%–20%), 2 (21%–40%), 3 (41%–60%), 4 (61%–80%), and 5 (81%–100%). Statistical differences were evaluated by Mann–Whitney *U* test considering differences significant if *p* ≤ 0.01.

To compare the temporal disease development, we manually inspected 40 inoculated leaves per isolate between 9 and 27 dpi and registered the occurrence of first visible symptoms every two days. Individual leaves were documented using a Leica S8APO equipped with a Leica DFC450 camera.

To localize and visualize the reactive oxygen species H_2_O_2 _within infected leaf tissue, we conducted 3,3′‐diaminobenzidine (DAB) staining (Thordal‐Christensen, Zhang, Wei, & Collinge, 1997) at 2, 4, 7–11, 14, 16, 18, and 21 dpi and quantified the reddish‐brown precipitate in cleared leaves which indicates an accumulation of H_2_O_2 _(Supporting Information Text S1). Leaves were documented prior and post staining.

### 2.4 Analysis of *Z. tritici *wheat infection by confocal microscopy

Development of *Z. tritici* isolates within and on the surface of wheat leaves was analyzed by confocal laser scanning microscopy. We harvested infected wheat leaves at 3–14, 17, 19–21, 24, 25, and 28 dpi and analyzed the interactions between fungal hyphae and wheat tissue. Likewise, we analyzed infected leaves to determine the infection stage of leaf samples used for RNA extraction (see below). In total, we studied 37 infected wheat leaves for Zt05, 34 for Zt09, and 30 for Zt10 (Supporting Information Table S1), analyzed at least 15 infection events per leaf sample by confocal microscopy, and created a total of 113 confocal image z‐stacks. Cleared leaf material was stained with wheat germ agglutinin conjugated to fluorescein isothiocyanate (WGA‐FITC) in combination with propidium iodide (PI) (Supporting Information Text S1 for staining protocol). Microscopy was conducted using a Leica TCS SP5 (Leica Microsystems, Germany) and a Zeiss LSM880 (Carl Zeiss Microscopy, Germany). FITC was excited at 488 nm (argon laser) and detected between 500 and 540 nm. PI was excited at 561 nm (diode‐pumped solid‐state laser) and detected between 600 and 670 nm. Analyses, visualization, and processing of image z‐stacks were performed using Leica Application Suite Advanced Fluorescence (Leica Microsystems, Germany), ZEN black and Zen blue (Carl Zeiss Microscopy, Germany), and AMIRA^® ^(FEI^TM^ Visualization Science Group, Germany). Animations of image *z*‐stacks are .avi format and can be played in VLC media player (available at http://www.videolan.org/vlc/).

### 2.5 Transcriptome analyses of *Z. tritici *isolates during wheat infection

Total RNA from *Z. tritici*‐infected wheat material collected during one infection experiment was isolated using the TRIzol™ reagent (Invitrogen, Karlsruhe, Germany) according to the manufacturer's instructions. One biological sample consists of material from three inoculated second leaves that were pooled and homogenized in liquid nitrogen. One hundred milligram of the resulting powder was used for RNA extraction. Because our analyses revealed isolate‐specific differences in the temporal infection development, we used independent sampling schedules for each isolate to be able to compare their transcriptomes during the same infection stage (Supporting Information Table S2). We collected infected leaf material at up to nine time points per isolate and assigned infection stages by examining central sections (1–2 cm) of each leaf by confocal microscopy (Supporting Information Figure S2). Per isolate and infection stage, the two most representative samples were selected as biological replicates for transcriptome sequencing (Table 1). Preparation of strand‐specific RNA‐seq libraries including polyA enrichment was performed at the Max Planck Genome Center, Cologne, Germany (http://mpgc.mpipz.mpg.de), using the NEBNext Ultra^TM^ Directional RNA Library Prep Kit for Illumina according to the manufacturer's protocol (New England Biolabs, Frankfurt/Main, Germany) with an input of 1 µg total RNA. Sequencing, performed using an Illumina HiSeq 2,500 platform, generated strand‐specific, 100‐base, single‐end reads with an average yield of 112 million reads per sample (Supporting Information Table S3).

**Table 1:** Summary of the stage‐specific transcriptomes (A–D) of the three *Zymoseptoria tritici* isolates

Isolate	Infection stage	Sample	Time point (dpi)	No. of filtered reads	No. of reads mapped to genome	% reads mapped to genome	No. of genes RPKM ≥2^a^	No. of genes RPKM ≥10^a^
Zt05^b^	A	Zt05_Ta_A_01	3	107,507,137	15,213,307	14.15	9,302	7,455
		Zt05_Ta_A_02		81,479,903	10,509,515	12.90		
	B	Zt05_Ta_B_01	8	113,732,295	15,057,425	13.24	9,404	7,623
		Zt05_Ta_B_02		128,271,704	14,856,314	11.58		
	C	Zt05_Ta_C_01	13	91,298,814	26,756,807	29.31	9,538	7,982
		Zt05_Ta_C_02		135,198,719	34,329,884	25.39		
	D	Zt05_Ta_D_01	20	86,100,462	41,871,582	48.63	9,585	7,914
		Zt05_Ta_D_02		119,101,106	76,123,086	63.91		
Zt09^c^	A	Zt09_Ta_A_01	4	129,342,007	5,868,572	4.54	9,435	7,482
		Zt09_Ta_A_02		92,711,865	5,582,561	6.02		
	B	Zt09_Ta_B_01	11	96,767,482	5,034,677	5.20	9,718	7,910
		Zt09_Ta_B_02		103,428,015	5,964,566	5.77		
	C	Zt09_Ta_C_01	13	121,529,652	31,264,373	25.73	9,892	8,220
		Zt09_Ta_C_02		93,253,633	27,790,773	29.80		
	D	Zt09_Ta_D_01	20	110,296,264	84,263,562	76.40	9,867	7,949
		Zt09_Ta_D_02		101,757,635	75,044,880	73.75		
Zt10^d^	A	Zt10_Ta_A_01	6	93,557,587	4,895,650	5.23	8,814	7,219
		Zt10_Ta_A_02		91,111,840	4,836,535	5.31		
	B	Zt10_Ta_B_01	11	94,828,793	5,951,869	6.28	9,068	7,407
		Zt10_Ta_B_02		110,255,245	7,896,227	7.16		
	C	Zt10_Ta_C_01	13	86,652,710	20,804,110	24.01	9,241	7,742
		Zt10_Ta_C_02		91,070,127	8,620,099	9.47		
	D	Zt10_Ta_D_01	24	98,493,807	29,939,216	30.40	9,062	7,308
		Zt10_Ta_D_02		93,690,628	34,607,371	36.94		

Note

Overview of RNA‐seq datasets including time point of sampling, number of sequenced reads post filtering, number of mapped reads, percentage of mapped reads, and numbers of transcribed genes.

a RPKM values were calculated using Cuffdiff2 and are normalized over all infection stages within the respective isolate (normalization method: geometric, dispersion method: per‐condition).

b 11,138 genes of IPO323 (94.08%) found by nucleotide blast for Zt05.

c 11,754 of the 11,839 genes predicted and annotated for IPO323 (Grandaubert et al., 2015); 85 genes located on chromosome 18 were not considered.

d 10,745 genes of IPO323 (90.76%) found by nucleotide blast for Zt10.

We assessed the quality of the sequencing data with *FastQC* v0.11.2 (http://www.bioinformatics.babraham.ac.uk/projects/fastqc/) and applied a stringent trimming and filtering protocol using *FASTX‐toolkit* v0.0.14 (http://hannonlab.cshl.edu/fastx_toolkit/) and *Trimmomatic* (Bolger, Lohse, & Usadel, 2014) v0.33 (Supporting Information Text S1 for details). The resulting 88‐bp reads were mapped against the genome of the respective isolate with *TopHat2* v2.0.9 (Kim et al., 2013). Read alignments were stored in SAM format, and indexing, sorting, and conversion to BAM format were performed using SAMtools v0.1.19 (Li et al., 2009). Relative abundance of transcripts for predicted genes was calculated in RPKM by *Cuffdiff2* v2.2.1 (Trapnell et al., 2013). Raw read counts per gene were estimated with *HTSeq* v0.6.1p1 (Anders, Pyl, & Huber, 2015). Gene coordinates in the Zt05 (Supporting Information Table S4) and Zt10 (Supporting Information Table S5) genomes were obtained by mapping the predicted genes of IPO323 using nucleotide BLAST alignments (*e*‐value cutoff 1*e* ^−3^, identity ≥90%, query coverage 90%–110%). Differential gene expression analyses between *Z. tritici *infection stages and isolates were performed in R (R Core Team, 2017) using the Bioconductor package *DESeq2 *v1.10.1 (Love, Huber, & Anders, 2014). Significant signals of differential expression for the most strongly changing genes were determined with *p*_adj_ ≤0.01 and |log_2_ fold change ≥2| as recommended for RNA‐seq experiments with a low number of replicates (Schurch et al., 2016). The R package topGO (Alexa, Rahnenführer, & Lengauer, 2006) was used to perform Gene Ontology (GO) term enrichment analyses. *p* Values for each GO term (Grandaubert, Bhattacharyya, & Stukenbrock, 2015) were calculated using Fischer's exact test applying the topGO algorithm “weight01” considering GO term hierarchy. We reported categories significant with *p* ≤ 0.01 for the ontology “Biological Process.” PFAM domain enrichment analyses were performed using a custom python script, and *p* values were calculated using chi‐square tests.

To analyze genomic distances between differentially expressed genes and transposable elements (TEs), we annotated TEs as described in Grandaubert et al. (2015) for Zt05 (Supporting Information Table S6) and Zt10 (Supporting Information Table S7) and used the published TE annotation of IPO323 for Zt09 (Grandaubert et al., 2015). Distances between genes of interest and the closest annotated TEs were calculated with *bedtools* v2.26 (Quinlan & Hall, 2010). Likewise, we used ChIP‐seq peak data (Schotanus et al., 2015) to calculate distances between genes and the closest H3K9me3 and H3K27me3 peaks. Statistical analyses were performed in R. For a detailed overview, see Supporting Information Text S1.

### 2.6 Pulsed‐field gel electrophoresis

A non‐protoplast protocol (Supporting Information Text S1) was used to produce DNA plugs for separation of small chromosomes (~0.2 to 1.6 Mb) by pulsed‐field gel electrophoresis (PFGE) (Stukenbrock et al., 2010). Chromosomal DNA of *Saccharomyces cerevisiae* (Bio‐Rad, Munich, Germany) was used as standard size marker. Gels were stained in 1 µg/ml ethidium bromide solution, and chromosome bands were detected with Thyphoon Trio™ (GE Healthcare, Munich, Germany).

### 2.7 De novo genome assemblies of *Z. tritici* isolates Zt05 and Zt10 and synteny analyses

DNA of Zt05 and Zt10 was extracted from single cells using the CTAB extraction protocol (Allen, Flores‐Vergara, Krasynanski, Kumar, & Thompson, 2006) and used as input to prepare Pacific Biosciences (PacBio) SMRTbell libraries. Single‐molecule real‐time (SMRT) sequencing was performed on four SMRT cells and run on a PacBio RS II instrument at the Max Planck Genome Center. Genome assemblies of Zt05 and Zt10 based on the generated reads were done as previously described (Plissonneau, Stürchler, & Croll, 2016) using *HGAP* (Chin et al., 2013) v3.0 included in *SMRTanalysis suite* v2.3.0. For further details, see Supporting Information Text S1.

Synteny of *Z. tritici* reference strain IPO323 and the Zt05 and Zt10 unitigs was compared using SyMAP v4.2 (Soderlund, Bomhoff, & Nelson, 2011) applying default settings and considering all unitigs ≥1,000 bp (Zt05) and ≥10,000 bp (Zt10). To estimate the amount of unique DNA in Zt05 and Zt10 in comparison with IPO323 and Zt09, respectively, we generated pairwise genome alignments with *Mugsy *v1.r2.2 (Angiuoli & Salzberg, 2011) applying default settings. Alignments were analyzed using a custom python script to extract unique DNA blocks with a minimum length of 1 bp.

## 3 RESULTS

### 3.1 The three *Z. tritici* isolates Zt05, Zt09, and Zt10 exhibit high levels of genomic variation

We selected three *Z. tritici* isolates Zt05, Zt09, and Zt10 that belong to genetically different clades within the species (Grandaubert, Dutheil, & Stukenbrock, 2017) but produce the same amount of disease symptom on the susceptible wheat cultivar Obelisk (see below). These isolates were previously collected in Denmark, the Netherlands, and Iran, respectively (Supporting Information Table S1), and vary in their tolerance to abiotic stressors (Supporting Information Text S1, Figure S3, Table S8). A previous population genomic study using the 39.7 Mb genome of the isolate IPO323 (Goodwin et al., 2011) as reference addressed the extent of genetic variation in these isolates and identified 500,177 single nucleotide polymorphisms (SNPs) in Zt05 and 617,431 SNPs in Zt10, indicating a considerable genetic distance between the three isolates (Grandaubert et al., 2017).

Previous comparative genome analyses have shown that *Z. tritici* is highly polymorphic at the level of chromosome structure and gene content, including chromosomal rearrangements, genomic orphan regions, and isolate‐specific gene presence/absence patterns (Hartmann et al., 2017; Plissonneau et al., 2016; Plissonneau, Hartmann, & Croll, 2018). To further assess variation in genome structure and content, we performed a karyotype analysis by pulsed‐field gel electrophoresis (PFGE) and generated high‐quality genome assemblies based on long‐read SMRT sequencing of Zt05 and Zt10 (Supporting Information Table S9). The PFGE analyses revealed extensive variation in the karyotypes of the three isolates with no small chromosomes of the same size (Supporting Information Figure S4). The PFGE results suggest that Zt05 and Zt10 possess at least seven and four putative accessory chromosomes, respectively, and show length polymorphisms of the smallest core chromosomes 12 and 13 consistent with variation reported in a previous study (Mehrabi, Taga, & Kema, 2007). In spite of the pronounced differences in genome structure, alignment of the three high‐quality genome assemblies showed a high extent of synteny. By whole‐chromosome synteny analyses using SyMAP, we identified large syntenic DNA blocks for all 21 chromosomes of IPO323 in the Zt05 assembly, while Zt10 lacked homologs of chromosomes 18, 20, and 21 (Supporting Information Figure S5 and Table S9).

To identify the genes that are shared between Zt05, Zt09, and Zt10, we performed nucleotide BLAST analyses using the coding sequences of the 11,839 annotated genes of the reference IPO323 as input (Grandaubert et al., 2015). We identified 11,138 IPO323 genes (94.08%) in Zt05 and 10,745 (90.76%) in Zt10. The gene presence/absence patterns correlate with the absence of large syntenic DNA blocks of chromosomes 18, 20, and 21 in Zt10 (Supporting Information Figure S5B and Table S9). Consequently, 91% of genes on core chromosomes are shared, while only 49% (313 of 643) of the genes located on accessory chromosomes are present in all three isolates (Supporting Information Table S9). Similarly, only 85% (370 of 434) of the previously identified genes encoding candidate secreted effector proteins (CSEPs) (Stukenbrock & Dutheil, 2018) were found in all isolates, indicating that the effector repertoire of *Z. tritici* is characterized by presence/absence polymorphisms. In total, 10,426 genes were present in the three isolates and considered to be *Z. tritici* core genes that we included in our further analyses (Supporting Information Table S10). In summary, the genome comparison of Zt05, Zt09, and Zt10 shows a high extent of variation at single nucleotide positions as well as structural variation including differences in the total gene content that is typical in natural isolates of this highly polymorphic pathogen.

### 3.2 Virulence of the three *Z. tritici* isolates is very similar but disease develops at different speeds

**Figure 1:**
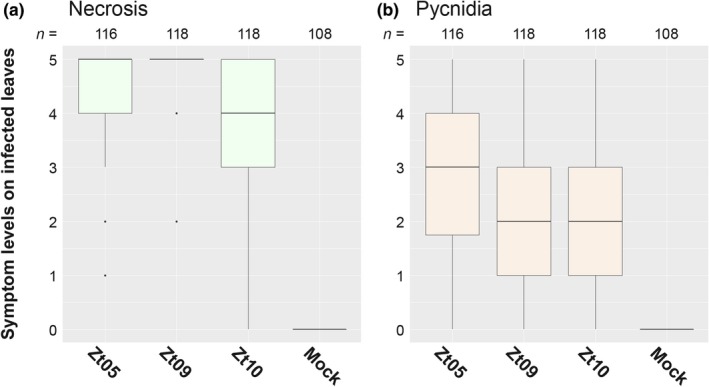
In planta phenotypic assay demonstrates similar pycnidia levels of *Zymoseptoria tritici* isolates on the susceptible wheat cultivar Obelisk. Quantitative differences in (a) necrosis and (b) pycnidia coverage of inoculated leaf areas were manually assessed at 28 days post inoculation based on six symptom levels: 0 (without visible symptoms), 1 (1% to 20%), 2 (21% to 40%), 3 (41% to 60%), 4 (61% to 80%), and 5 (81% to 100%). The three isolates caused different levels of necrosis (two‐sided Mann–Whitney *U* tests, *p* ≤ 0.0048), but pycnidia levels were not different (two‐sided Mann–Whitney *U* tests, *p* ≥ 0.034)

We compared the virulence phenotypes of Zt05, Zt09, and Zt10 on the highly susceptible wheat cultivar Obelisk and evaluated infections 28 days post inoculation (dpi) by categorizing the percentage of leaf area affected by necrosis (Figure 1a and Supporting Information Figure S6) and covered with pycnidia, the asexual fruiting bodies (Figure 1b and Supporting Information Figure S6). Although we observed different levels of necrosis (two‐sided Mann–Whitney *U* tests, *p* ≤ 0.0048), we found no significant differences in the pycnidia levels of the three isolates (two‐sided Mann–Whitney *U* tests, *p* ≥ 0.034) (Supporting Information Figure S7). As previously observed (Stewart & McDonald, 2014), quantitative necrosis and pycnidia levels were only weakly linked and coverage of necrotic lesions with pycnidia was highly variable (Supporting Information Figure S1). Hence, for quantitative virulence of *Z. tritici,* the production of pycnidia is considered the primary measure as it directly reflects reproductive fitness rather than differences in host sensitivity, for example, sensitivity toward pathogen toxins that contribute to necrosis (Stewart & McDonald, 2014). Based on the observed pycnidia levels at 28 dpi, we conclude that the three *Z. tritici* isolates have similar virulence phenotypes on the wheat cultivar Obelisk.

**Figure 2:**
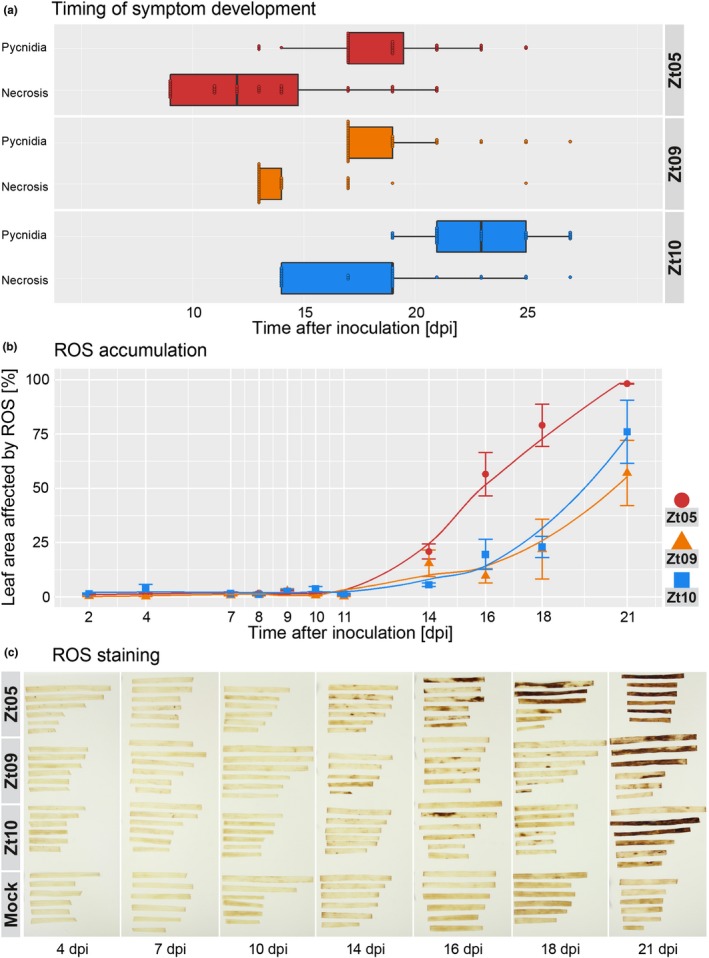
Timing of disease symptom development and H_2_O_2_ accumulation varies between wheat leaves infected with different *Zymoseptoria tritici* isolates. (a) Temporal disease progression for infections with Zt05, Zt09, and Zt10 was measured by manual screening for the first occurrence of necrotic spots and pycnidia. For each isolate, 40 leaves of the wheat cultivar Obelisk were inoculated and tested. No disease developed on seven of the leaves inoculated with Zt10. (b) Reactive oxygen accumulation in infected leaves was visualized by staining with 3,3′‐diaminobenzidine and quantified by analysis of six scanned leaf images per isolate and time point. Mean values of percentage leaf area affected by ROS are plotted. Error bars indicate standard errors of the mean. (c) Dark red‐brown precipitate indicates H_2_O_2 _accumulation and appeared first in leaves infected with Zt05 in leaf areas beginning to undergo necrosis

Next, we investigated whether disease symptoms develop at a similar pace. We monitored temporal disease progress by screening the leaves every other day for visible necrotic spots and pycnidia. Leaves inoculated with Zt05 and Zt09 showed necrosis and pycnidia significantly earlier than leaves inoculated with Zt10 (one‐sided Mann–Whitney *U* tests, *p* ≤ 7.73 × 10^−8^) (Figure 2a). The median onset of necrosis caused by Zt09 occurred one day after that caused by Zt05 and is significantly later (one‐sided Mann–Whitney *U* test, *p* = 0.0089), although the first pycnidia of both isolates developed at the same time (two‐sided Mann–Whitney *U* test, *p* = 0.9455). Thus, although the three *Z. tritici* isolates produced similar quantities of pycnidia in Obelisk, disease develops at different speeds.

Reactive oxygen species (ROS) play a central role in plant pathogen defense by acting as signaling molecules after pathogen recognition and activating defense responses (Heller & Tudzynski, 2011). We aimed to determine whether the observed differences in temporal disease development of *Z. tritici* isolates reflect a temporal variation in host response. In some wheat cultivars, a rapid upregulation of defense‐related genes is part of the early responses to *Z. tritici* infections (Ma, Keller, McDonald, Palma‐Guerrero, & Wicker, 2018; Ray, Anderson, Urmeev, & Goodwin, 2003) while only little transcriptional changes and even the downregulation of such genes were found in the cultivars Riband and Sevin during the asymptomatic phase (Rudd et al., 2015; Yang, Li, & Jørgensen, 2013). Since previous luminol‐based assays for ROS quantification in wheat tissue failed to produce consistent results (Schoonbeek et al., 2015), we visualized the ROS H_2_O_2_ by diaminobenzidine (DAB) staining and quantified accumulation by image analysis of the stained leaves. H_2_O_2 _is involved in restricting colonization of resistant wheat cultivars but also plays an important role during necrotrophic fungal growth in compatible infections (Shetty et al., 2003, 2007). We observed ROS accumulation coinciding with the onset of necrosis (Figure 2c and Supporting Information Figure S8). However, 11 to 14 days after inoculation, we observed no ROS (Figure 2b,c), possibly because concentrations were low and not detectable. This suggests no or very weak activation of immune responses during this phase of infection in Obelisk. Consistent with the faster disease progress, ROS accumulates earlier in leaves infected with Zt05 than Zt09 and Zt10 (Figure 2b). Thereby, the timing of ROS accumulation in response to the three *Z. tritici* isolates is consistent with the observed differences in the temporal development of disease symptoms in infected wheat tissue.

### 3.3 *Zymoseptoria tritici* infection is characterized by four core developmental stages

**Figure 3:**
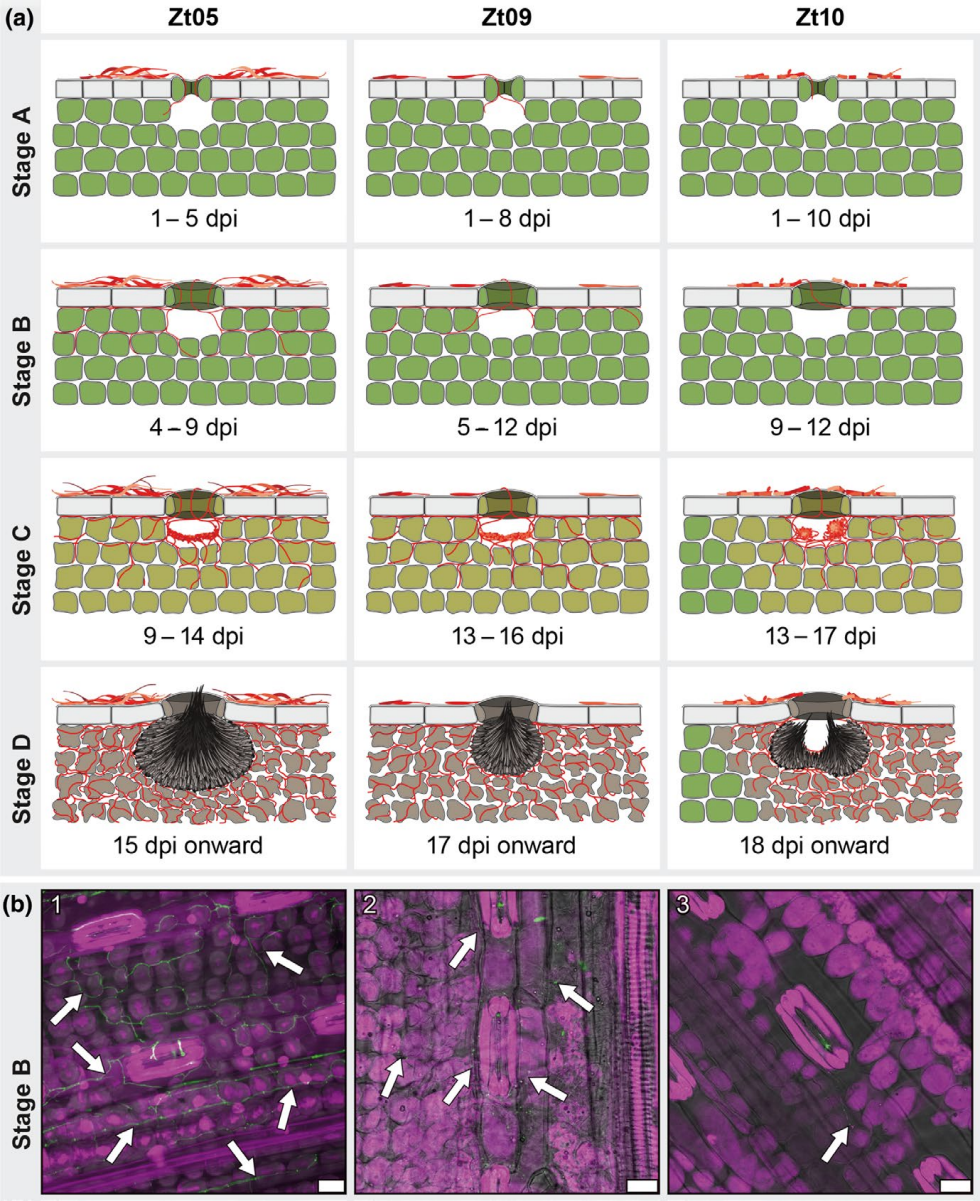
*Zymoseptoria tritici* wheat infections are characterized by four distinct infection stages and isolate‐specific infection development. (a) Schematic drawings of the key features that characterize the four infection stages of *Z. tritici* and illustrate the infection phenotypes of isolates Zt05, Zt09, and Zt10 on the wheat cultivar Obelisk. (b) Micrographs showing *Z. tritici *hyphae (arrows) during biotrophic growth inside wheat leaves. Maximum projections of confocal image z‐stacks. Nuclei and wheat cells are displayed in *purple* and fungal hyphae or septa in *green*. The panel shows biotrophic colonization of (1) isolate Zt05 at 7 dpi, (2) Zt09 at 11 dpi, and (3) Zt10 at 9 dpi. Scale bars = 25 µm

Next, we aimed to morphologically characterize host colonization of the three *Z. tritici* isolates. We conducted detailed confocal microscopy analyses in which we scanned 101 leaves harvested between 3 and 28 days after inoculation (Supporting Information Table S1). Analyses of large z‐stacks of longitudinal optical sections allowed us to infer the spatial and temporal fungal colonization on and in infected leaves. First, we focused on the commonalities in host colonization shared by the isolates. We identified a sequence of four stages that we define as the core infection program of *Z. tritici* (Figure 3; Supporting Information Text S1).

Stage A, or infection establishment, involves the penetration of wheat leaf tissue by fungal hyphae. After inoculation, fungal cells germinate on the leaf surface. Germ tubes of *Z. tritici* develop into infection hyphae, some of which grow in the direction of stomata while others grow epiphytically (Figure 3a stage A; Supporting Information Animations S1 and S2). During stomatal penetration and in the substomatal cavities, infection hyphae grow in tight contact with the guard cells.

Stage B refers to the symptomless, biotrophic intercellular colonization of the wheat mesophyll (Figure 3a stage B; Supporting Information Animations S3 and S4). During this stage, the pathogen explores host tissue without recognition by the host immune system, as indicated by the absence of an accumulation of ROS in infected tissue (Figure 2b,c). Hyphae first grow in the interspace of epidermis and mesophyll, where they spread via the grooves between adjacent epidermal cells before deeper mesophyll cell layers are colonized.

Infection stage C comprises the transition from biotrophic to necrotrophic growth when the first disease symptoms develop (Figure 3a stage C). For asexual reproduction, *Z. tritici* requires large amounts of nutrients that are released from dead host cells. Fungal hyphae branch extensively and colonize all mesophyll layers, with hyphae growing around individual plant cells as they die off. Primary structures of pycnidia start to develop (Supporting Information Animations S5–S7), and ring‐like scaffolds form in substomatal cavities where hyphae align and build stromata that give rise to conidiogenous cells.

Necrotrophic colonization and asexual reproduction characterize infection stage D (Figure 3a stage D). Hyphae colonize an environment that is nutrient‐rich but also hostile due to highly abundant ROS (Figure 2b). Substomatal cavities are occupied by mature pycnidia that harbor asexual pycnidiospores, which are extruded through stomatal openings. The adjacent mesophyll tissue is heavily colonized by hyphae wrapping around collapsed host cells (Supporting Information Animations S8 and S9), possibly to improve the acquisition of nutrients and protect resources from competing saprotrophs.

Although the four infection stages of *Z. tritici* can be clearly distinguished, infections by different cells of one isolate are not completely synchronized; different infection stages can be present simultaneously on the same inoculated leaf to some extent.

### 3.4 Highly differentiated infection phenotypes of the three *Z. tritici* isolates on Obelisk wheat

While we clearly recognized the four core infection stages for the isolates Zt05, Zt09, and Zt10, we also observed differences (Supporting Information Text S1) that mainly relate to the timing of transitions between the stages and the extent of fungal proliferation. During stage A, infection hyphae of Zt05 enter stomata between 1 and 5 dpi, whereas germ tube formation and stomatal penetration for Zt09 and Zt10 occur later (Figure 3a stage A). We also observed strong epiphyllous growth for Zt05 and, in contrast to the two other isolates, the frequent occurrence of several hyphae entering a single stoma (Figure 3a stage A: Zt05). Stomatal penetrations are evenly distributed for Zt05 and Zt09 in inoculated leaf areas, while Zt10 penetrations are more clustered, leading to patchy infections (Figure 3a stages C, D: Zt10). Zt05 is also the first isolate switching to necrotrophic growth (9 to 14 dpi), followed by Zt09 (13 to 16 dpi) and Zt10 (13 to 17 dpi) (Figure 3a stage C). The isolates enter stage D in the same order. Furthermore, Zt10 forms two pycnidia in one substomatal cavity (Figure 3a stages C and D: Zt10; Supporting Information Animation S7) more often than Zt05 and Zt09.

The most striking difference between the isolates is however the extent of biotrophic colonization during stage B (Figure 3b). Zt05 develops expanded biotrophic hyphal networks with long “runner” hyphae growing longitudinally between epidermis and mesophyll (Figures 3a stage B: Zt05, 3b1; Supporting Information Animation S3). Zt09 produces fewer hyphae that are located mainly between epidermis and mesophyll and in the first mesophyll cell layer (Figures 3a stage B: Zt09, 3b2). Biotrophic growth of Zt10 is strongly reduced and limited to the mesophyll cells adjacent to substomatal cavities (Figures 3a stage B: Zt10, 3b3). Intrafoliar growth by Zt10 beyond that is only found during the lifestyle switch and later. During asymptomatic colonization, *Z. tritici* depends on successful evasion of host immunity (Jones & Dangl, 2006) and acquires energy initially mainly from stored lipids and only later from host‐derived nutrients (Rudd et al., 2015). Delayed stomatal penetration and reduced internal leaf colonization increase the role of the epiphytic phase where the pathogen is exposed to environmental influences and control measures but could still be transferred to a more favorable host environment (Fones et al., 2017). We speculate that the different extent of fungal growth reflects colonization of different niches associated with the host tissue, deviating strategies to avoid host recognition as well as distinct capabilities to store lipids and exploit the limited nutrient resources during biotrophic colonization.

Taken together, the infection development of the studied *Z. tritici* isolates is highly divergent, although the final infection outcome is the same (Figure 1). Thereby, instead of one strictly defined infection program, *Z. tritici* exhibits a variety of host–pathogen interactions that represent equally successful strategies for reproduction in a susceptible wheat cultivar. In particular, we find that infection development of the wheat pathogen can be highly flexible with respect to the timing of the lifestyle transition and the spatial distribution of infecting hyphae inside host tissue.

### 3.5 Generation of isolate‐ and stage‐specific transcriptomes based on confocal microscopy analyses

Given the morphological and temporal differences in infection development, we next asked how gene expression profiles differ between the *Z. tritici* isolates Zt05, Zt09, and Zt10 during wheat infection. Previous studies have demonstrated transcriptional reprogramming in *Z. tritici* during infection (Brunner, Torriani, Croll, Stukenbrock, & McDonald, 2013; Kellner et al., 2014; Keon et al., 2007; Palma‐Guerrero et al., 2016; Rudd et al., 2015; Yang et al., 2013) and shown different transcriptional programs of strains that differ in their virulence phenotypes (Palma‐Guerrero et al., 2017). The transcriptome studies involving different isolates were however limited by the fact that the sequenced samples were collected at predefined time points. Our observations of significant temporal variation in infection development of *Z. tritici* suggest that predefined sampling time points may be biased and in fact lead to the comparison of transcriptomes of different developmental infection stages.

To account for the temporal variation between Zt05, Zt09, and Zt10, we combined confocal laser scanning microscopy and RNA‐seq and conducted microscopy analyses of tissue from the wheat leaves used for RNA extraction and transcriptome sequencing. Infected leaves were collected at up to nine time points per isolate, and samples for transcriptome sequencing were selected based on their morphological infection stage (Supporting Information Figure S2; Table 1, Supporting Information Table S2). Thereby, we generated stage‐specific RNA‐seq datasets corresponding to the four core infection stages, allowing us to compare the isolate‐specific expression profiles at the same stage of infection development.

We obtained 89.2 to 147.5 million single‐end, strand‐specific reads per replicate (total >2.7 billion reads) that were quality trimmed and filtered. Between 4.54% (early infection) and 76.4% (late infection) of the reads could be mapped to the genome of the respective isolate, reflecting the infection stage‐specific amount of fungal biomass (Table 1, Supporting Information Table S3; Text S1). Across all isolates, transcriptomes of stages A and B, representing biotrophic growth, cluster together and are clearly different from transcriptomes of stages C and D that likewise cluster and represent necrotrophic growth of *Z. tritici* (Supporting Information Figures S9 and S10). Exploring the transcriptome datasets based on gene read counts shows the greatest variation of biological replicates for Zt10 at stage C (Supporting Information Figure S11), possibly reflecting variability in the infection development of the two biological replicates.

### 3.6 Core *Z. tritici* transcriptional program during wheat infection

**Figure 4:**
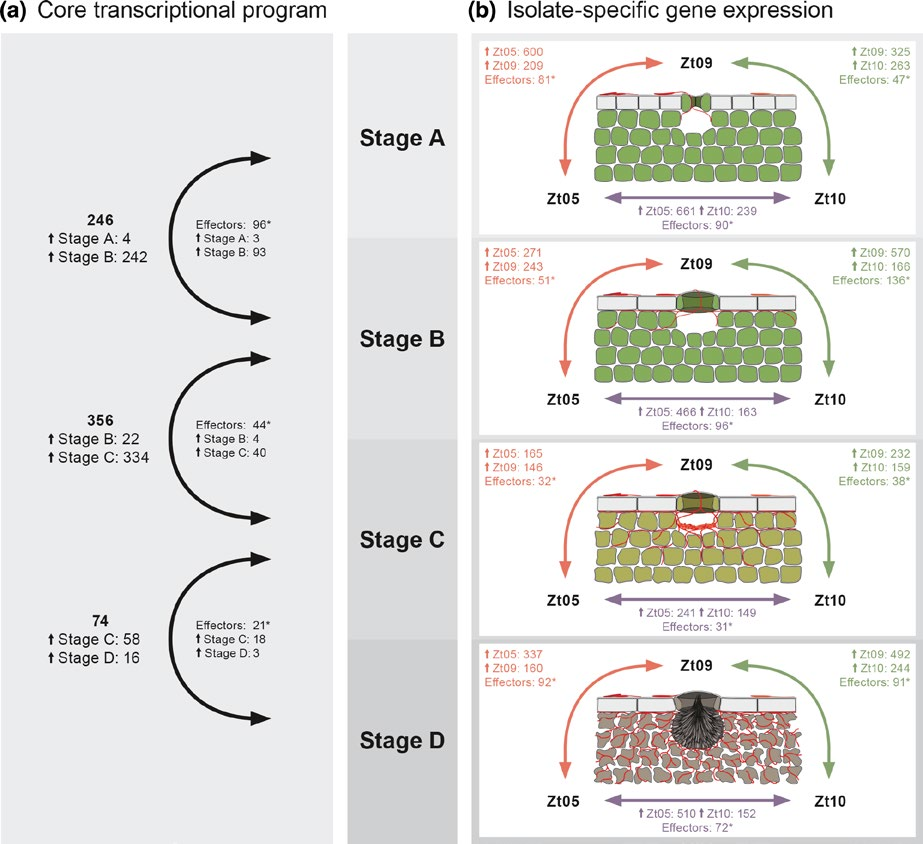
*Zymoseptoria tritici* core transcriptional program during wheat infection and isolate‐specific expression during the four infection stages. Numbers of significantly differentially expressed genes across all isolates (a) between the four core *Z. tritici* infection stages and (b) between the isolates within the infection stages (between Zt05 and Zt09: orange arrows, between Zt05 and Zt10: purple arrows, between Zt09 and Zt10: green arrows). Small arrows (↑) with stage or isolate names indicate the number of genes specifically up‐regulated during that stage or in that isolate for the respective comparison. Differential gene expression analyses performed with DESeq2. Genes were considered to be significantly differentially expressed if *p*_adj_ ≤ 0.01 and |log_2_ fold change| ≥ 2. *Indicates significant enrichment of effector candidates among differentially expressed genes (Fischer's exact tests, *p* < 0.001). Effector candidates encode secreted proteins putatively involved in modulating molecular host‐pathogen interactions (Lo Presti et al., 2015)

The mean expression of genes located on accessory chromosomes was 6‐ to 20‐fold lower than the expression levels of genes located on core chromosomes (Supporting Information Table S11). We performed differential gene expression analyses to compare transcription of the 10,426 *Z. tritici* core genes. We hypothesized that genes involved in stage‐specific infection development and transitions between the four core stages have dynamic, stage‐specific expression profiles that are shared among the three *Z. tritici* isolates. In total, we identified only 597 genes (5.6%) that fulfilled these two criteria: differential expression between infection stages (DESeq2, *p*_adj_ ≤0.01, |log_2_ fold change| ≥2) and shared expression kinetics—and hence represent putative determinants of the *Z. tritici* core infection stages (Figure 4a). Interestingly, 79 of these genes were differentially expressed between several infection stages, suggesting dynamic, wave‐like expression kinetics (Supporting Information Figure S12).

A total of 246 genes were differentially expressed (Supporting Information Table S12) between stage A and stage B. The vast majority of these (242) were up‐regulated in stage B and are enriched in genes encoding proteins without a functional prediction (146) and with Gene Ontology (GO) groups involved in proteolysis (GO:0006508; 27 genes) and amino acid transmembrane transport (GO:0003333; five genes) (*p* < 0.01, Fischer's exact test). PFAM domain analysis further shows enrichment of genes encoding cytochrome P450‐like and polyketide synthase‐like proteins that possibly play a role in the production of secondary metabolites (*p* < 0.001, *χ* ^2^ test).

Comparing biotrophic growth and the transition to necrotrophic growth, only 22 genes are up‐regulated in the same manner in the three isolates in stage B while 334 genes are significantly induced during stage C (Supporting Information Table S13). Genes up‐regulated during the necrotrophic stage are enriched with GO groups involved in metabolic processes (GO:0008152; 97 genes), in particular L‐arabinose metabolic processes (GO:0046373; four genes), and transmembrane transport (GO:0055085; 25 genes). Similarly, the PFAM analysis shows an enrichment of genes encoding transporters; CAZymes including different groups of glycosyl hydrolases, serine hydrolases, alpha‐L‐arabinofuranosidases, and cutinases that play important roles as plant tissue and cell wall‐degrading enzymes (Kubicek, Starr, & Glass, 2014); polyketide synthases; and cytochrome P450s. These transcriptional changes reflect the previously described metabolic reprogramming of *Z. tritici* during the transition from biotrophic to necrotrophic growth (Rudd et al., 2015). Instead of feeding from intracellular lipids, the fungus switches to utilize plant‐derived nutrients (Rudd et al., 2015) and rapidly develops large hyphal networks and the primal structures of the asexual pycnidia in the infected wheat mesophyll tissue.

Between stages C and D, only 74 genes were differentially expressed (Supporting Information Table S14) indicating overall similar transcription profiles during the two necrotrophic stages. Among the 58 genes up‐regulated in stage C, we identified GO groups involved in arabinan metabolic processes (GO:0031221; one gene) and an enrichment of PFAM domains related to cytochrome P450s, polyketide synthases, hydrophobic surface‐binding protein A, tyrosinases, and to beta‐ketoacyl‐ACP synthases, which are known to be involved in fatty acid production and the generation of new cell membrane. Only 16 genes were significantly up‐regulated in all three isolates during the transition from stage C to stage D, which is when the pycnidia mature. These genes are predicted to encode proteins similar to CAZymes, transporters, and proteins containing RNA‐binding domains. Upregulation of a secreted catalase‐like protein‐encoding gene shows the importance of ROS detoxification, which is highly abundant in necrotic leaf tissue as shown by DAB staining (Figure 2c).

In summary, we identified a small number of genes that show the same expression dynamics in the three isolates suggesting that the transitions between the four stages of the core infection program of *Z. tritici* are facilitated by a comparably small set of core genes. These genes may be involved in determining the stage‐specific infection development and include candidates for core virulence determinants (see below).

### 3.7 Core biotrophic and necrotrophic effector candidates with shared expression profiles in *Z. tritici* isolates

Given their importance in plant–pathogen interactions, we particularly focused our analyses on genes encoding candidate secreted effector proteins (CSEPs) (Lo Presti et al., 2015). *Zymoseptoria tritici* CSEP‐encoding genes were previously predicted (Stukenbrock & Dutheil, 2018) using the machine learning approach EffectorP (Sperschneider et al., 2015) and are significantly enriched among the core differentially expressed genes (*p* ≤ 2.7 × 10^−13^, Fischer's exact tests) (Figure 4a), indicating highly dynamic transcription of core effectors during all stages of wheat infection. We filtered the differentially expressed CSEP genes according to their expression profiles (Supporting Information Figures S13 and S14) to identify putative key genes facilitating biotrophic and necrotrophic growth in wheat (Tables 2 and 3).

**Table 2:** *Zymoseptoria tritici* core biotrophic effector candidate genes

Criteria	Significantly up‐regulated at stage B			
	Low/no expression during stage C and D			
	RPKM stage B > RPKM stage C in Zt05 and Zt09			
Expression profiles	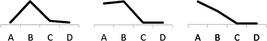			
Putative functions	Bypass host recognition during biotrophic colonization			
Candidate genes	25 (Supporting Information Figure S13)			
Candidates encoding hypothetical proteins	23 (Supporting Information Table S15)			
Candidates with predicted function	**Gene**	**Function/Functional prediction**	**PFAM domains**	**GO terms associated**
	*MgNLP *(Motteram et al., 2009) *Zt09_chr_13_00229*	Necrosis and ethylene‐inducing peptide 1‐like protein MgNLP (Kettles, Bayon, Canning, Rudd, & Kanyuka, 2017; Motteram et al., 2009)	PF05630	GO:0008150; GO:0003674; GO:0005575
	*Zt09_chr_2_00129*	Hypothetical protein, secreted phospholipase A2 precursor	PF06951	GO:0004623; GO:0005509; GO:0005576; GO:0016042

Note

Summary of core *Z. tritici* biotrophic effector candidate genes that were identified based on their specific expression profiles within the *Z. tritici *core transcriptional program during wheat infection. Functional annotation, PFAM, and GO term information from Grandaubert et al. (2015).

**Table 3:** *Zymoseptoria tritici* core necrotrophic effector candidate genes

Criteria	Significantly up‐regulated at stage C			
	Low/no expression during stage A and B			
Expression profiles	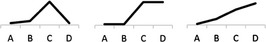			
Putative functions	Facilitate transition from biotrophy to necrotrophy			
	Induction of necrosis			
Candidate genes	35 (Supporting Information Figure S14)			
Candidates encoding hypothetical proteins	24 (Supporting Information Table S16)			
Candidates with predicted function	**Gene**	**Functional prediction**	**PFAM domains**	**GO terms associated**
	*Zt09_chr_3_00584*	PCWDE, similar to alpha‐1, Glycosyl transferases group 1	PF00128; PF00534; PF08323	GO:0003824; GO:0043169; GO:0005975; GO:0009058
	*Zt09_chr_9_00308*	PCWDE, similar to carbohydrate‐binding module family 63 protein, expansin‐like	—	GO:0008150; GO:0003674; GO:0005575
	*Zt09_chr_3_01063*	PCWDE, similar to pectate lyase	PF00544	GO:0008150; GO:0003674; GO:0005575
	*Zt09_chr_12_00112*	cutinase‐like protein	PF01083	GO:0050525; GO:0016787; GO:0005576; GO:0008152
	*Zt09_chr_2_00663*	similar to Chain A, cutinase‐like protein	PF01083	GO:0050525; GO:0016787; GO:0005576; GO:0008152
	*Zt09_chr_2_01151*	PCWDE, similar to acetyl xylan esterase,	PF01083	GO:0016787; GO:0008152
	*Zt09_chr_6_00446*	PCWDE,similar to acetyl xylan esterase	PF01083	GO:0016787; GO:0008152
	*Zt09_chr_10_00107*	PCWDE, similar to glycoside hydrolase family 12 protein	PF01670	GO:0008810; GO:0004553; GO:0000272
	*Zt09_chr_2_01205*	PCWDE, similar to putative extracellular cellulase CelA/allergen Asp F7‐like protein	PF03330	GO:0008150; GO:0003674; GO:0005575
	*Zt09_chr_6_00626*	similar to metalloprotease	PF05572	GO:0008237
	*Zt09_chr_9_00038*	similar to hydrophobin	PF06766	GO:0005576

Note

PCWDE: putative plant cell wall‐degrading enzyme.

Summary of core *Z. tritici* necrotrophic effector candidate genes that were identified based on their specific expression profiles within the *Z. tritici *core transcriptional program during wheat infection. Functional annotation, PFAM, and GO term information from Grandaubert et al. (2015).

During symptomless stage B, 78 CSEP genes are specifically up‐regulated, highlighting that a large suite of *Z. tritici* effectors is induced after stomatal penetration and is required for biotrophic colonization of wheat mesophyll. By filtering the up‐regulated genes for lower expression during stage C (RKPM stage B > RPKM stage C in Zt05 and Zt09), we narrowed down these genes to 25 core biotrophic CSEP genes (Table 2, Supporting Information Table [Link], [Link]; Figure S13) that mostly encode hypothetical proteins. In comparison with Zt05 and Zt09, expression of the biotrophic effectors in Zt10 is in general lower, possibly reflecting the strongly limited biotrophic colonization of this isolate (Figure 3b).

Thirty five CSEP genes are specifically up‐regulated at stage C (Supporting Information Figure S14) and represent candidates for necrotrophic core effectors (Table 3, Supporting Information Table S16). These genes may be involved in the transition from biotrophic to necrotrophic growth and the induction of necrosis. Nine CSEP genes encode putative plant cell wall‐degrading enzymes and cutinase‐like proteins, demonstrating that the lifestyle switch to necrotrophy involves intensified degradation of plant tissue and cell wall components. The gene *Zt09_chr_9_00038* encodes a putative hydrophobin; hydrophobins are small fungal‐specific proteins with various functions (Aimanianda et al., 2009; Wösten, 2001), i.a. as toxins in plant–pathogen interactions (Takai, 1974). Likewise strongly induced during the transition to necrotrophy is the gene *Zt09_chr_7_00263* that encodes a putative secreted metalloprotease, which are known fungal virulence factors in animal and plant pathogens (Karimi Jashni et al., 2015; Naumann, Wicklow, & Price, 2011; O'Connell et al., 2012; Vu et al., 2014).

In summary, our comparative approach allowed us to identify a set of core *Z. tritici *effector candidates, which are consistently expressed during infection of the cultivar Obelisk in the different genetic background of Zt05, Zt09, and Zt10.

### 3.8 Isolate‐specific transcriptional changes during wheat infection

The 597 genes that we identified as differentially expressed between the stages show the same expression profile in each of the three isolates and we consider them as part of the core *Z. tritici* transcriptional infection program. However, we observed that transcript levels of many genes strongly deviate between the isolates during the specific infection stages.

To further study how the infection phenotypes of the *Z. tritici* isolates relate to differences in gene expression, we compared expression profiles during the infection stages (Figure 4b). In total, 2,377 (~22.8%) of the 10,426 shared genes are differentially expressed between the *Z. tritici *isolates during wheat infection (Table 4, Supporting Information Tables  S17, S19) reflecting the extent of spatial, temporal, and quantitative differences in development that we observe by confocal microscopy (Figure 3a). A similar genome portion is differentially expressed in Swiss isolates during infection of the cultivar Drifter (Palma‐Guerrero et al., 2017) indicating a high extent of functional redundancy and flexibility in the infection program of *Z. tritici*.

**Table 4:** Genes with isolate‐specific expression profiles during wheat infection

Genes differentially expressed between isolates^a^	2,377 genes in total		
	22.8% of all 10,426 core genes		
	**Comparison (all four stages)**	**DE genes** b	**Regulation**
	Zt05‐Zt09	1,311	↑Zt05: 917 ↑Zt09: 436
	Zt05‐Zt10	1,482	↑Zt05: 1086 ↑Zt10: 412
	Zt09‐Zt10	1,514	↑Zt09: 1062 ↑Zt10: 541
GO groups enriched^c^	**GO term**	**DE genes in GO group**	**GO ID**
	Transmembrane transport	159 (of 491)	GO:0055085
	Carbohydrate metabolic process	82 (of 242)	GO:0005975
	Proteolysis	64 (of 237)	GO:0006508
	Amino acid transmembrane transport	17 (of 36)	GO:0003333
	Oxidation–reduction process	159 (of 627)	GO:0055114
	Lipid catabolic process	6 (of 15)	GO:0016042
Candidate secreted effector genes	245 of 370 effector candidate genes		
	**Comparison (all four stages)**	**DE effector genes** d	**Regulation**
	Zt05‐Zt09	161	↑Zt05: 120 ↑Zt09: 50
	Zt05‐Zt10	167	↑Zt05: 141 ↑Zt10: 31
	Zt09‐Zt10	193	↑Zt09: 167 ↑Zt10: 60

Note

DE: differentially expressed. ↑: significantly up‐regulated in the isolate.

Summary of genes that are differentially expressed between the three *Z. tritici* isolates Zt05, Zt09, and Zt10 during the four infection stages.

a Differentially expressed genes identified by DESeq2, *p*_adj_ ≤0.01, |log_2_ fold change| ≥2.

b 774 genes are differentially expressed in at least two isolate comparisons.

c Gene Ontology (GO) group enrichment analyses by topGO for ontology “Biological Process,” *p* ≤ 0.01.

d 198 effector candidate genes are differentially expressed in at least two isolate comparisons.

**Figure 5:**
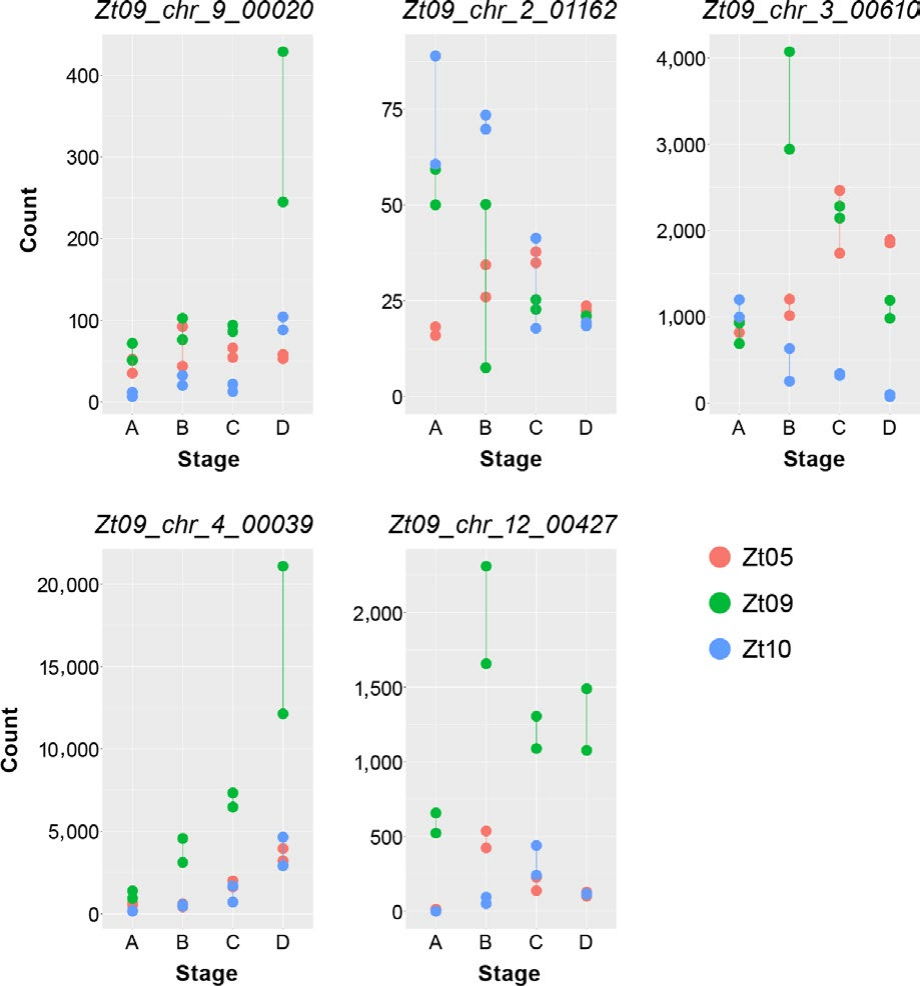
Five effector candidates with highly differentiated expression profiles in the three isolates during wheat infection. The plots display normalized read counts for five effector candidate genes for the twelve RNA‐seq datasets. Read counts were normalized across the four core infection stages (A to D) and the three *Z. tritici* isolates Zt05, Zt09, and Zt10 by applying the regularized log transformation (rlog) function of DESeq2 (Love et al., 2014) and represent a measure of relative gene expression between the infection stages and between the isolates

For all isolate comparisons, the identified differentially expressed genes are significantly enriched in CSEP genes (*p* ≤ 1.18 × 10^−7^, Fischer's exact tests) (Figure 4b) indicating isolate‐specific effector expression (Supporting Information Table S19). Figure 5 exemplifies the isolate‐specific expression kinetics of five CSEP genes. These five genes encode one hypothetical effector (*Zt09_chr_12_00427*) and secreted proteins with various functions: a hydrophobin (*Zt09_chr_9_00020*) also shown previously to be differentially expressed between Swiss field isolates (Palma‐Guerrero et al., 2017), a DNase (*Zt09_chr_2_01162*), and the ribonuclease Zt6 (*Zt09_chr_3_00610*), which possesses ribotoxin‐like activity and is cytotoxic against plants and various microbes (Kettles, Bayon, Sparks, et al., 2017). The gene *Zt09_chr_4_00039* encodes a protein with homology to the phytotoxin cerato‐platanin of *Ceratocystis fimbriata* which was shown to induce necrosis and defense responses in plane trees (Pazzagli et al., 1999). During all infection stages, *Zt09_chr_4_00039 *is significantly higher expressed in Zt09 and might contribute to the higher necrosis levels caused by Zt09 (Figure 1a).

In addition to the differences in the expression of CSEP genes, we also noted isolate‐specific expression patterns for genes located on accessory chromosomes. For example, three neighboring genes located on chromosome 19 in Zt09 (*Zt09_chr_19_00071* to *Zt09_chr_19_00073*) are significantly higher expressed in Zt10 during all four infection stages (Supporting Information Figure S15; Table S18), and other genes located ~70 kb downstream are likewise specifically up‐regulated in Zt10 compared to one or both of the other isolates. We consider the possibility that the genomic environment, including the presence of transposable elements, may influence the observed variation in transcription activity of an entire region. To address this hypothesis, we investigated the specific region in the genome alignment of the three isolates. In Zt10, these genes are located on unitig 16, which is syntenic to the accessory chromosome 19 of Zt09. Transposable elements (RLG elements) are located downstream of *Zt09_chr_19_00072* and upstream of *Zt09_chr_19_00073* in Zt09, but are not present within the vicinity of these genes on unitig 16 in Zt10. Instead, the right arm of unitig 16 of Zt10 (~100 Mb) is inverted compared to Zt09 and Zt05, and the inversion starts upstream of the gene *Zt09_chr_19_00073, *reflecting a significant change in sequence structure that might influence transcriptional regulation on this accessory chromosome.

### 3.9 Transposable elements are associated with the differentially expressed genes

Detailed analyses of the *Z. tritici* transcriptomes revealed considerable variation in the transcriptional landscapes among isolates. The extent of genetic differentiation between Zt05, Zt09, and Zt10 likely accounts for much of the transcriptional variation in the form of SNPs in regulatory sequences. However, other layers of gene regulation may also contribute to the heterogeneous transcriptional landscapes. We hypothesized that epigenetic transcriptional regulation, such as co‐regulation of sequences associated with transposable elements, could impact gene expression variation. In a previous study, we showed that transposable elements and the accessory chromosomes of *Z. tritici* are enriched with the histone modifications H3K9me3 and H3K27me3, which are associated with repressive regions of chromatin (Schotanus et al., 2015). In *Fusarium graminearum*, the histone modification H3K27me3 is associated with gene clusters encoding secondary metabolites and pathogenicity‐related traits (Connolly, Smith, & Freitag, 2013). It is possible that variation in the distribution of histone modifications like H3K27me3 across the genome sequences of Zt05, Zt09, and Zt10 contributes to the dramatic variation in expression phenotypes.

To test this, we assessed the distances of all genes to the closest annotated transposable element. In the genomes of all three isolates, we found that isolate‐specific differentially expressed genes are located significantly closer to transposable elements than genes that were not differentially expressed (Mann–Whitney U tests, *p* < 2.2 × 10^−16^). Although more than 50% of these differentially expressed genes are located downstream of the closest transposable element, we did not observe an overall enrichment of TEs in their putative promotor regions 2 kb upstream (Supporting Information Table S20). Among the differentially expressed genes, isolate‐specific up‐regulated genes (genes that are significantly up‐regulated in one isolate in contrast to the others) are significantly enriched within a distance of 2 kb to transposable elements in Zt05 and Zt09 (Fisher's exact tests, *p* ≤ 0.0094), but not in Zt10. We note, however, that the transposable element annotation in Zt10 is not as complete as in Zt05 and Zt09 as the annotation was based on the Illumina short read assembly which is 6.7 Mb smaller than the de novo genome assembly of SMRT Sequencing reads. We also analyzed the in vitro histone 3 methylation data for Zt09 (Schotanus et al., 2015) and found that differentially expressed genes are indeed significantly closer to H3K9me3 and H3K27me3 peaks (Mann–Whitney *U* tests, *p* < 2.2 × 10^−16^). Further, we observed significant enrichment of genes up‐regulated in Zt09 in comparison with Zt05 and Zt10 within a distance of 2 kb to H3K9me3 and H3K27me3 peaks (Fisher's exact tests, *p* ≤ 1.55 × 10^−15^), but down‐regulated genes were only enriched in the vicinity of H3K27me3 peaks (distance ≤2 kb, Fisher's exact test, *p* = 1.35 × 10^−15^). Poor transcription of genes located on the accessory chromosomes was explained by enrichment of H3K27me3 covering the entire chromosomes and H3K9me3, which is mostly associated with repetitive DNA (Schotanus et al., 2015). Our findings indicate that during host infection, chromatin state of repeat‐rich genome compartments is highly dynamic and changes between “active” euchromatin and “repressive” heterochromatin, as suggested in *Leptosphaeria maculans* (Soyer et al., 2014). Further, our observation suggests that the fine‐scale distribution of epigenetic marks likely differs between the genomes of *Z. tritici* isolates and contributes to the isolate‐specific gene expression phenotypes that we observed. To further visualize the transcriptional landscape across the three *Z. tritici* genomes, we calculated expression values (RPKM) of 1‐kb windows (Supporting Information Table S11) and plotted them in heatmaps along the chromosomes and unitigs (Supporting Information Figures S16–S18). This approach allows visualization of the transcriptional landscapes at a high resolution. Thereby, we identified heterogeneous gene expression patterns across chromosomes, such as on chromosome 19 in Zt10 (Supporting Information Figure S18) and demonstrated conservation of previously identified patterns, such as transcriptional silencing of the right arm of chromosome 7 (Kellner et al., 2014; Rudd et al., 2015) in Zt05, Zt09, and Zt10 (Supporting Information Figure S17). This chromosomal segment has characteristics of an accessory chromosome, as it is significantly enriched with H3K27me3 that mediates transcriptional silencing (Schotanus et al., 2015). While syntenic chromosomal regions generally have a similar composition of transcribed and silenced loci, the fine‐scale distribution of transcriptional cold and hot spots is clearly different between the genomes of the three isolates studied here.

## 4 DISCUSSION

Many plant pathogens have a narrow host range and are specialized to colonize and reproduce in a small number of host plant species or host genotypes (Haueisen & Stukenbrock, 2016; van der Does & Rep, 2017). By combining advanced microscopy and comparative transcriptome analyses, we addressed the consequences of host specialization in the specialized wheat pathogen *Z. tritici*. We specifically asked whether one particular virulence phenotype results from one specific infection program. Our infection experiments and transcriptome data analyses reveal an unexpectedly high extent of plasticity in the infection program. The three studied *Z. tritici* isolates differ significantly in their genomic composition, and we show that the genetic variation and isolate‐specific transcriptional reprogramming during the different stages of host infection translate into highly distinct phenotypes that deviate temporally, spatially, and morphologically. Gene expression profiles associated with host colonization show a high degree of variability: More than 20% of the core genes are differentially expressed between these three *Z. tritici* isolates during the four infection stages indicating different feeding and molecular host interaction strategies. Candidates for effectors and putatively phytotoxic and antimicrobial molecules are enriched among the differentially expressed genes, suggesting that the three isolates employ different molecular strategies to interact with wheat and its associated microbiome. Effectors with cytotoxic activity have been proposed to shape the host environment by manipulating host and the associated microbiome in favor of the pathogen (Snelders, Kettles, Rudd, & Thomma, 2018). The three isolates show different abilities to grow epi‐ and endophytically during the symptomless stages of infection and consequently are differentially exposed to biotic and abiotic influences, such as host responses, microbial competitors, or pathogen control treatments. Strikingly, the highly variable infection programs result in the same level of virulence, showing that “host specialization” in *Z. tritici* involves a very flexible strategy to exploit wheat tissue for growth and reproduction. This flexibility may be facilitated by the fact that pathogenesis does not involve the formation of complex penetration or feeding structures that require fine‐tuned developmental programs. In powdery mildew which need to form appressoria as well as haustoria to establish host infections, comparative transcriptomics during early pathogenesis identified a comparatively low number of isolate‐specifically expressed genes in two barley powdery mildew strains (Hacquard et al., 2013) and showed that transcriptional programs are similar even during incompatible infections and interactions with nonhost species (Hacquard et al., 2013; Hu et al., 2018).

As necrotic lesions are usually composed of several distinct *Z. tritici* genotypes (Linde et al., 2002), it would be highly relevant to investigate whether strains in one lesion have similar or different infection phenotypes. Isolates with different infection strategies colonizing the same leaf could complement each other or have antagonistic effects. Future multi‐isolate studies must however consider possible developmental asynchrony during infection and should focus on isolates showing similar temporal infection development. Our findings likewise underline the importance to consider not only quantitative infection outcomes but also qualitative aspects, like the timing of disease progress over an appropriate period of time, to be able to compare the infections of different pathogen isolates and detect meaningful differences in their infection phenotypes (Habig et al., 2017; Meile et al., 2018).

An intriguing question that emerges from our analyses is which factors cause deviation in gene expression phenotypes in *Z. tritici*. Genetic variants associated with transcriptional regulation likely contribute to differences in gene regulation. However, we hypothesize that variation in epigenetic traits promotes different transcriptional programs. Genome‐wide patterns of transcriptional activity (Supporting Information Figures S16–S18) indeed suggest some variation in the physical distribution of transcriptionally active and silent regions, which may result from distinct epigenetic landscapes related to histone modifications or DNA methylation.

The capacity of *Z. tritici* populations to harbor and maintain a broad diversity of infection programs might facilitate flexibility and dynamic responses to changes in their host environment, for example, fungicide treatments during the season or the introduction of new host genotypes. Furthermore, the significant variation in expression of virulence factors such as effectors, secondary metabolites, and plant cell wall‐degrading enzymes suggests that different individuals of *Z. tritici* employ different molecular strategies to interact with the same host environment. We hypothesize that highly diverging infection phenotypes are not exclusive among isolates of *Z. tritici *and are likely found in populations of other pathogens that retain high levels of genetic diversity. Variation in infection and expression profiles contributes another layer of polymorphism in pathogen populations that has so far been neglected in most systems. Many studies identifying virulence factors focus on the molecular interactions between reference genotypes—one pathogen isolate and one host genotype. Our work challenges the relevance of these specific interactions in the field on population scales and emphasizes the importance to consider the diversity of pathogen infection programs to improve our understanding of pathogen evolution and the ecology of plant pathogens.

## CONFLICT OF INTEREST

None declared.

## AUTHOR CONTRIBUTION

Conceptualization: JH and EHS. Wheat infection experiments and in vitro stress assay: JH. *In planta *ROS assay: JH and HS. Karyotyping and synteny analyses: JH and MM. Confocal microscopy analyses and data visualization: JH and HA. Genome sequencing and de novo genome assemblies: MM and CJH. Transcriptome sequencing, data curation, and comparative transcriptome analyses: JH. Gene and TE annotations, software for enrichment analyses: JG. Preparation and writing of manuscript: JH and EHS. Editing of original manuscript: JH, EHS, MM, and HS.

## DATA ACCESSIBILITY

All generated RNA‐seq datasets have been deposited at the NCBI Gene Expression Omnibus and are accessible with the accession number GSE106136. De novo genome assemblies of isolates Zt05 and Zt10 are available under accession numbers PEBP00000000 and PEBO00000000. The genome sequence of the reference isolate IPO323 used for transcriptome analysis of Zt09 is available at: http://genome.jgi.doe.gov/Mycgr3/Mycgr3.home.html. Genome assemblies based on whole‐genome shotgun sequencing (Illumina) were also used; the assembly for Zt10 is available at GenBank (Zt10 = STIR04_A26b) GCA_000223645.2. Sequencing data and assembly for Zt05 (Zt05 = MgDk09_U34) are available through NCBI BioProject PRJNA312067 (Grandaubert et al., 2017).
