# Supplementary material for: Highly flexible infection programs in a specialized wheat pathogen
Source: Ecol Evol. 2018 Dec 26;9(1):275–94. doi: 10.1002/ece3.4724 (PMC6342133; doi:10.1002/ece3.4724)
Supplement: Supplementary file 1 [file ECE3-9-275-s001.pdf]

Figure S1

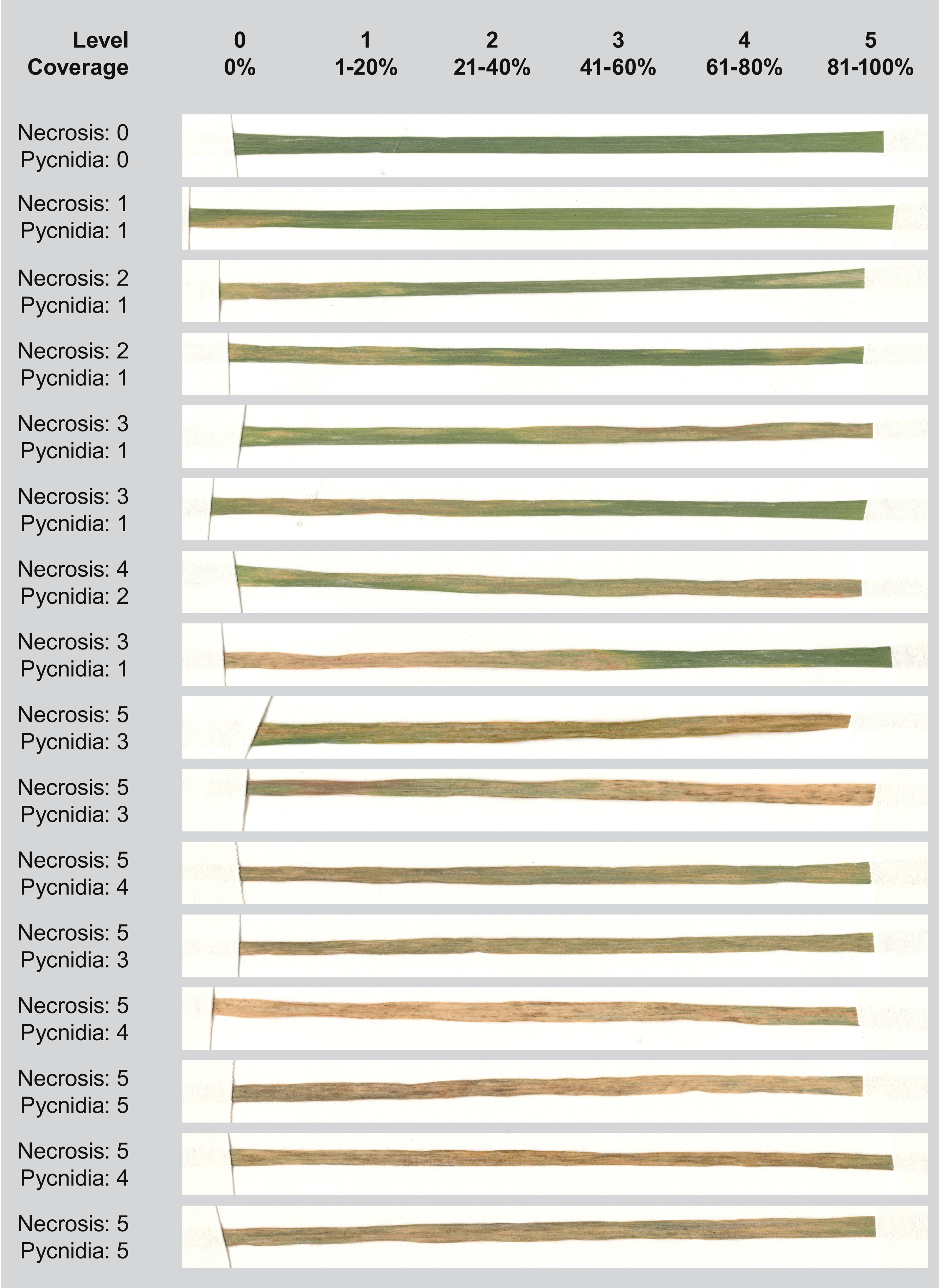
Level
Coverage
0
0%
1
1-20%
2
21-40%
3
41-60%
4
61-80%
5
81-100%
Necrosis: 0
Pycnidia: 0
Necrosis: 1
Pycnidia: 1
Necrosis: 2
Pycnidia: 1
Necrosis: 2
Pycnidia: 1
Necrosis: 3
Pycnidia: 1
Necrosis: 3
Pycnidia: 1
Necrosis: 4
Pycnidia: 2
Necrosis: 3
Pycnidia: 1
Necrosis: 5
Pycnidia: 3
Necrosis: 5
Pycnidia: 3
Necrosis: 5
Pycnidia: 4
Necrosis: 5
Pycnidia: 3
Necrosis: 5
Pycnidia: 4
Necrosis: 5
Pycnidia: 5
Necrosis: 5
Pycnidia: 4
Necrosis: 5
Pycnidia: 5

Figure S2

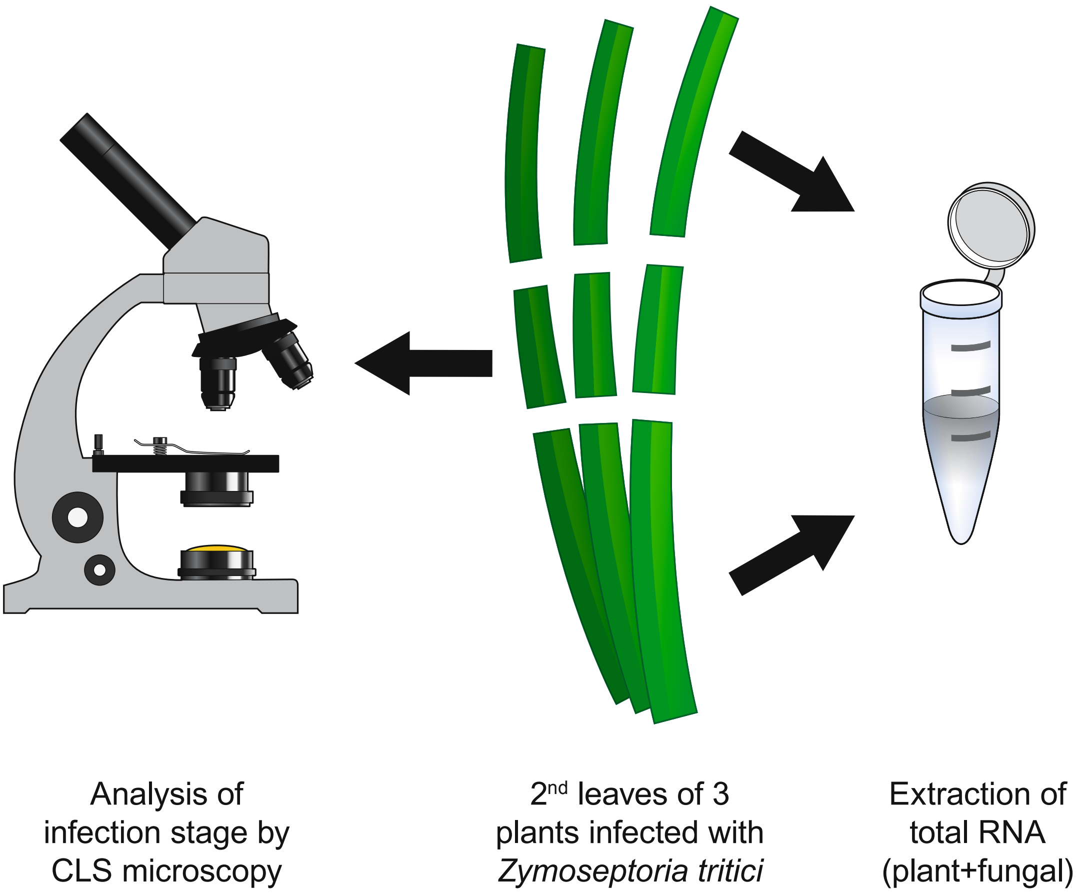
Analysis of
infection stage by
CLS microscopy
2nd leaves of 3
plants infected with
Zymoseptoria tritici
Extraction of
total RNA
(plant+fungal)

Figure S3

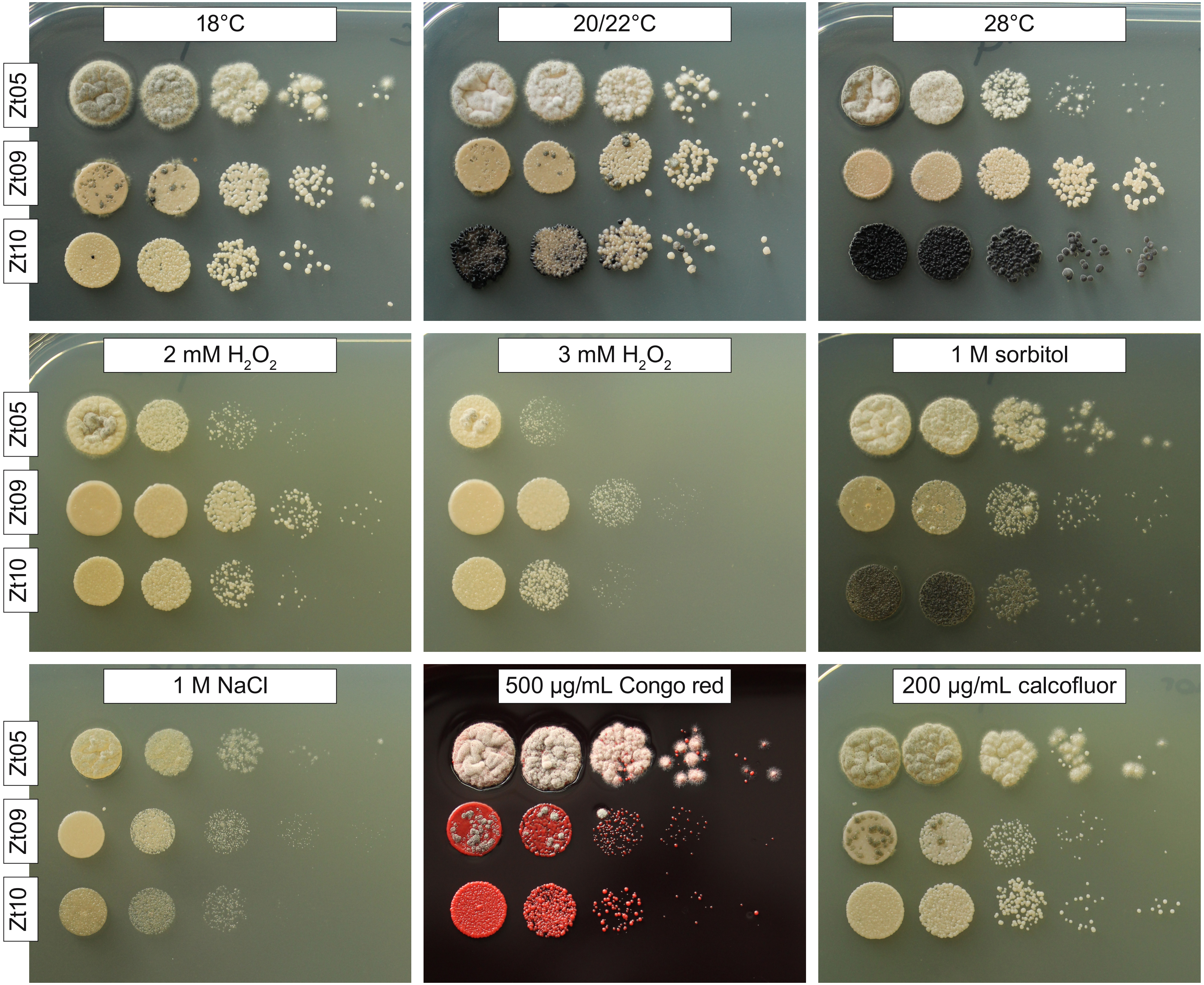
18°C
20/22°C
28°C
2 mM H_2O_2
3 mM H_2O_2
1 M sorbitol
1 M NaCl
500 µg/mL Congo red
200 µg/mL calcofluor
Zt05
Zt09
Zt10

Figure S4

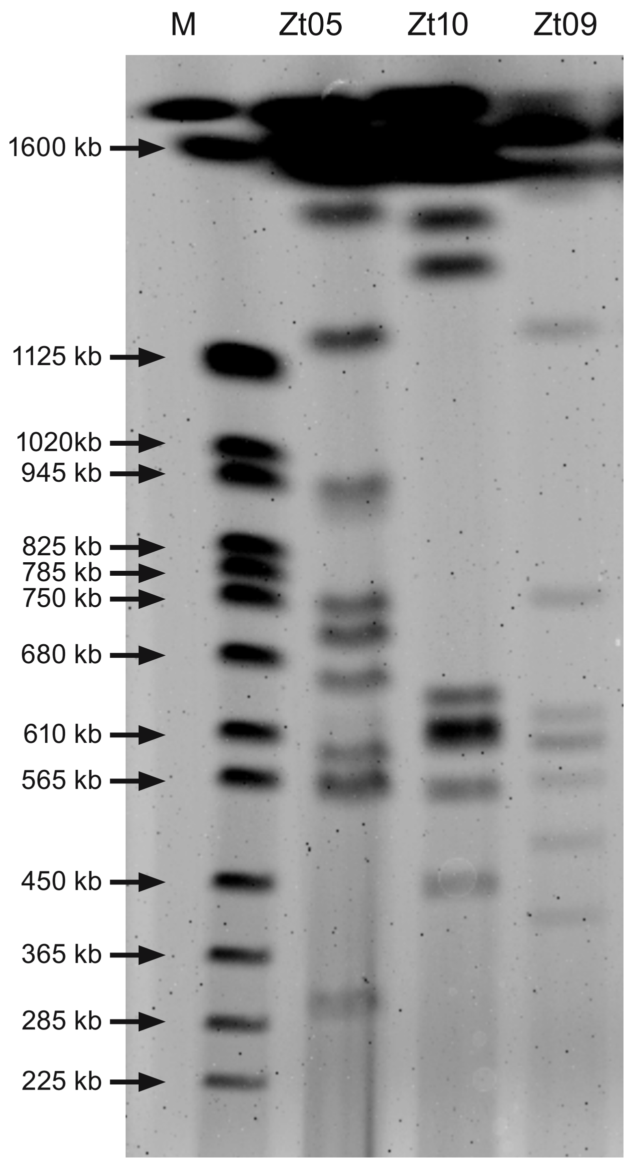
M
Zt05
Zt10
Zt09
1600 kb
1125 kb
1020kb
945 kb
825 kb
785 kb
750 kb
680 kb
610 kb
565 kb
450 kb
365 kb
285 kb
225 kb

Figure S5

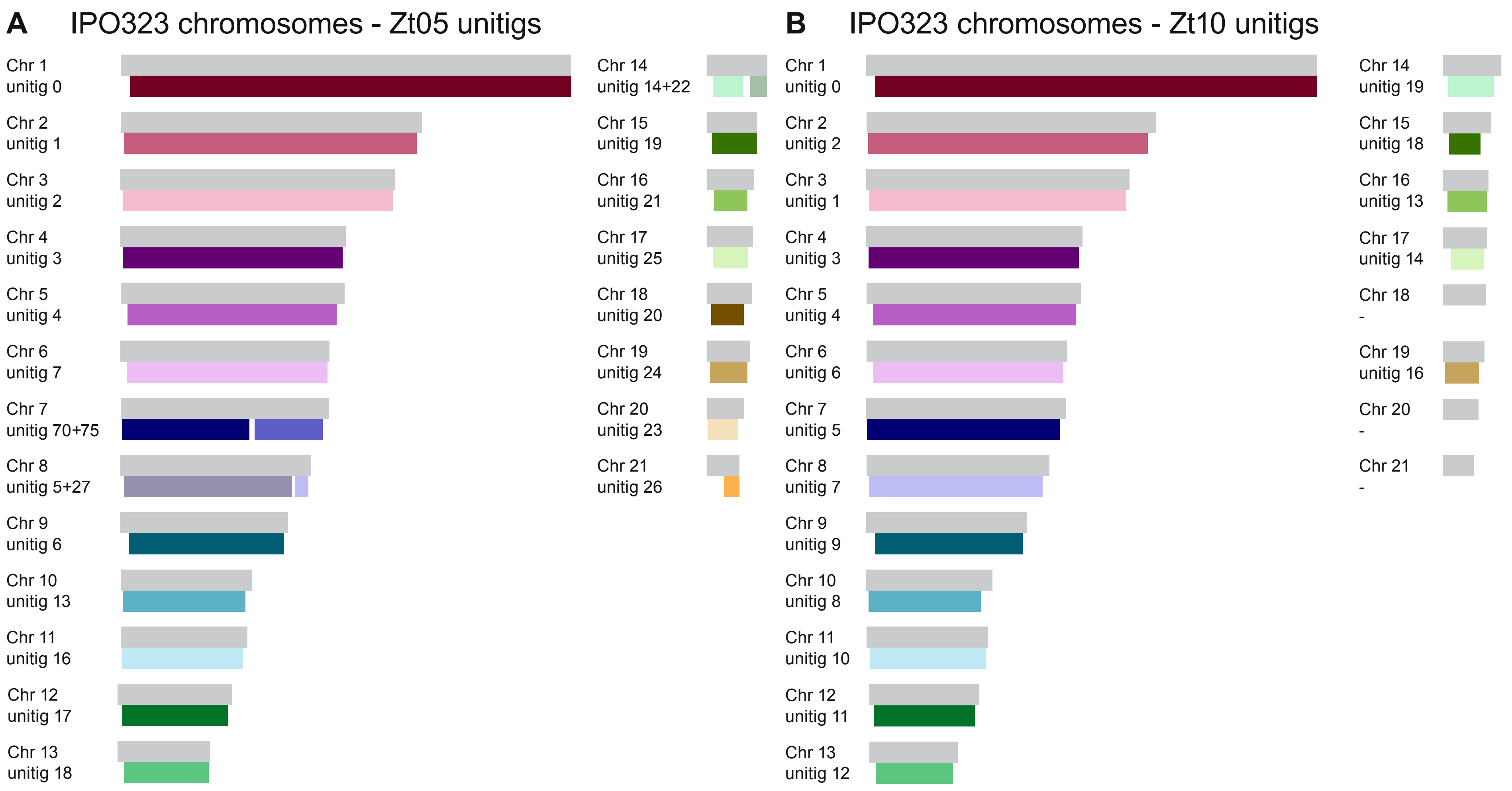
A
IPO323 chromosomes - Zt05 unitigs
Chr 1
unitig 0
Chr 2
unitig 1
Chr 3
unitig 2
Chr 4
unitig 3
Chr 5
unitig 4
Chr 6
unitig 7
Chr 7
unitig 70+75
Chr 8
unitig 5+27
Chr 9
unitig 6
Chr 10
unitig 13
Chr 11
unitig 16
Chr 12
unitig 17
Chr 13
unitig 18
Chr 14
unitig 14+22
Chr 15
unitig 19
Chr 16
unitig 21
Chr 17
unitig 25
Chr 18
unitig 20
Chr 19
unitig 24
Chr 20
unitig 23
Chr 21
unitig 26
B
IPO323 chromosomes - Zt10 unitigs
Chr 1
unitig 0
Chr 2
unitig 2
Chr 3
unitig 1
Chr 4
unitig 3
Chr 5
unitig 4
Chr 6
unitig 6
Chr 7
unitig 5
Chr 8
unitig 7
Chr 9
unitig 9
Chr 10
unitig 8
Chr 11
unitig 10
Chr 12
unitig 11
Chr 13
unitig 12
Chr 14
unitig 19
Chr 15
unitig 18
Chr 16
unitig 13
Chr 17
unitig 14
Chr 18
-
Chr 19
unitig 16
Chr 20
-
Chr 21
-

Figure S6

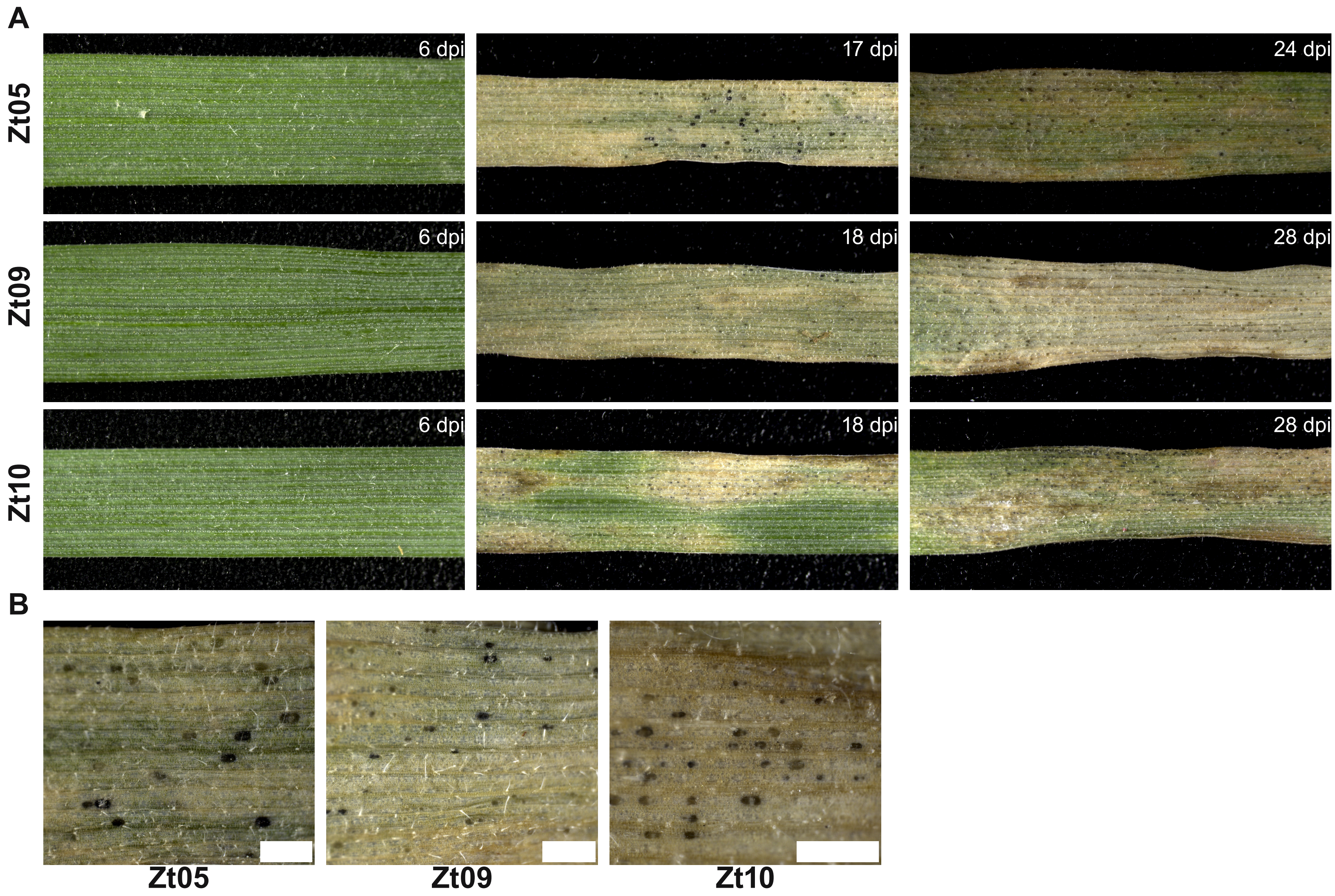
A
Zt05
Zt09
Zt10
6 dpi
17 dpi
24 dpi
6 dpi
18 dpi
28 dpi
6 dpi
18 dpi
28 dpi
B
Zt05
Zt09
Zt10

Figure S7

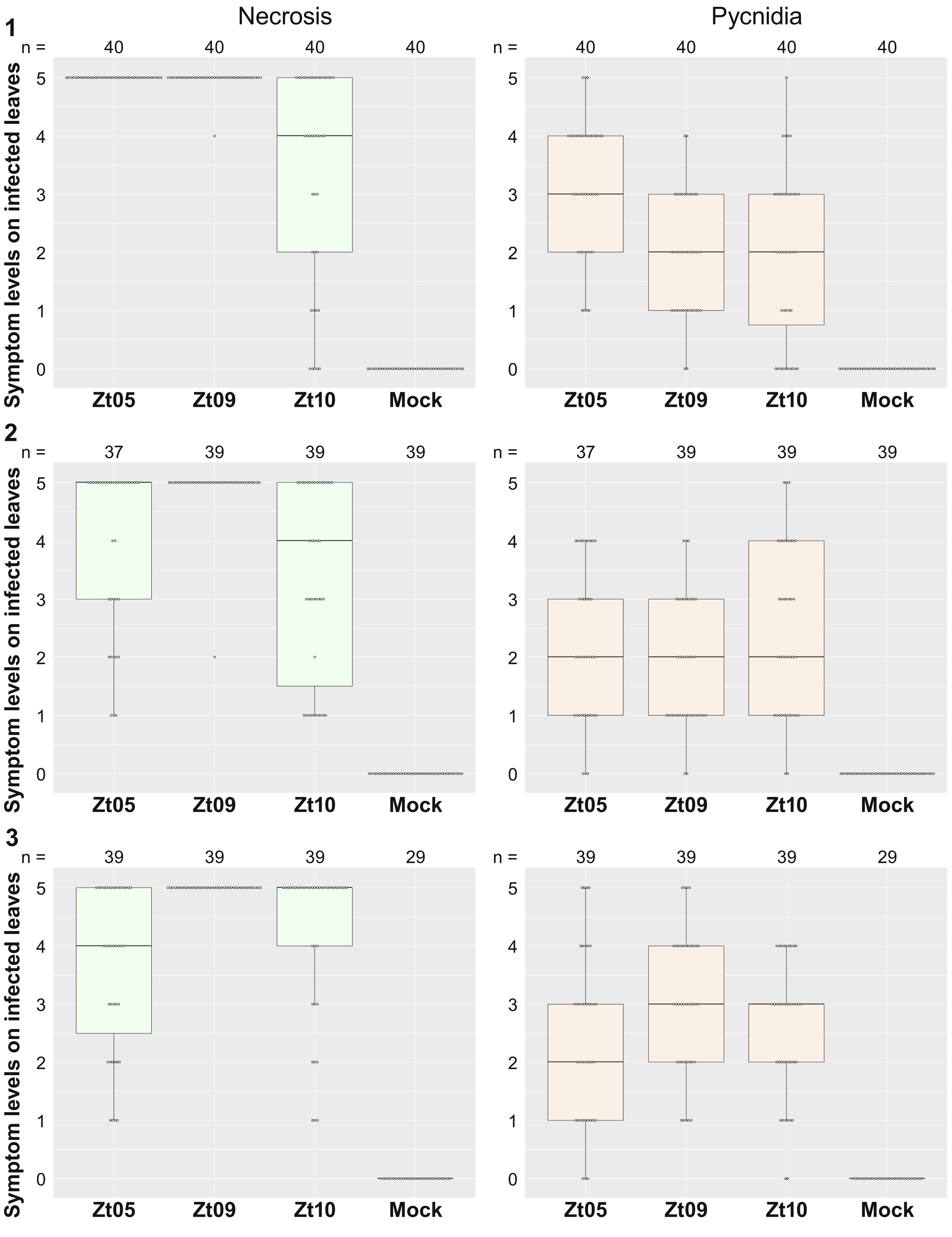

Necrosis
Pycnidia
1
2
3
n =
40
40
40
40
37
39
39
39
29
Symptom levels on infected leaves
0
1
2
3
4
5
Zt05
Zt09
Zt10
Mock

Figure S8

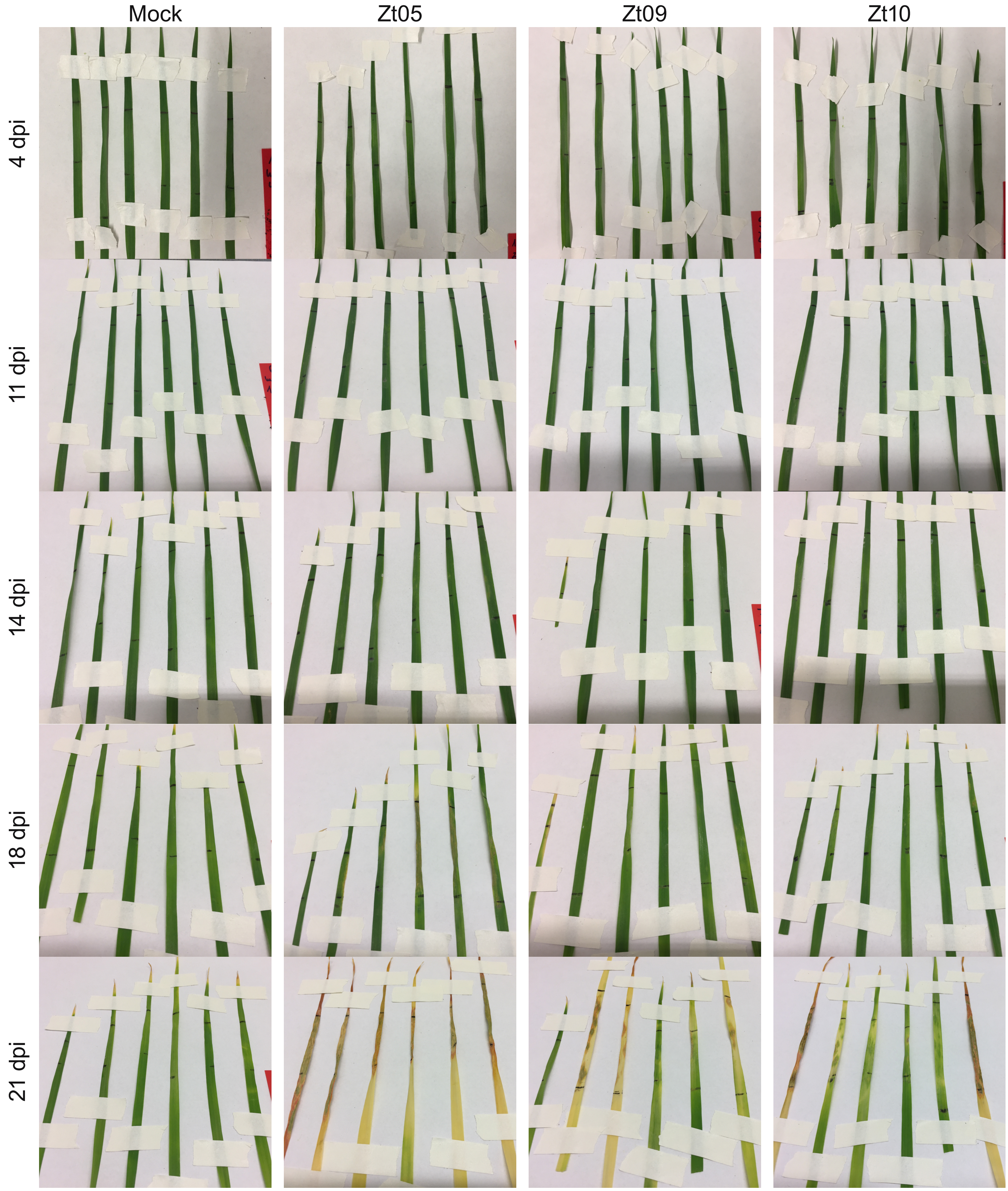

Figure S9

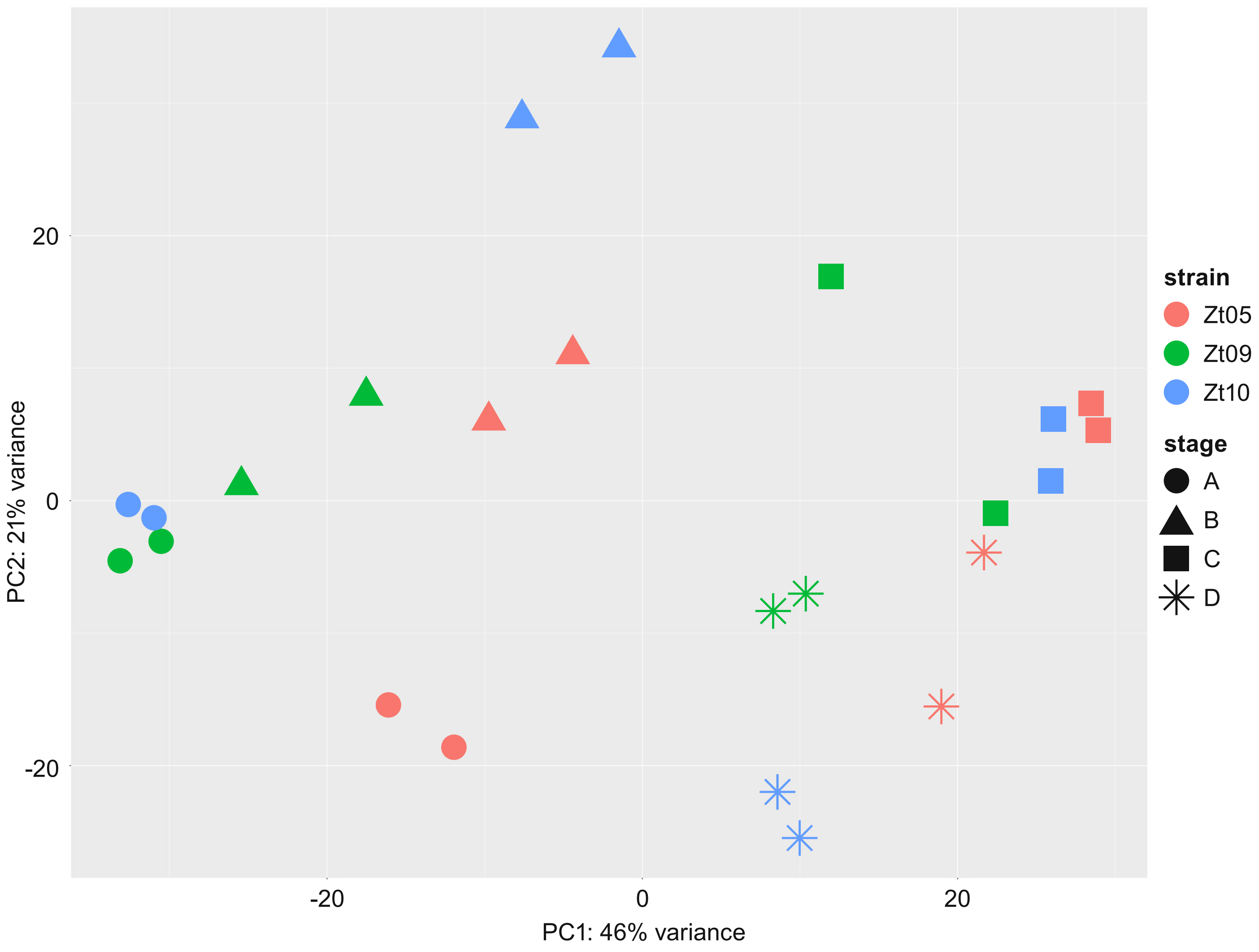

20
0
-20
-20
0
20
PC1: 46% variance
PC2: 21% variance
strain
Zt05
Zt09
Zt10
stage
A
B
C
D

Figure S10

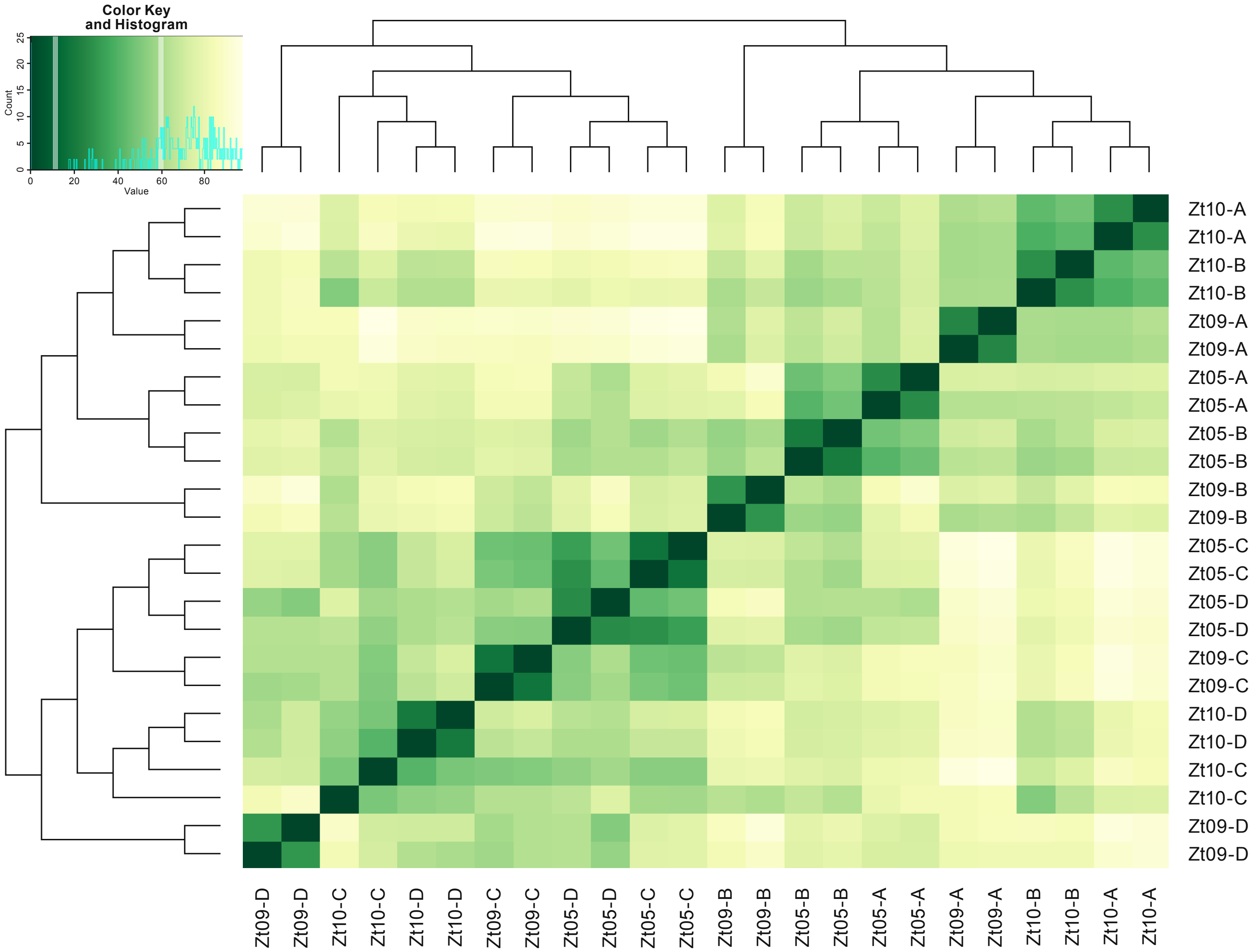
Color Key
and Histogram
Count
Value
0
20
40
60
80
0
5
10
15
20
25
Zt10-A
Zt10-A
Zt10-B
Zt10-B
Zt09-A
Zt09-A
Zt05-A
Zt05-A
Zt05-B
Zt05-B
Zt09-B
Zt09-B
Zt05-C
Zt05-C
Zt05-D
Zt05-D
Zt09-C
Zt09-C
Zt10-D
Zt10-D
Zt10-C
Zt10-C
Zt09-D
Zt09-D

Figure S11

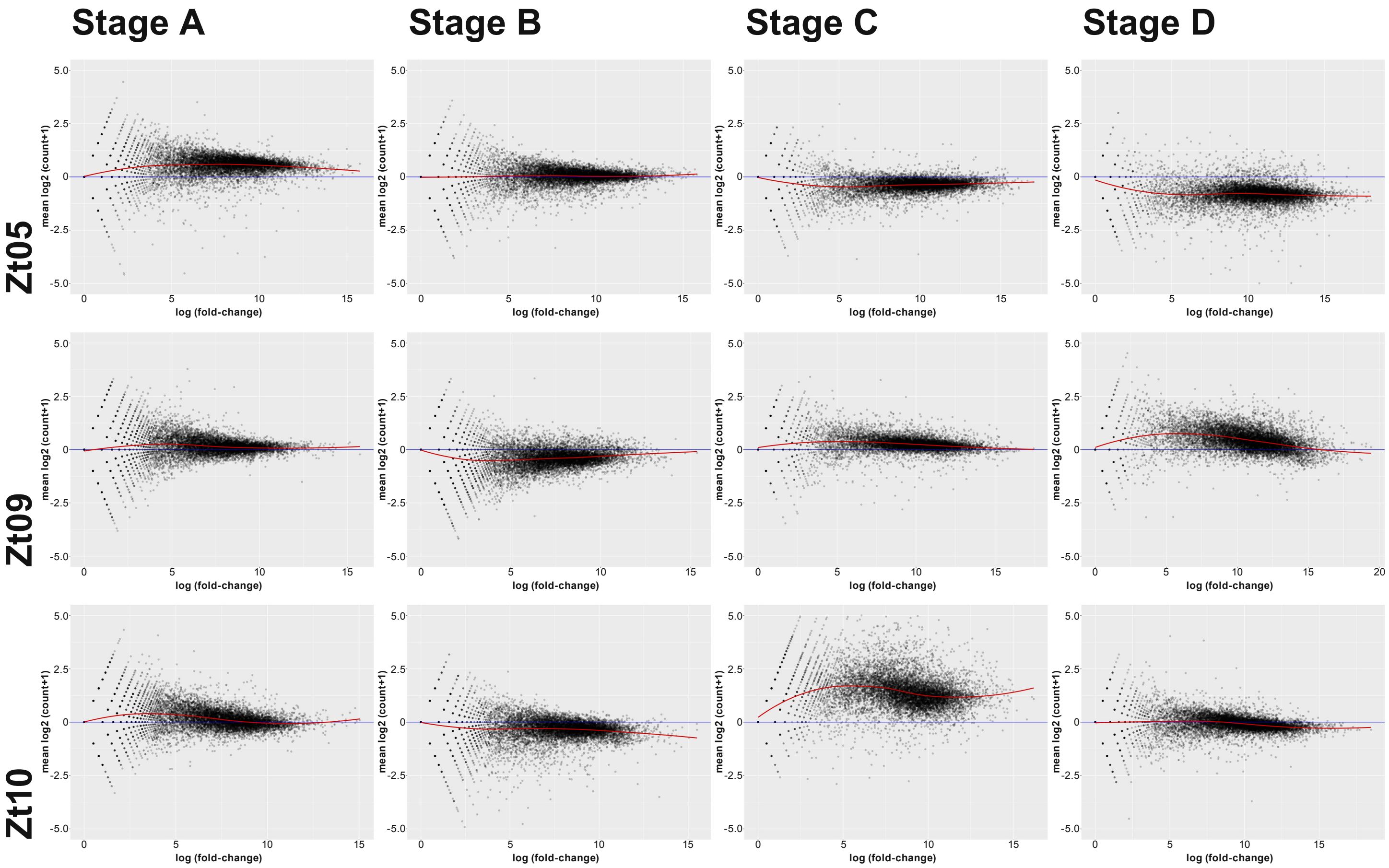
Stage A
Stage B
Stage C
Stage D
Zt05
Zt09
Zt10
mean log2 (count+1)
log (fold-change)

Figure S12

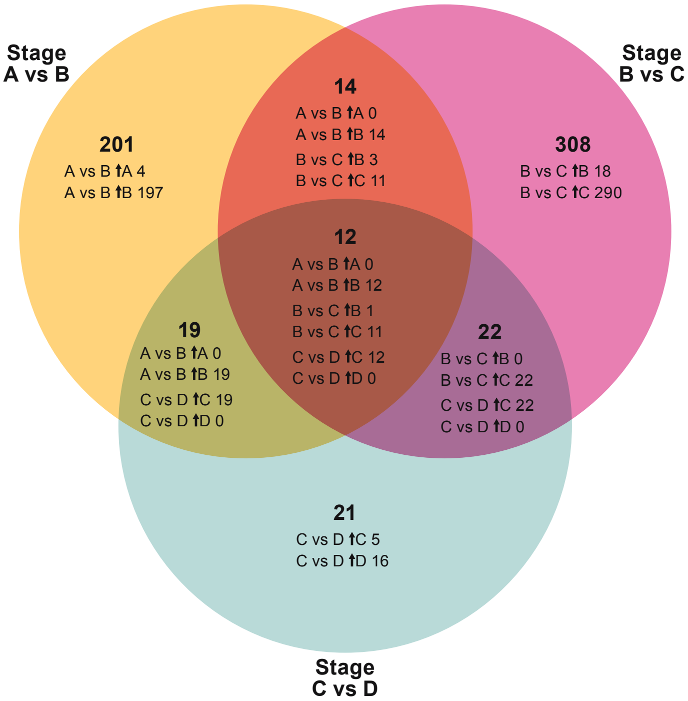

Stage
A vs B
Stage
B vs C
Stage
C vs D
201
A vs B ↑A 4
A vs B ↑B 197
14
A vs B ↑A 0
A vs B ↑B 14
B vs C ↑B 3
B vs C ↑C 11
308
B vs C ↑B 18
B vs C ↑C 290
12
A vs B ↑A 0
A vs B ↑B 12
B vs C ↑B 1
B vs C ↑C 11
C vs D ↑C 12
C vs D ↑D 0
19
A vs B ↑A 0
A vs B ↑B 19
C vs D ↑C 19
C vs D ↑D 0
22
B vs C ↑B 0
B vs C ↑C 22
C vs D ↑C 22
C vs D ↑D 0
21
C vs D ↑C 5
C vs D ↑D 16

Figure S13_1

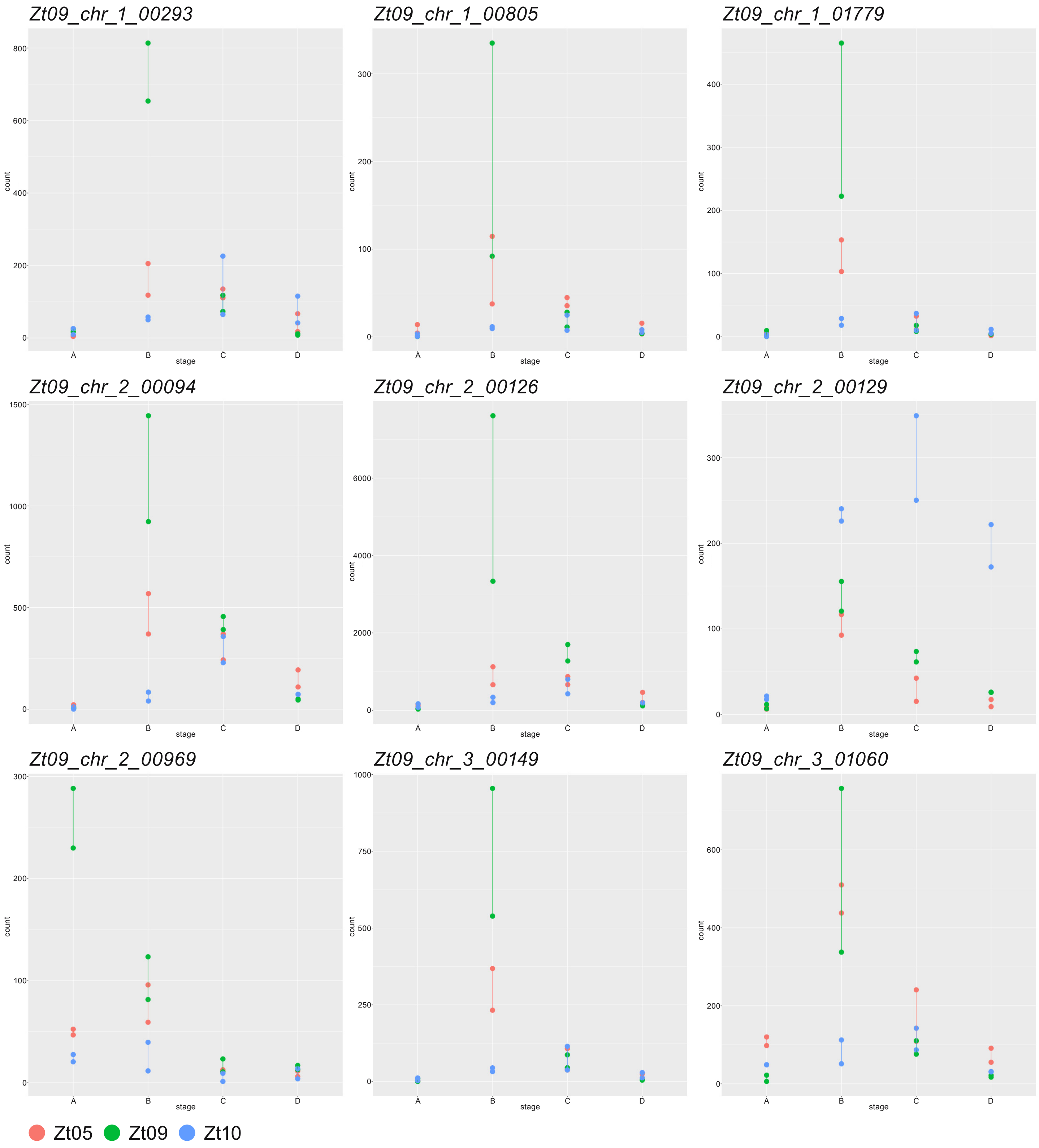
Zt09_chr_1_00293
Zt09_chr_1_00805
Zt09_chr_1_01779
Zt09_chr_2_00094
Zt09_chr_2_00126
Zt09_chr_2_00129
Zt09_chr_2_00969
Zt09_chr_3_00149
Zt09_chr_3_01060
count
stage
A
B
C
D
Zt05
Zt09
Zt10

Figure S13_2

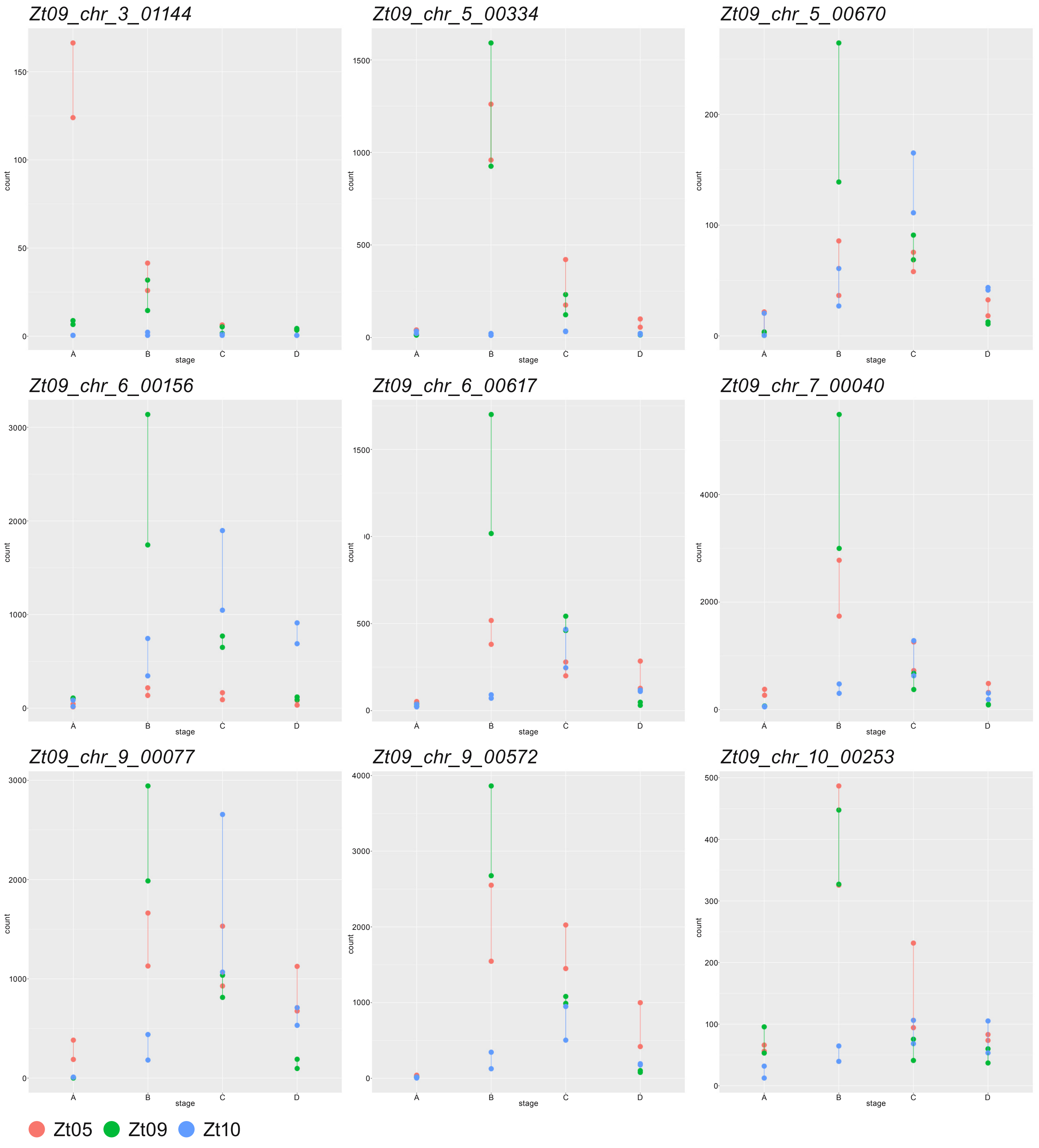

Zt09_chr_3_01144
Zt09_chr_5_00334
Zt09_chr_5_00670
Zt09_chr_6_00156
Zt09_chr_6_00617
Zt09_chr_7_00040
Zt09_chr_9_00077
Zt09_chr_9_00572
Zt09_chr_10_00253
count
stage
A
B
C
D
Zt05
Zt09
Zt10

Figure S13_3

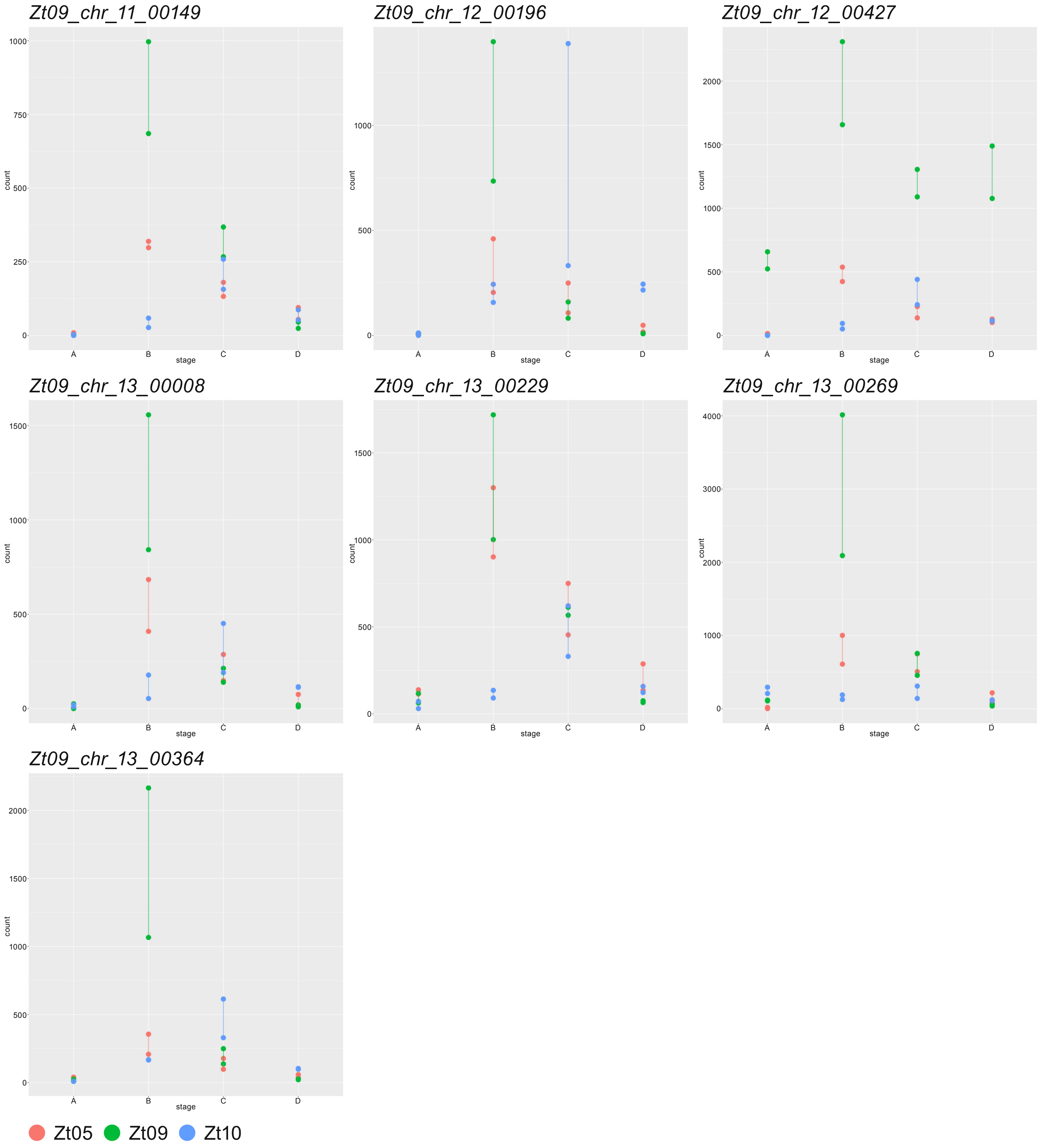
Zt09_chr_11_00149
Zt09_chr_12_00196
Zt09_chr_12_00427
Zt09_chr_13_00008
Zt09_chr_13_00229
Zt09_chr_13_00269
Zt09_chr_13_00364
count
stage
A
B
C
D
Zt05
Zt09
Zt10

Figure S14_1

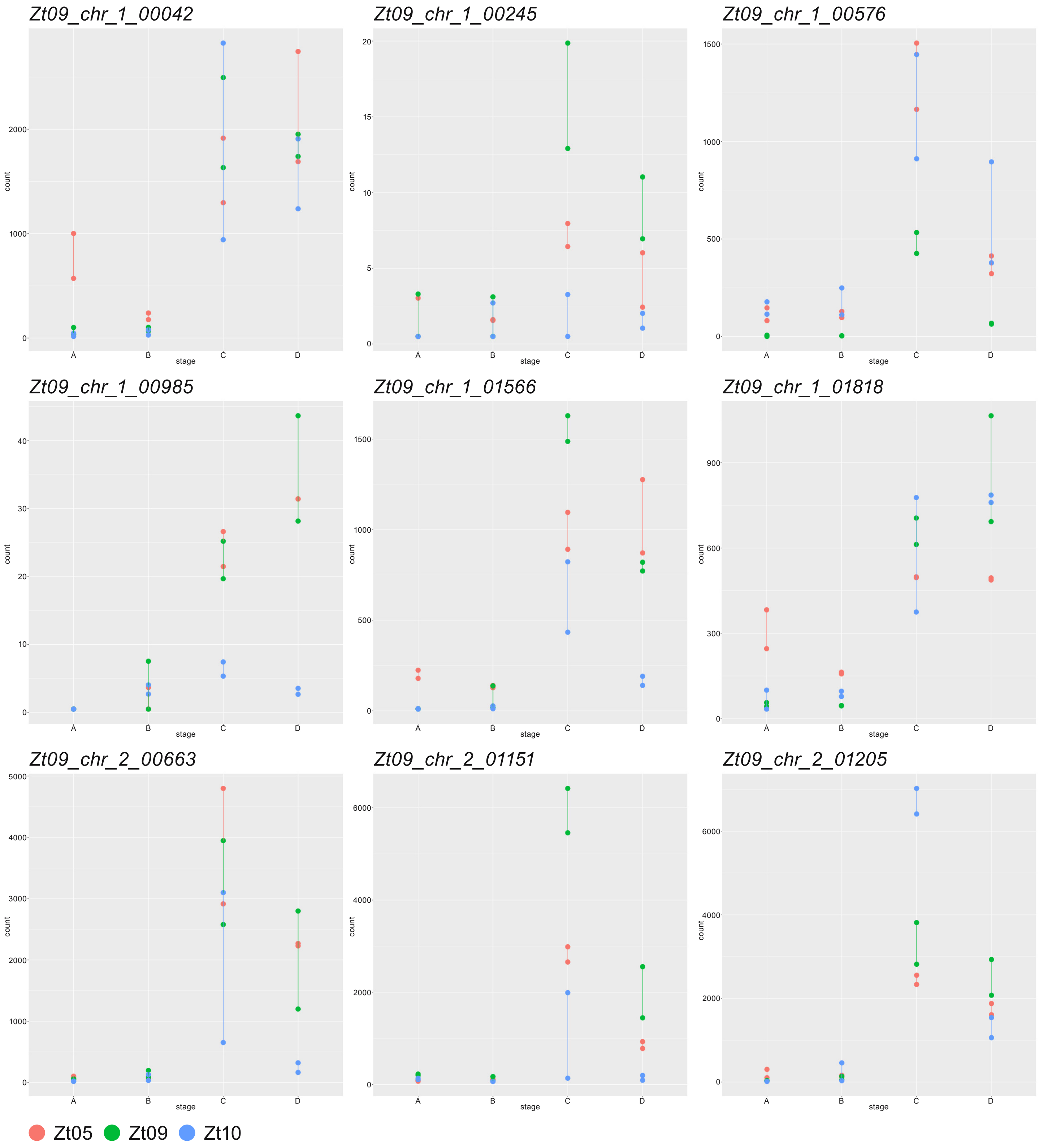

Zt09_chr_1_00042
Zt09_chr_1_00245
Zt09_chr_1_00576
Zt09_chr_1_00985
Zt09_chr_1_01566
Zt09_chr_1_01818
Zt09_chr_2_00663
Zt09_chr_2_01151
Zt09_chr_2_01205
count
stage
A
B
C
D
Zt05
Zt09
Zt10

Figure S14_2

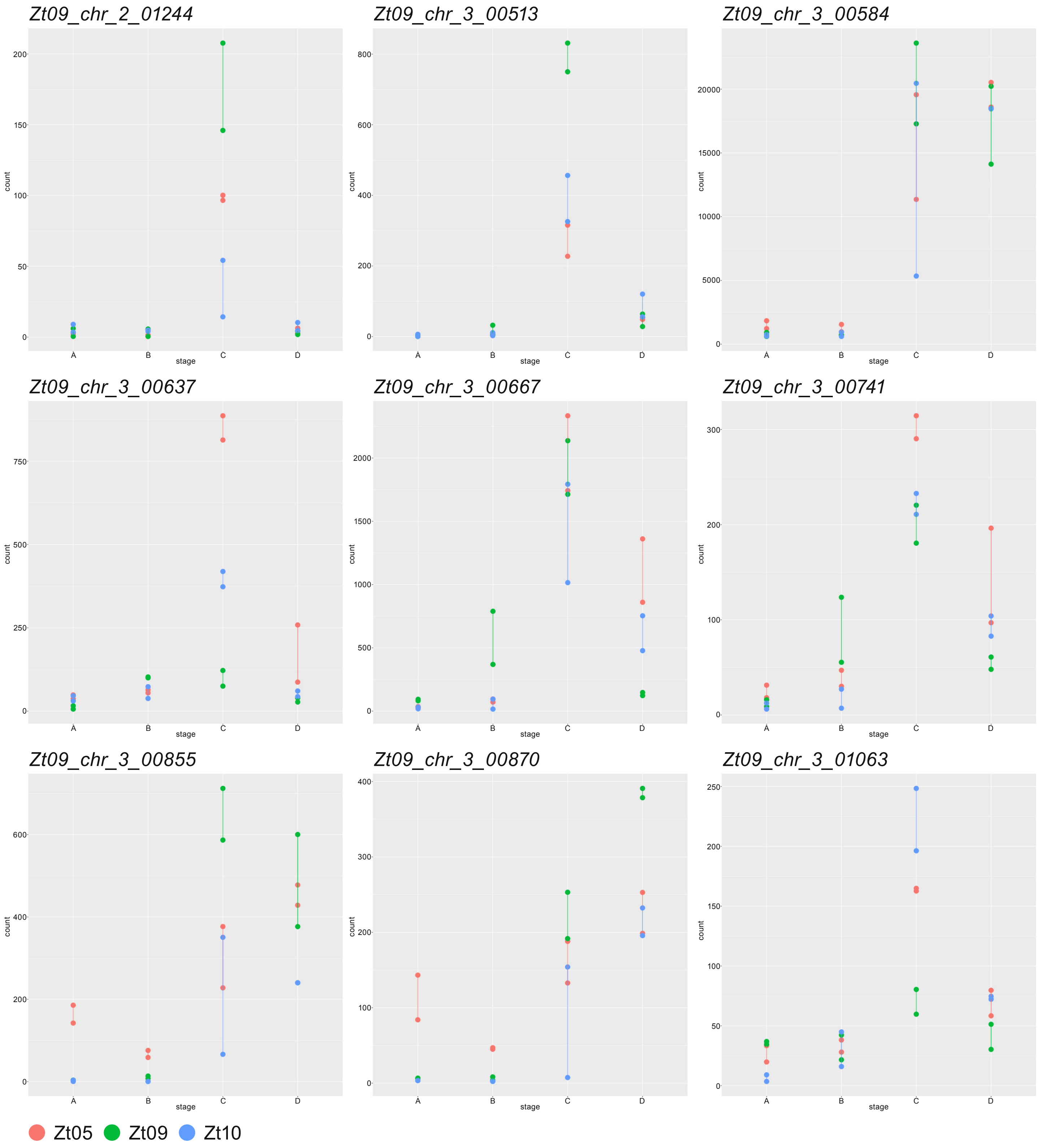

Zt09_chr_2_01244
Zt09_chr_3_00513
Zt09_chr_3_00584
Zt09_chr_3_00637
Zt09_chr_3_00667
Zt09_chr_3_00741
Zt09_chr_3_00855
Zt09_chr_3_00870
Zt09_chr_3_01063
count
stage
A
B
C
D
Zt05
Zt09
Zt10

Figure S14_3

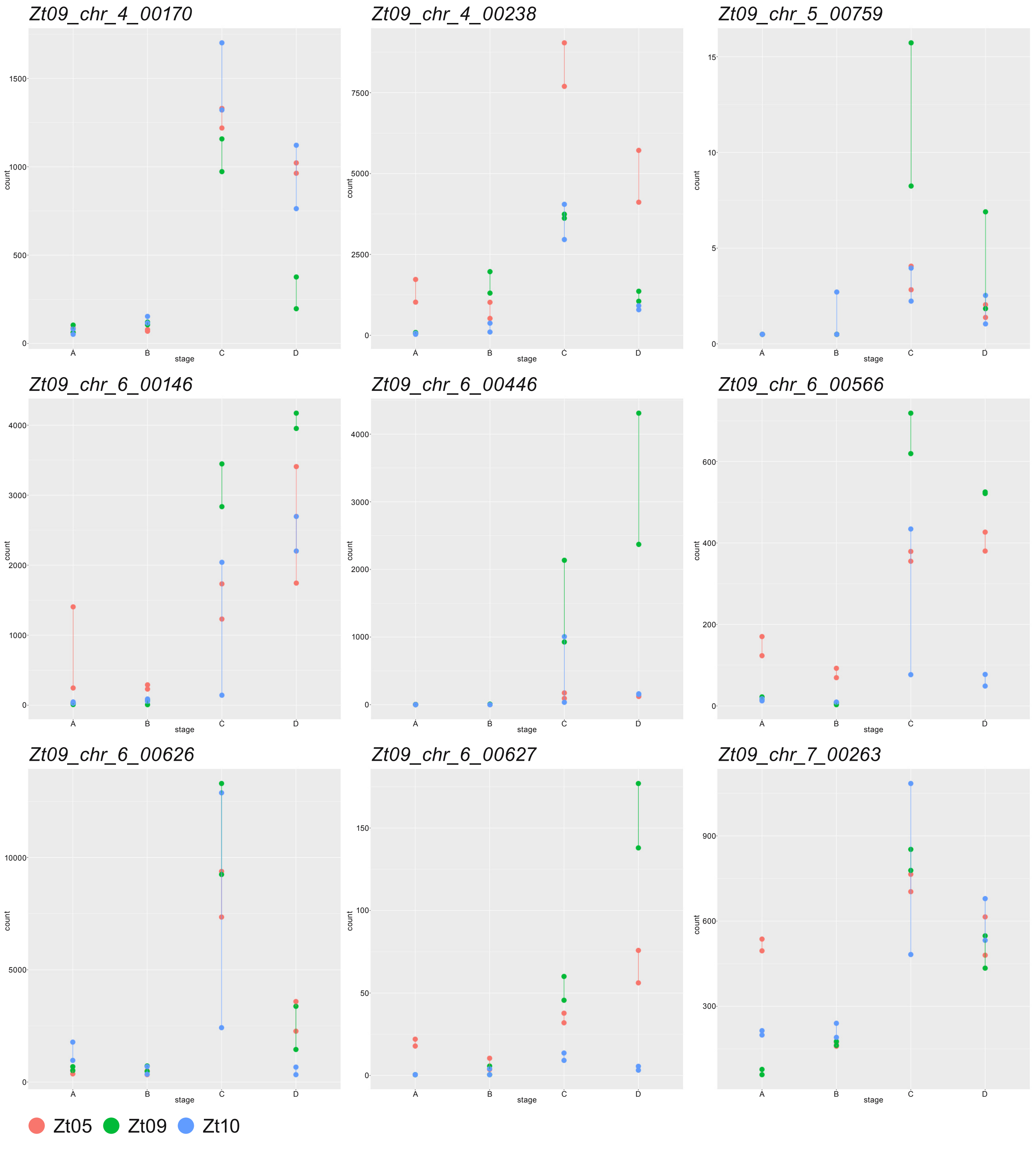

Zt09_chr_4_00170
Zt09_chr_4_00238
Zt09_chr_5_00759
Zt09_chr_6_00146
Zt09_chr_6_00446
Zt09_chr_6_00566
Zt09_chr_6_00626
Zt09_chr_6_00627
Zt09_chr_7_00263
count
stage
A
B
C
D
Zt05
Zt09
Zt10

Figure S14_4

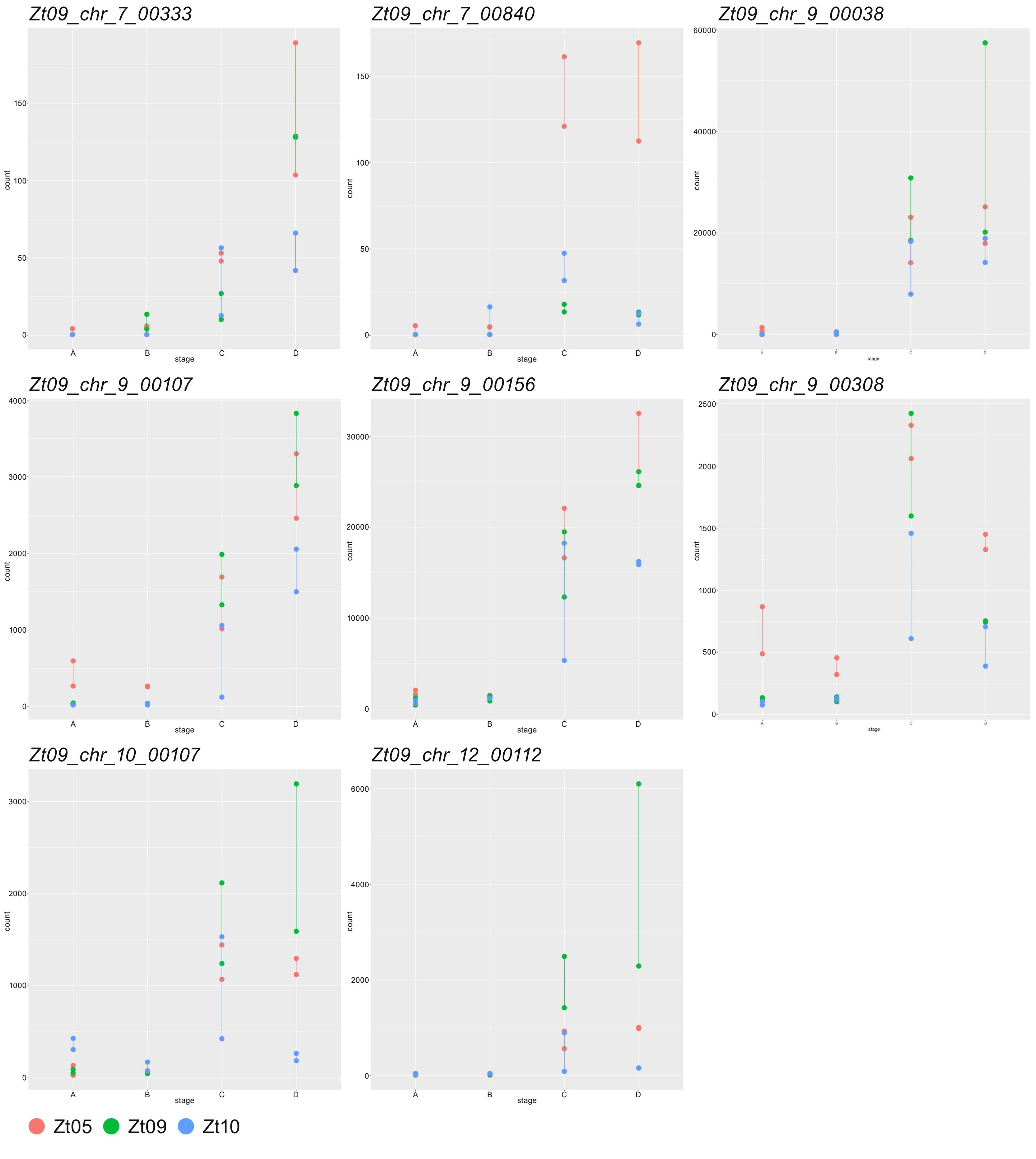
Zt09_chr_7_00333
Zt09_chr_7_00840
Zt09_chr_9_00038
Zt09_chr_9_00107
Zt09_chr_9_00156
Zt09_chr_9_00308
Zt09_chr_10_00107
Zt09_chr_12_00112
count
stage
A
B
C
D
Zt05
Zt09
Zt10

Figure S15

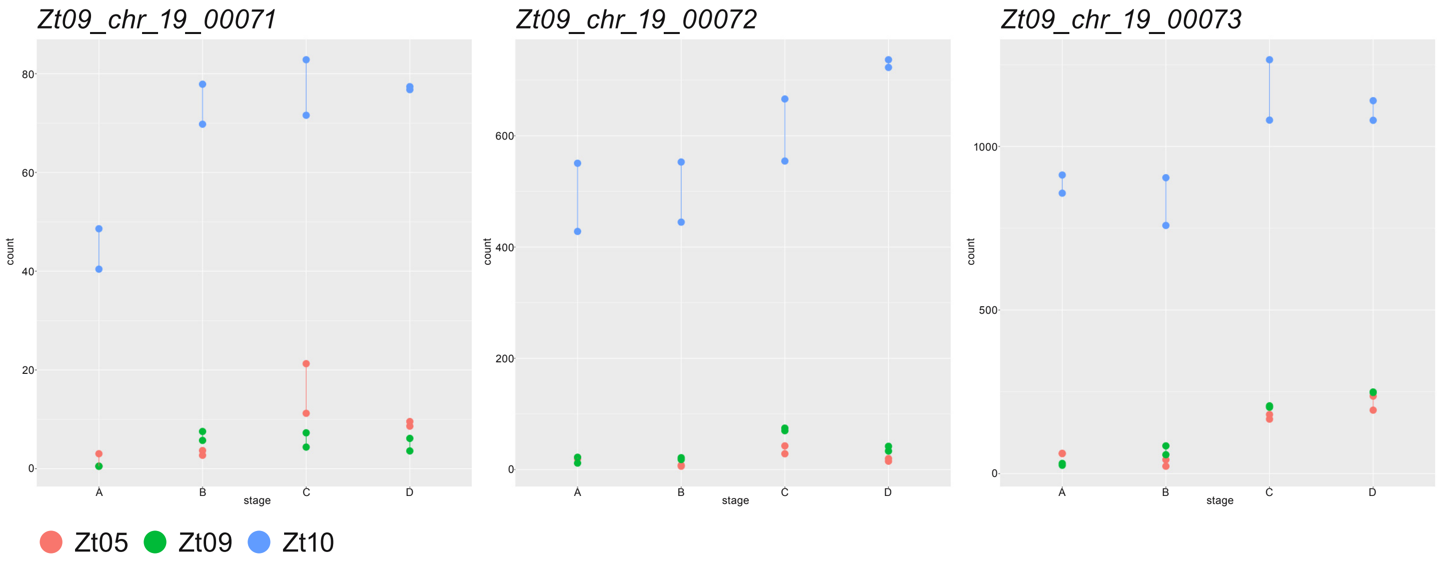

Zt09_chr_19_00071
Zt09_chr_19_00072
Zt09_chr_19_00073
count
stage
A
B
C
D
0
20
40
60
80
200
400
600
500
1000
Zt05
Zt09
Zt10

Figure S16

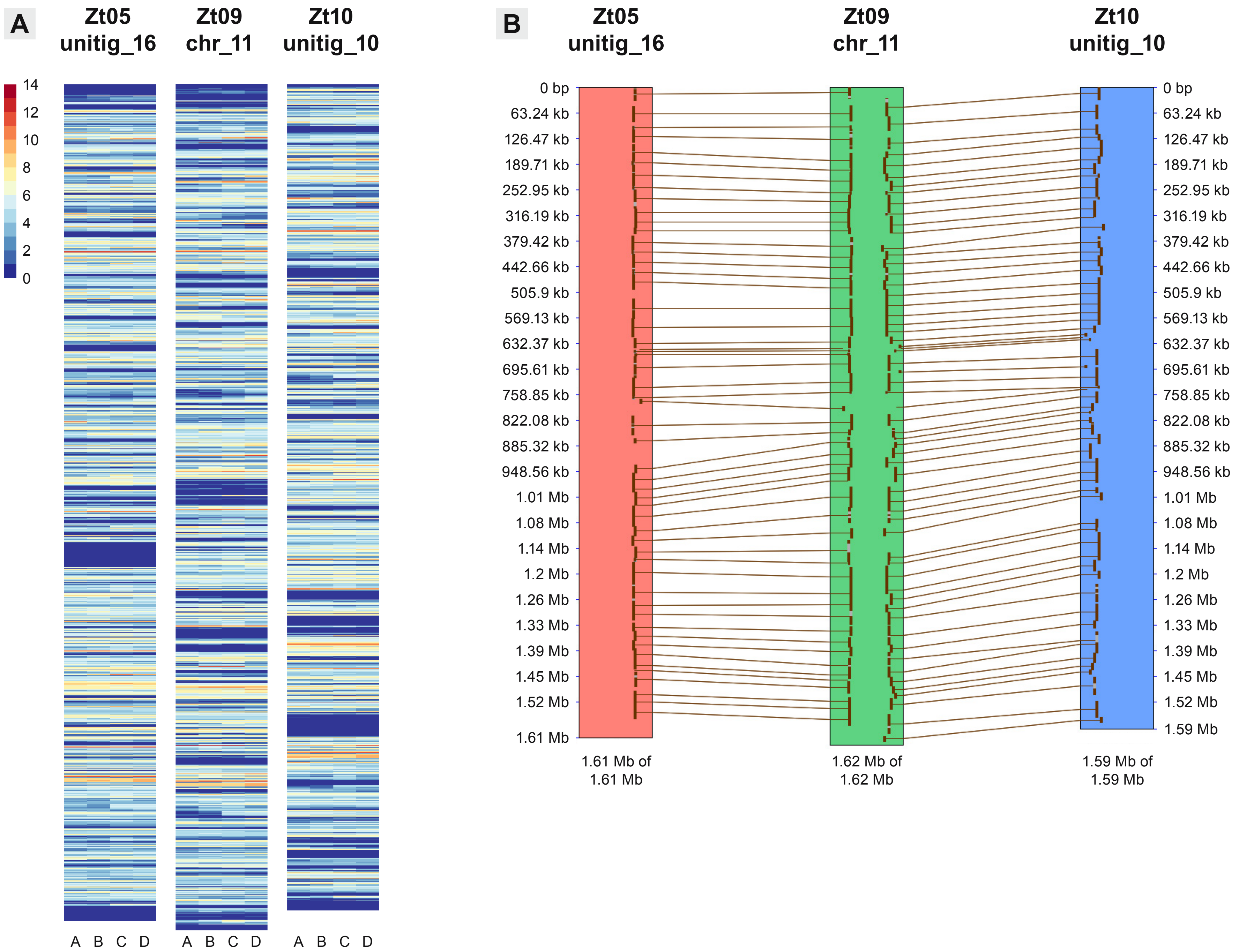

A
Zt05 unitig_16
Zt09 chr_11
Zt10 unitig_10
14
12
10
8
6
4
2
0
A B C D
B
Zt05 unitig_16
Zt09 chr_11
Zt10 unitig_10
0 bp
63.24 kb
126.47 kb
189.71 kb
252.95 kb
316.19 kb
379.42 kb
442.66 kb
505.9 kb
569.13 kb
632.37 kb
695.61 kb
758.85 kb
822.08 kb
885.32 kb
948.56 kb
1.01 Mb
1.08 Mb
1.14 Mb
1.2 Mb
1.26 Mb
1.33 Mb
1.39 Mb
1.45 Mb
1.52 Mb
1.61 Mb
1.59 Mb
1.61 Mb of 1.61 Mb
1.62 Mb of 1.62 Mb
1.59 Mb of 1.59 Mb

Figure S17

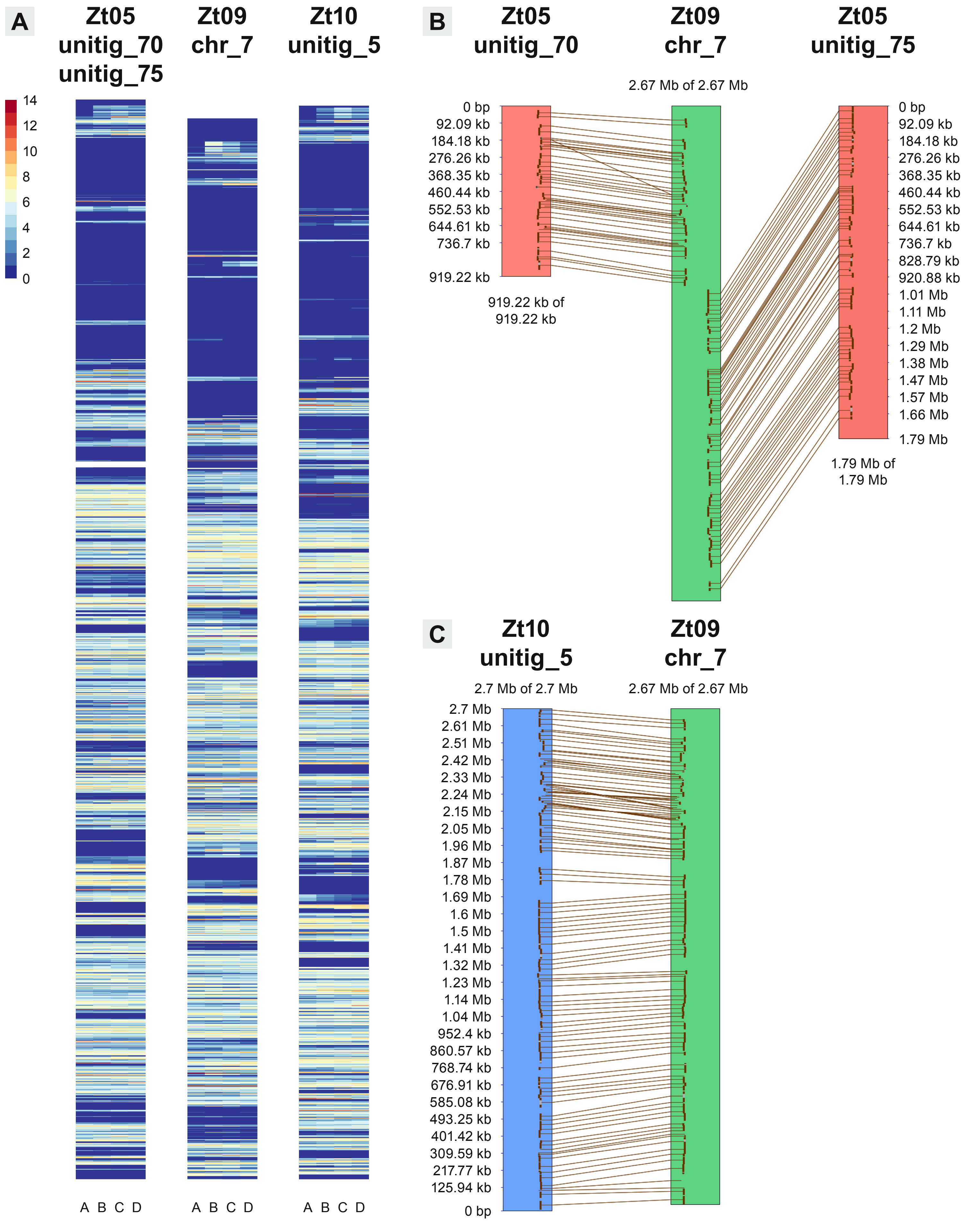
A
Zt05 unitig_70 unitig_75
Zt09 chr_7
Zt10 unitig_5
14
12
10
8
6
4
2
0
A B C D
A B C D
A B C D
B
Zt05 unitig_70
Zt09 chr_7
Zt05 unitig_75
2.67 Mb of 2.67 Mb
0 bp
92.09 kb
184.18 kb
276.26 kb
368.35 kb
460.44 kb
552.53 kb
644.61 kb
736.7 kb
919.22 kb
919.22 kb of 919.22 kb
0 bp
92.09 kb
184.18 kb
276.26 kb
368.35 kb
460.44 kb
552.53 kb
644.61 kb
736.7 kb
828.79 kb
920.88 kb
1.01 Mb
1.11 Mb
1.2 Mb
1.29 Mb
1.38 Mb
1.47 Mb
1.57 Mb
1.66 Mb
1.79 Mb
1.79 Mb of 1.79 Mb
C
Zt10 unitig_5
Zt09 chr_7
2.7 Mb of 2.7 Mb
2.67 Mb of 2.67 Mb
2.7 Mb
2.61 Mb
2.51 Mb
2.42 Mb
2.33 Mb
2.24 Mb
2.15 Mb
2.05 Mb
1.96 Mb
1.87 Mb
1.78 Mb
1.69 Mb
1.6 Mb
1.5 Mb
1.41 Mb
1.32 Mb
1.23 Mb
1.14 Mb
1.04 Mb
952.4 kb
860.57 kb
768.74 kb
676.91 kb
585.08 kb
493.25 kb
401.42 kb
309.59 kb
217.77 kb
125.94 kb
0 bp

Figure S18

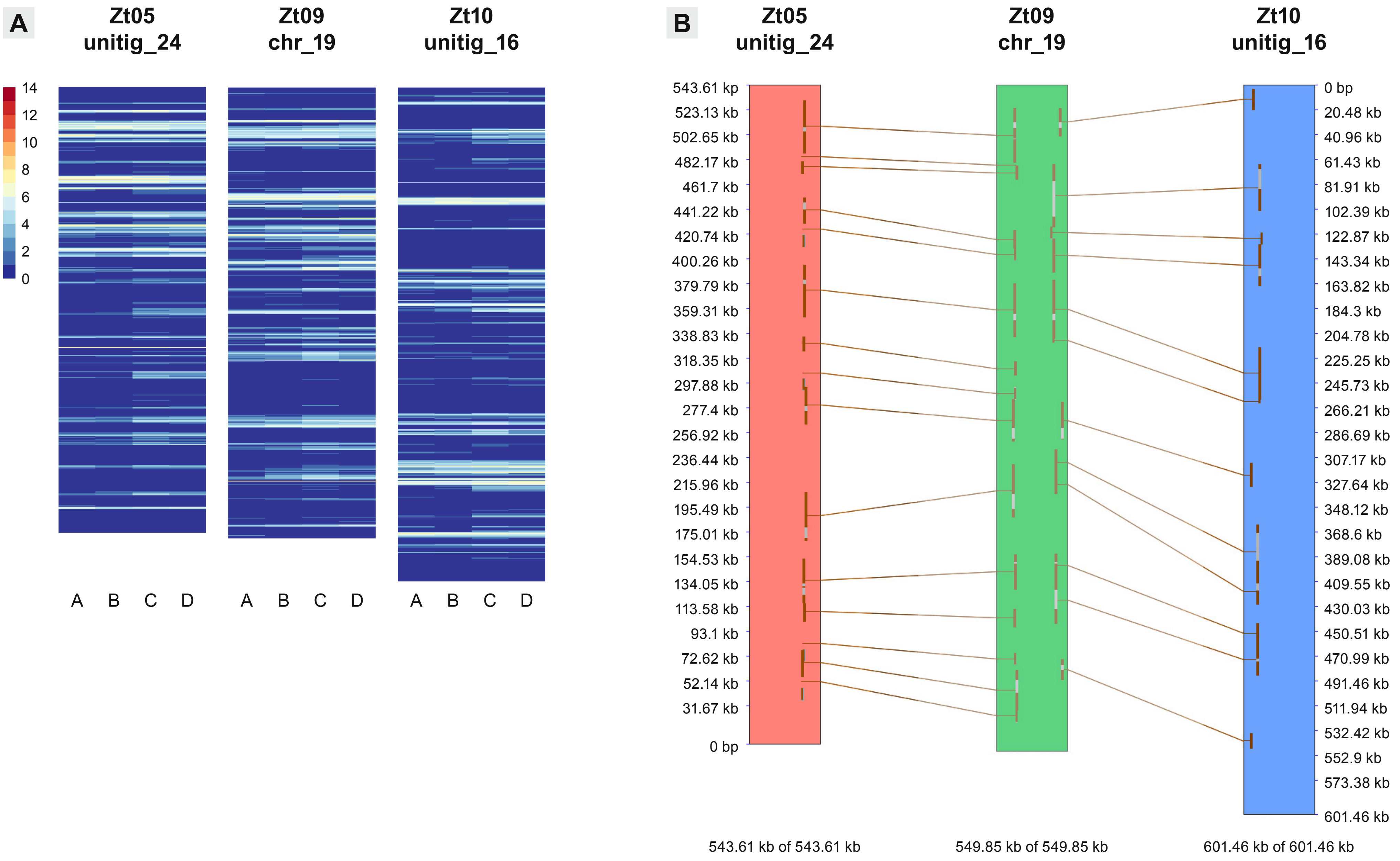

A
Zt05
unitig_24
Zt09
chr_19
Zt10
unitig_16
14
12
10
8
6
4
2
0
A B C D
A B C D
A B C D
B
Zt05
unitig_24
Zt09
chr_19
Zt10
unitig_16
543.61 kp
523.13 kb
502.65 kb
482.17 kb
461.7 kb
441.22 kb
420.74 kb
400.26 kb
379.79 kb
359.31 kb
338.83 kb
318.35 kb
297.88 kb
277.4 kb
256.92 kb
236.44 kb
215.96 kb
195.49 kb
175.01 kb
154.53 kb
134.05 kb
113.58 kb
93.1 kb
72.62 kb
52.14 kb
31.67 kb
0 bp
0 bp
20.48 kb
40.96 kb
61.43 kb
81.91 kb
102.39 kb
122.87 kb
143.34 kb
163.82 kb
184.3 kb
204.78 kb
225.25 kb
245.73 kb
266.21 kb
286.69 kb
307.17 kb
327.64 kb
348.12 kb
368.6 kb
389.08 kb
409.55 kb
430.03 kb
450.51 kb
470.99 kb
491.46 kb
511.94 kb
532.42 kb
552.9 kb
573.38 kb
601.46 kb
543.61 kb of 543.61 kb
549.85 kb of 549.85 kb
601.46 kb of 601.46 kb
