# Supplementary material for: Highly flexible infection programs in a specialized wheat pathogen
Source: Ecol Evol. 2018 Dec 26;9(1):275–94. doi: 10.1002/ece3.4724 (PMC6342133; doi:10.1002/ece3.4724)
Supplement: Supplementary file 9 [file ECE3-9-275-s009.docx]

**Table S8. The three *Z. tritici* isolates vary in tolerance to abiotic stressors.**

|  | 20/22C° at 16-h day / 8-h night | 28C° | 2 mM H_2_O_2_ | 3 mM H_2_O_2_ | 1 M sorbitol | 1 M NaCl | 500 µg/mL Congo red | 200 µg/mL calcofluor white |
| --- | --- | --- | --- | --- | --- | --- | --- | --- |
| Zt05 | - | + | ++ | +++ | + | ++ | - | - |
| Zt09 | - | - | - | + | + | ++ | + | + |
| Zt10 | ++ | +++ | + | ++ | + | ++ | + | + |

Summary of the *in vitro* stress assay comparing tolerance of the *Z. tritici* isolates Zt05, Zt09, and Zt10 to abiotic stressors. Symbols indicate tolerance levels in comparison to growth on YMS control plates at 18°C: - isolate was not affected, + isolate was mildly sensitive, ++ isolate was moderately sensitive, +++ isolate was highly sensitive.
