# Supplementary material for: Highly flexible infection programs in a specialized wheat pathogen
Source: Ecol Evol. 2018 Dec 26;9(1):275–94. doi: 10.1002/ece3.4724 (PMC6342133; doi:10.1002/ece3.4724)
Supplement: Supplementary file 22 [file ECE3-9-275-s022.docx]

**Supplementary Information – Text S1**

**Highly flexible infection programs in a specialized wheat pathogen**

Janine Haueisen^1,2^, Mareike Möller^1,2^, Christoph J. Eschenbrenner^1,2^, Jonathan Grandaubert^1,3^, Heike Seybold^1,2^, Holger Adamiak^2^ and Eva H. Stukenbrock^1,2^*

^1^Environmental Genomics Group, Max Planck Institute for Evolutionary Biology, August-Thienemann-Str. 2, 24306 Plön, Germany ^2^Environmental Genomics Group, Christian-Albrechts University Kiel, Am Botanischen Garten 1-11, 24118 Kiel, Germany **^3^**Fungal Biology and Pathogenicity, Institute Pasteur, Paris, France

*Corresponding author

**Supplementary Results and Discussion**

***Zymoseptoria tritici* isolates tolerate different levels of abiotic stress**

*Zymoseptoria tritici* is characterized by a dimorphic lifestyle with hyphal growth during host infection and predominantly yeast-like growth in *in vitro* cultures (Quaedvlieg *et al.*, 2011). The yeast/hyphae dimorphism is likely inherited as a multigenic quantitative trait (Lendenmann *et al.*, 2016) and is essential for pathogenicity (Mehrabi *et al.*, 2006). The fungus is exposed to a multitude of environmental influences during infection, dispersal, and other less well characterized stages of the life cycle such as saprotrophic growth and spore dormancy. We compared tolerance of the three *Z. tritici* isolates to several abiotic stressors *in vitro* (temperature, oxidative, osmotic, and cell wall stresses) to assess the variability in growth phenotypes (see Text S1 for Materials and Methods). Colonies of the three isolates exhibited different morphologies and tolerated different levels of abiotic stress (Fig S3; Table S8). Only osmotic stress led to the same level of reduced growth in all strains. Under all tested conditions, colonies of Zt09 and Zt10 were mainly composed of yeast-like cells, whereas Zt05 predominantly grew as hyphae. On plates supplemented with the anionic dyes Congo red or calcoflour white, Zt05 formed hyphal colonies comparable to those formed on yeast-malt-sucrose (YMS) control plates, whereas Zt09 and Zt10 were growth-impaired. Both tested dyes interfere with the fungal cell wall assembly by binding to chitin and thereby inhibiting the assembly of chitin‒β-glucan connections. Increased resistance can be associated to a lower chitin content in the fungal cell wall (Ram & Klis, 2006) why these results hint at differences in the cell wall composition of yeast-like and hyphal cells. However, yeast-like *Z. tritici* cells might in general be more susceptible to the cell wall-interfering agents as they were also suggested to inhibit chitinases required for cell separation in the budding yeast *Saccharomyces cerevisiae* (Roncero & Duran, 1985).

Elevated temperatures greatly impact development of Zt10 that formed strongly melanized colonies at 20/22°C and 28°C. Besides virulence-related functions (Perez-Nadales *et al.*, 2014), cell wall-associated fungal melanins can facilitate protection against harsh environmental conditions (Butler & Day, 1998). Zt10 was collected in the Ilam Province, a semi-arid, hot region in Iran (Pauw *et al.*, 2014). Possibly, increased melanization reflects local adaptation to extreme temperatures and temperature fluctuations (Zhan & McDonald, 2011), desiccation, and increased UV radiation.

Oxidative stress eventually diminishes growth of all isolates, although Zt09 tolerated exposure to H_2_O_2_ more than Zt05 and Zt10. *Z. tritici* experiences oxidative stress *in planta* from ROS produced as a host defense response or released from dead tissue. In general, higher tolerance to ROS is advantageous (Shetty *et al.*, 2007), for example during necrotrophic growth and pycnidia formation in dead mesophyll tissue (Shetty *et al.*, 2003). However, mechanisms to detoxify extracellular ROS must be tightly regulated to avoid ROS levels that are toxic to the hosts (Heller & Tudzynski, 2011), and we speculate that the observed differences in H_2_O_2_ tolerance reflect divergent adaptation of the *Z. tritici* isolates to host populations with different defense responses to pathogen invasion. Together, the *in vitro* stress assay revealed unanticipated intra-species variation in tolerance to abiotic stresses among the *Z. tritici* isolates, especially considering that all were isolated in agro-ecosystems from the same host species, *Triticum aestivum*. The variation in colony morphology and stress responses may reflect different adaptations of the *Z. tritici* isolates to their local environments, and our observations suggest that ecological adaptation of fungal plant pathogens can be a strong driver of phenotypic divergence.

**Karyotypes and synteny analyses of the three *Z. tritici* isolates**

Putatively dispensable chromosomes in the size range of 225 to 1,125 kb were separated by pulsed-field gel electrophoresis (PFGE) and visualized for the three *Z. tritici* isolates (Fig S4). We observed very different karyotypes with no small chromosomes of the same size (Fig S4). Further, the PFGE results suggest that Zt05 and Zt10 possess at least seven and four putative accessory chromosomes, respectively and show length polymorphisms of the smallest core chromosomes 12 (~1.463 kb) and 13 (~1,186 kb) compared to Zt09, consistent with a previous study (Mehrabi *et al.*, 2007). The previously reported loss of chromosome 18 (~574 kb) in Zt09 (Kellner *et al.*, 2014) could not be demonstrated by PFGE, as the chromosome could not be separated from the chromosomes 17 (~584 kb) and 16 (~607 kb) with almost the same size.

We observed intense chromosomal bands around 540 kb and 710 kb in Zt05 and around 615 kb in Zt10 (Fig S4). This indicates that both isolates possess additional chromosomes to the seven (Zt05) and four (Zt10) chromosomes that we identified based on separated chromosomal bands by PFGE. Indeed, analyses of *de novo* genome assemblies based on long-read SMRT Sequencing data for Zt05 and Zt10, show eight and five mainly full chromosome unitigs (indicated by telomeric repeats at both ends) in the size range of 290 to 905 kb (Table S9) with synteny to IPO323/Zt09 chromosomes 14 to 21 (Fig S5).

On the PFGE gel, we identified a chromosomal band around 640 kb for Zt10 that possibly represents unitig 15 (634 kb). This unitig shares no synteny with an IPO323 chromosome suggesting that this is a hitherto not described accessory chromosome in the species. However, as there is only one telomeric repeat present at one end of unitig 15 the assembly does not represent the full chromosome. Moreover, transcribed regions on unitig 15 are syntenic to a larger block on Zt05 unitig 20 that was identified as homologous to chromosome 18 of IPO323. We further conducted blast searches with the sequences of the transcribed regions on unitig 15 and received hits for genes on chromosomes 18, 20, and 21 of the reference IPO323/Zt09 indicating breakage of macrosynteny (Hane *et al.*, 2011).

**Comparative analysis of Z*. tritici* infection development by confocal microscopy**

We set out to characterize the infection development of the three *Z. tritici* isolates Zt05, Zt09 and Zt10 on the surface as well as within wheat leaves. To this end, we conducted a detailed survey where we analyzed leaf material harvested at at 3-14, 17, 19-21, 24, 25, and 28 days after inoculation (dpi) by confocal laser-scanning microscopy. We used large z-stacks of longitudinal optical sections to reconstruct the spatial and temporal fungal colonization outside and within infected tissue. We first focused on shared characteristics of the three *Z. tritici* isolates during host colonization and reproduction. Thereby, as described in details below, we identified four distinct infection stages that we define as the core *Z. tritici* infection program (Fig 3). Furthermore, we characterized the differences and isolate-specific aspects of infection development of the three isolates including temporal, spatial, and quantitative variation of host colonization.

**The shared core infection program of *Z. tritici* is characterized by four infection stages**

The first infection stage A is the penetration of wheat leaf tissue by *Z. tritici* hyphae. Germination of fungal cells on the leaf surface is initiated and developing infection hyphae enter wheat stomata. We observed that germ tubes emerge from *Z. tritici* cells at different time points post inoculation indicating that the fungi sense and respond to particular host-derived cues that trigger the developmental switch from spores to hyphal growth (Turrà *et al.*, 2015). Germ tubes develop into filaments of which some grow directed towards stomatal openings and enter the leaf (Fig 3A, stage A, Animation S1 and S2). Occasionally, we noticed slight, spatially restricted swelling of hyphae on top of stomata that resemble primitive appressoria as also previously reported (Cohen *et al.*, 1993; Kema *et al.*, 1996). However, we never observed a direct penetration of epidermal cells. During stomatal passage and in the sub-stomatal cavities, *Z. tritici* infection hyphae grow in tight contact to the wheat guard cells. The close physical contact between hyphae and plant cells might facilitate delivery of *Z. tritici* effector molecules (Lo Presti *et al.*, 2015) or serve as a structural scaffold to direct fungal hyphae in the host tissue (Tucker & Talbot, 2001). However, not all inoculated *Z. tritici* cells caused stomatal penetrations. We found that a portion of cells did not form germ tubes within 28 dpi and that development of filaments was stopped before entering stomata; what we also expect to happen in field infections.

The subsequent infection stage B is characterized by biotrophic growth of *Z. tritici* and the symptomless colonization of wheat mesophyll (Fig 3A, stage B, Animation S3 and S4). For successful infections, the pathogen must avoid recognition by the host immune system and/or suppress activation of defense responses during biotrophic growth. We observed strict intercellular hyphal colonization, starting from sub-stomatal cavities into adjacent mesophyll tissue, whereat the hyphae grow in close contact with host cells. Remarkably, hyphae first grow in the interspace of epidermis and first mesophyll layer. There, hyphae spread in the grooves between adjacent epidermal cells and only subsequently explore subjacent mesophyll cell layers.

The transition from symptomless biotrophic to necrotrophic colonization and the development of disease symptoms like chlorosis and necrotic lesions represent the third infection stage C (Fig 3A, stage C). From there on, *Z. tritici* colonizes a biochemically changing host environment (Fig 2C) and feeds on nutrients released by the host cell death to build pycndia. Hyphae are branching and grow in all mesophyll layers, surrounding individual wheat mesophyll cells. Simultaneously, primal structures of the asexual fruiting bodies, the pycnidia, are established and begin to develop. Hyphae form ring-like scaffolds in the sub-stomatal cavities where hyphae align and build stromata (Animation S5-S7) that later give rise to conidiogenous cells.

The last stage D concludes the infection and is characterized by necrotrophic colonization and asexual reproduction (Fig 3A, stage D). *Z. tritici* hyphae eventually grow in an environment that is very nutrient rich and attractive to other microbial competitors, but also putatively toxic e.g. due to high concentrations of reactive oxygen species (Fig 2B and C). In necrotic leaf regions, the dead mesophyll tissue is heavily colonized and the asexual fruiting bodies are visible and maturated (Animation S8 and S9). Hyphae wrap around dead, collapsed mesophyll cells several times. The pathogen may keep this tight contact to the degrading plant cells to increase the acquisition of nutrients and maybe also to protect them from competing saprotrophic species. Sub-stomatal cavities within the colonized leaf areas are occupied by sub-globose pycnidia that can grow into the adjacent mesophyll tissue. Mature pycnidia harbour hyaline, oblong asexual pycnidiospores that are released through the former stomatal opening.

In general, the described infection stages of *Z. tritici* can be well distinguished by considering the majority of all infection events within inoculated leaf regions. However, we also observed that different infections stages are present simultaneously within one leaf. Infections by individual *Z. tritici* cells occur within a temporal range after inoculation and are not fully synchronized. Moreover, environmental influences and host physiological processes act differently on individual leaves and plants which also can lead to the temporal variation in infection development of *Z. tritici*.

During biotrophic colonization of wheat tissue, fluorescence emitted from fluorescein isothiocyanate conjugated to wheat germ agglutinin (WGA-FITC) primarily came from septa and was weak compared to that during necrotrophic colonization, during which fluorescence was also emitted from interseptal regions. Previously, similar observations were reported in endophytic and epiphyllous (Becker *et al.*, 2016) and biotrophic and necrotrophic hyphae (Takahara *et al.*, 2016). WGA binds to N-acetylglucosamine residues that build chitin, an elicitor of plant immunity (Sánchez-Vallet *et al.*, 2015). Fungal plant pathogens can prevent recognition, e.g. through chitin-binding LysM effectors (van den Burg *et al.*, 2006; de Jonge *et al.*, 2010; Sánchez-Vallet *et al.*, 2013) like the extracellular LysM protein ChELP2, that was also shown to limit accessibility of chitin to WGA in biotrophic hyphae of *C. higginsianum* (Takahara *et al.*, 2016). In *Z. tritici*, two LysM effectors protect hyphae from plant chitinases (Marshall *et al.*, 2011), and Mg3LysM shields chitin from recognition by wheat receptors (Lee *et al.*, 2013). Hence, it is possible that Mg3LysM also limits binding of WGA to chitin during biotrophic, but not necrotrophic, colonization of *Z. tritici* leading to the differences in fluorescence signals from biotrophic and necrotrophic hyphae.

**Highly differentiated infection phenotypes of the three *Z. tritici* isolates on Obelisk wheat**

Although we clearly recognize the four core infection stages for the three *Z. tritici* isolates, we found that the infection phenotypes of Zt05, Zt09, and Zt10 are highly differentiated. We observed temporal, spatial and quantitative variation in the infection development of these isolates on the wheat cultivar Obelisk.

The duration of the initial infection stage A—in particular the period between inoculation and stomatal penetrations—is different in the three *Z. tritici* isolates. For Zt05, infection hyphae enter stomata within 5 dpi. Germ-tube formation and stomatal penetration is usually slower for Zt09 (up to 8 dpi) and most delayed for Zt10 (up to 10 dpi) (Fig 3A, stage A). We also noticed strong epiphyllous proliferation and mycelium formation for Zt05 during all infection stages and frequently, several infection hyphae of Zt05 enter one stoma (Fig 3A, stage A: Zt05). In general, hyphae of Zt05 and Z09 penetrate stomata at high frequencies, while we saw fewer stomatal penetrations for the Zt10 leading to patchy infections within the inoculated leaf areas (Fig 3A, stage C and D: Zt10).

The extent of biotrophic colonization during infection stage B comprises the most pronounced difference between the three isolates. Zt05 builds biotrophic hyphal networks in the mesophyll tissue with long “runner” hyphae growing primarily longitudinally between epidermis and mesophyll (Fig 3A, stage B: Zt05, Fig 3B.1, Animation S3). Biotrophic hyphal networks of Zt09 are smaller and located mainly in the interspace of epidermis and mesophyll as well as between the cells of the upper mesophyll layer (Fig 3A, stage B: Zt09, Fig 3B.2). Biotrophic colonization by Zt10, however, is very poor and hyphal growth is limited to the mesophyll cells adjacent to sub-stomatal cavities (Fig 3A, stage B: Zt10, Fig 3B.3). Since biotrophic colonization depends on successful evasion of host immunity (Jones & Dangl, 2006), the different extent of colonization could reflect different strategies to bypass recognition in a given host genotype.

During the later infection stages, differences between the isolates are smaller and primarily relate to temporal variation. Transition to necrotrophic growth usually first occurs for Zt05 (9 to 14 dpi), followed by Zt09 (13 to 16 dpi), and Zt10 (13 and 17 dpi) (Fig 3A, stage C). Studying the development of the asexual fruiting bodies, we frequently noticed the formation of two pycnidia in one sub-stomatal cavity for Zt10 (Fig 3A, stages C and D: Zt10, Animation S7). This was observed less often for the other two isolates. The onset of infection stage D occurs in the same temporal order as for stage C, first for Zt05, followed by Zt09, and last by Zt10. At 28 dpi, inoculated leaf areas are usually fully necrotic for Zt09 and frequently covered by several distinct necrotic lesions for Zt10 (Fig S7).

Taken together, we observed highly differentiated infection phenotypes for the three *Z. tritici* isolates due to isolate-specific infection development. However, the final production of asexual pycnidia did not differ significantly between the three isolates (Fig 1), suggesting that the isolate-specific aspects in host-pathogen interaction sum up to equally good strategies for host colonization and asexual reproduction. We conclude that infection development of *Z. tritici* can be highly flexible with respect to the timing of the lifestyle transition and the spatial distribution of infecting hyphae inside host tissue.

With several independent plant infection experiments using the three isolates Zt05, Zt09, and Zt10 on the wheat cultivar Obelisk, we found that the temporal disease progress and, consequently, the duration of the different infection can stages vary between experiments. However, although the precise timing for the onset of the four stages can differ between experiments, the relative temporal differences between the isolates, as described above, remain consistent.

**Percentage of mapped RNA-seq reads reflects infection stage-specific fungal biomass**

For transcriptome datasets representing initial infection (stage A) and biotrophic growth (stage B), where comparably little fungal biomass is present and the wheat tissue is still fully intact, on average 8.02% and 8.2% of the filtered reads were aligned to the fungal genomes (Tables 1, S3). Exceptionally high alignment rates were obtained for isolate Zt05, (average stage A: 13.52%, average stage B: 12.41%), likely reflecting the strong proliferation on the leaf surface as well as the expanded biotrophic hyphal networks (Fig 3: Zt05, Animation S3). For RNA-seq samples covering the lifestyle transition (stage C) and necrotrophic growth (stage D), where *Z. tritici* hyphal networks rapidly expand and the wheat mesophyll cells die, the amount of fungal-derived reads increased to 23.95% and 55% on average, respectively. The constant increase in fungal-derived RNA-seq reads during wheat infection reflects the increase in fungal biomass within the leaf tissue due to mesophyll colonization and lifestyle transition.

**Core *Zymoseptoria tritici* transcriptional program during wheat infection**

We performed differential gene expression analyses to compare expression of the 10,426 *Z. tritici* core genes. We identified 597 genes that were differentially expressed between the infection stages (DESeq2, *P*_adj_ ≤ 0.01, |log_2_ fold change| ≥2) and show the same expression kinetics in all three isolates (Fig 4A). Interestingly, 79 of these genes were differentially expressed between several infection stages, suggesting dynamic, wave-like expression kinetics (Fig S12). A total of 246 genes were differentially expressed (Table S12) between stage A and stage B; the vast majority of these (242) were up-regulated in stage B. In stage A, three of the four genes that were up-regulated encode candidate secreted effector proteins (CSEPs), and the fourth encodes a carbohydrate active enzyme (CAZyme) similar to an extracellular chitosanase (*Zt09_chr_11_00040*). This gene is significantly down-regulated or not expressed during later infection (stages B to D), suggesting a role of the enzyme during early establishment in the leaf, similar to the role of a homolog described in *Fusarium solani* (Liu *et al.*, 2010). Another gene (*Zt09_chr_6_00**402*) that was strongly up-regulated in all isolates during early infection encodes a putative hsp30-like small heat shock protein, possibly reflecting a response to stressful environmental conditions on the wheat leaf surface (Haslbeck & Vierling, 2015).

The 242 genes up-regulated during biotrophic colonization are enriched with Gene Ontology (GO) groups involved in proteolysis (GO:0006508; 27 genes) and amino acid transmembrane transport (GO:0003333; 5 genes) (*P* < 0.01, Fischer’s exact test). Furthermore, three previously characterized LysM homologs (Marshall *et al.*, 2011) and two homologs (*Zt09_chr_11_00358*, *Zt09_chr_13_00167*) of *Ecp2*, an effector gene of the tomato-infecting fungus *Cladosporium fulvum* (Laugé, 1997), are also strongly up-regulated during early infection, emphasizing the importance of these genes for biotrophic colonization. PFAM domain analysis further shows enrichment of genes encoding cytochrome P450- and polyketide synthase-like proteins that possibly play a role in the production of secondary metabolites (*P* < 0.001, χ^2^ test).

In stage B, 22 genes are up-regulated compared to stage C (Table S13), including four genes encoding CSEPs of unknown function and a gene encoding the putative non-secreted catalase *Zt09_chr_6_00289*. Metabolite profiling showed that oxidative catabolism of lipids plays an important role for *Z. tritici* during biotrophic colonization (Rudd *et al.*, 2015). High catabolic activity in the peroxisome entails accumulation of H_2_O_2_, which likely requires high abundance of catalase to maintain cellular redox homeostasis. *Zt09_chr_10_00421* is also highly expressed during biotrophic growth and down-regulated at later infection stages. It encodes a protein similar to siderophore iron transporter 1, previously described to be involved in the uptake of iron (Yun *et al.*, 2000) which is essential for fungal growth and pathogenesis (Haas *et al.*, 2008).

In stage C, 334 genes are significantly up-regulated compared to stage B (Table S13) and 58 genes in comparison to stage D (Table S14). Genes up-regulated from B to C are enriched with GO groups involved in metabolic processes (GO:0008152; 97 genes), in particular L-arabinose metabolic processes (GO:0046373; 4 genes), and transmembrane transport (GO:0055085; 25 genes). Similarly, a PFAM analysis shows an enrichment of genes encoding transporters; CAZymes including different groups of glycosyl hydrolases, serine hydrolases, alpha-L-arabinofuranosidases and cutinases that play important roles as plant tissue and cell wall degrading enzymes (Kubicek *et al.*, 2014); polyketide synthases; and cytochrome P450s. This transcriptional reprogramming reflects the physiological changes that *Z. tritici* undergoes during the transition from biotrophic to necrotrophic growth and is consistent with our microscopic observations. Among the 58 genes down-regulated from C to D (Table S14) we identified GO groups involved in arabinan metabolic processes (GO:0031221; one gene) and an enrichment of PFAM domains related to beta-ketoacyl-ACP synthases, which are known to be involved in fatty acid production and important for the generation of new cell membrane, as well as cytochrome P450s, polyketide synthases, hydrophobic surface binding protein A (Ohtaki *et al.*, 2006), and tyrosinases.

Only 16 genes were significantly up-regulated from stage C to D, which is when the pycnidia mature (Table S14), indicating overall similar transcription profiles during the two necrotrophic stages. Genes that are up-regulated during necrotrophic growth and reproduction are predicted to encode proteins similar to CAZymes, transporters, and proteins containing RNA-binding domains. Up-regulation of the secreted catalase-like protein-encoding gene *Zt09_chr_5_00821* shows the importance of detoxification of the ROS H_2_O_2_, which is highly abundant in necrotic leaf tissue as shown by DAB staining (Fig 2C).

In summary, we identified a core set of genes that show the same expression pattern in the three isolates during infection development. This core set includes genes encoding putative effectors as well as enzymes predicted to play a role in the breakdown and metabolism of plant cell components.

**Supplementary Materials and Methods**

**Phenotypic assays *in vitro***

To compare tolerance towards stress conditions and assess the *in vitro* phenotypes of the *Z. tritici* isolates, we conducted a stress assay as previously described (Poppe *et al.*, 2015). After five days, we compared growth on solid YMS medium at 18°C to growth on YMS medium exposed to stress conditions: temperature (20/22°C with 16-h day/8-h night rhythm, 28°C in darkness), oxidative stress (2 and 3 mM H_2_O_2_), osmotic stress (1 M NaCl, 1 M sorbitol), and cell wall stress (500 µg/mL Congo red, 200 µg/mL calcofluor white). Colony development was documented using a Canon EOS 600D. Each stress treatment was replicated three times.

**Detection of H_2_O_2_ in *Z. tritici* infected wheat leaves**

To visualize and localize the accumulation of the reactive oxygen species H_2_O_2_ within *Z. tritici* infected leaf tissue, we conducted 3,3’-diaminobenzidine (DAB) staining (Thordal-Christensen *et al.*, 1997) at 2, 4, 7-11, 14, 16, 18, and 21 days post inoculation (dpi). Inoculated leaf parts were excised with a razorblade and immersed in DAB solution (1 mg/mL 3,3’‑diaminobenzidine tetrahydrochloride (Thermo Fisher Scientific, Rockford, USA) in 0.05% [v/v] Tween 20). Samples were protected from light and DAB solution was infiltrated in two steps: 1^st^ at low pressure (600 mbar) for two times 15 min and 2^nd^ at gentle shaking (22 rpm) for 90 min. Subsequently, leaf samples were incubated overnight in de-staining solution (96% ethanol: acetic acid = 3:1 [v/v]) at gentle shaking (25 rpm). Cleared samples were stored in 96% ethanol and examined in 40% glycerol. Presence of H_2_O_2_ is indicated by reddish-brown precipitate in cleared leaf tissue. Infected leaf samples were documented by an iPhone 7 camera prior to the DAB staining and by a Canon EOS 600D post staining. Stained leaves were scanned at a resolution of 2,400 dpi using a HP Photosmart C4580 flatbed scanner. Leaf images were analyzed using ImageJ (Schneider *et al.*, 2012) and a macro script customized from (Stewart *et al.*, 2016) to quantify leaf area containing brown precipitate.

**Staining of infected wheat leaves and confocal laser-scanning microscopy**

Infected leaf parts were excised and de-stained in 96% ethanol. Samples were transferred to 10% KOH [w/v] at 85°C for 3 min to increase tissue permeability. For neutralization, leaf material was washed three times with 1X phosphate-buffered saline (PBS, pH 7.4) and subsequently incubated in a staining solution of 0.02% Tween 20 in 1X PBS (pH 7.4) with 10 µg/mL wheat germ agglutinin conjugated to fluorescein isothiocyanate (WGA-FITC) and 20 µg/mL propidium iodide (PI). Samples were protected from light and the staining solution was vacuum-infiltrated for 2 h where pressure was continuously reduced to 400 mbar for 5 min followed by ventilation of the desiccator and return to standard pressure. The staining solution was replaced by 1X PBS (pH 7.4) and the stained leaf samples were directly subjected to confocal microscopy analysis or stored lightproof at 4°C for later use. WGA was used to specifically label fungal hyphae (Robin *et al.*, 1986) but was occasionally found to also bind to plant cell walls and bacteria. PI stains DNA and binds to plant and fungal cell walls (Rounds *et al.*, 2011). FITC was excited with an argon laser at 488 nm and fluorescence was detected between 500 and 540 nm. A diode-pumped solid-state laser at 561 nm was employed for excitation of PI and emission was detected from 600 to 670 nm. Image stacks were obtained with a *x/y* scanning resolution of 1024 x 1024 (Leica) or 1500 x 1500 pixels (Zeiss) and a step size of 0.5 - 1 µm in *z*.

**Generation of DNA plugs and karyotyping by pulsed-field gel electrophoresis**

A non-protoplast protocol was used to produce DNA plugs for separating small chromosomes (~0.2 - 1.6 Mb) by pulsed-field gel electrophoresis (PFGE) (Stukenbrock *et al.*, 2010). Single cells of the three *Z. tritici* isolates were harvested from liquid YMS cultures. For preparation of plugs, 5 x 10^8^ cells were used as input and embedded in 1.1% low range agarose (Bio-Rad). Solidified agarose blocks were incubated in lysis buffer (1% SDS, 0.45 M EDTA, 1.5 mg/mL Proteinase K (Roth)) at 55°C for 48 h and subsequently washed three times in 1X TE buffer for 20 min. Plugs were directly submitted to pulsed-field gel electrophoresis or stored in 0.5 M EDTA at 4°C until further use.

PFGE was conducted using a contour-clamped homogeneous electric field (CHEF)-DR III apparatus (Bio-Rad) in 1% agarose in 0.5X TBE buffer applying the following conditions: temperature 14°C, 120° angle, 5 V/cm with a ramped 50 - 150 s switching interval for 48 to 68 h. Chromosomal DNA of *Saccharomyces cerevisiae* (Bio-Rad) was used as standard size marker. Gels were stained for 30 min in 1 µg/mL ethidium bromide solution and chromosomal bands were detected with the Thyphoon Trio™ (GE).

***De novo* genome assemblies of Zt05 and Zt10**

High molecular weight DNA of Zt05 and Zt10 was extracted from single cells grown in liquid YMS, using a modified version of the cetyltrimethylammonium bromide (CTAB) extraction protocol (Allen *et al.*, 2006), and used as input to prepare Pacific Biosciences (PacBio) SMRTbell libraries that were size-selected with a 10- to 15-kb cut-off. Single-molecule real-time (SMRT) sequencing was performed on four SMRT cells and run on a PacBio RS II instrument at the Max Planck Genome Center in Cologne, Germany (http://mpgc.mpipz.mpg.de). Genome assemblies of Zt05 and Zt10 based on the generated PacBio long reads were done as previously described (Plissonneau *et al.*, 2016) using *HGAP* (Chin *et al.*, 2013) v3.0 included in the *SMRTanalysis suite* v2.3.0. Briefly, we applied default settings for *HGAP* runs and tested the influence of different minimum seed read lengths (13 kb, 15 kb, 19 kb, and 21 kb) used for initiation of self-correction. A 19-kb minimum seed read length cut-off generated the most favorable results in terms of pre-assembly yield, assembly N50, and length of total assembly. Assembled unitigs were polished by applying default settings of *Quiver,* which is part of the *SMRTanalysis suite*.

**Supplementary Tools and Commands**

**Image analysis to quantify H_2_O_2_-accumulation in *Z. tritici*–infected leaves**

**# Modification of batch-processing ImageJ macro**

An ImageJ (Schneider *et al.*, 2012) macro to analyze infection symptoms on wheat leaves (Stewart *et al.*, 2016) was modified to quantify leaf areas affected by ROS accumulation indicated by brown staining. Line 934 of original macro (version 01/2016) containing command run("Fill Holes"); was removed to allow detection of small unstained leaf regions within stained regions. Otherwise, macro settings were chosen to detect ROS-affected areas instead of necrosis within leaf area.

**# Macro settings**

Min0 Max0 Min1 Max1 Min2 Max2 colmode Pass_Stop_H Pass_Stop_S 1 0 255 0 255 0 230 0 1 1 Pass_Stop_B maximaNoise leafAreaUpper leafAreaLower lesionSize

1 25 255 127 0.001

**Tools and *commands* used for genome analyses and processing and analyses of**

***Z. tritici* transcriptome data**

**# Quality control of RNA sequencing data**

FastQC (www.bioinformatics.babraham.ac.uk/projects/fastqc/) version 0.11.2

**# Removal of residual TruSeq adapter sequences**

Trimmomatic (Bolger *et al.*, 2014) version 0.33

*java -jar /…/trimmomatic-0.33.jar SE -threads 1 -phred33* *\*

*reads.fastq* *reads-adap.fastq \*

*ILLUMINACLIP:/…/Trimmomatic/adapters/TruSeq3-SE.fa:2:30:15 MINLEN:100*

**# Trimming of 12 nucleotides at 5' end of all reads**

Trimmomatic (Bolger *et al.*, 2014) version 0.33

*java -jar /…/trimmomatic-0.33.jar SE -threads 1 -phred33 \*

*reads-adap.fastq reads-adap-trim.fastq \*

*HEADCROP:12*

**# Filtering of reads based on quality scores**

**At least 80 % of bases must have a quality score ≥ 20 or read was dropped.**

FASTX-toolkit (http://hannonlab.cshl.edu/fastx_toolkit/) version 0.0.14

*fastq_quality_filter -q 20 -p 80 -v -Q33 \*

*-i reads-adap-trim.fastq -o* *reads-adap-trim-filt.fastq*

**# Masking of low quality bases**

**Nucleotides with quality score < 20 were masked with ‘N’.**

FASTX-toolkit (http://hannonlab.cshl.edu/fastx_toolkit/) version 0.0.14

*fastq_masker -q 20 -r N -v -Q33 \*

*-i reads-adap-trim-filt.fastq -o reads-adap-trim-fil-maskt.fastq*

**# Mapping of reads to genomes of *Z. tritici* isolates**

TopHat2 (Trapnell *et al.*, 2012; Kim *et al.*, 2013) version 2.0.9

*tophat --b2-sensitive --read-mismatches=10 --read-gap-length=10 --read-edit-dist=20 \*

*--library-type=fr-firststrand \*

*-o /…/output_directory \*

*/…/reference_genome_index \*

*/…/reads-adap-trim-filt-mask.fastq*

**# Manipulation of RNA-seq read alignments**

SAMtools (Li *et al.*, 2009) version 0.1.19

*samtools view \
-o* */…/**read_alignment_accepted_hits.sam* *\**/…/ read_alignment_accepted_hits.bam*

*samtools sort \
/…/ read_alignment_accepted_hits.bam \**/…/ read_alignment_accepted_hits_sort*

*samtools index \
 /…/* *read_alignment_accepted_hits_sort.bam*

**# Calculation of relative gene expression levels among the four infection stages within one *Z. tritici* isolate**

Cuffdiff2 in Cufflinks (Trapnell *et al.*, 2013) version 2.2.1

*cuffdiff --library-type fr-firststrand --library-norm-method geometric \*

*--dispersion-method per-condition --FDR 0.001 \*

*–L Ztxx_stage_A,Ztxx_stage_B,Ztxx_stage_C,Ztxx_stage_D \*

*/…/Ztxx_genes.gff \*

*/…/**Ztxx_stage_A_rep1_ read_alignment_accepted_hits_sort.bam \*

*/…/ Ztxx_stage_A_rep2_ read_alignment_accepted_hits_sort.bam \*

*/…/Ztxx_stage_B_rep1_ read_alignment_accepted_hits_sort.bam \*

*/…/ Ztxx_stage_B_rep2_ read_alignment_accepted_hits_sort.bam \*

*/…/Ztxx_stage_C_rep1_ read_alignment_accepted_hits_sort.bam \*

*/…/ Ztxx_stage_C_rep2_ read_alignment_accepted_hits_sort.bam \*

*/…/Ztxx_stage_D_rep1_ read_alignment_accepted_hits_sort.bam \*

*/…/ Ztxx_stage_D_rep2_ read_alignment_accepted_hits_sort.bam \*

*-o /…/output_directory \*

**# Counting of mapped sequencing reads per gene**

HTSeq (Anders *et al.*, 2015) version 0.6.1p1

*htseq-count -m union --type=gene --idattr=Name --stranded=reverse \*

*/…/read_alignment_accepted_hits.sam \*

*/…/Ztxx_genes.gff \*

*> /…/Ztxx_genes_counts.txt*

**# Differential gene expression analyses**

R package DESeq2 (Love *et al.*, 2014) version 1.10.1

# Comparison between infection stages across all isolates

*ddsMatrix <- DESeqDataSetFromMatrix(countData = countdata, colData = condition.table,*

*design = ~ strain+stage)*

*dds <- DESeq(ddsMatrix, betaPrior = T, modelMatrixType = "expanded")*

*DE_genes_AB <- results(dds, contrast=c("stage", "B", "A"))*

*DE_genes_BC <- results(dds, contrast=c("stage", "C", "B"))*

*DE_genes_CD <- results(dds, contrast=c("stage", "D", "C"))*

# Comparison within infection stages between two isolates

*dds$group <- factor(paste0(dds$strain, dds$stage))*

*design(dds) <- ~ group*

*dds <- DESeq(dds)*

*DE_genes_Ztxx_Ztyy_stageA <- results(dds, contrast=c("group","ZtxxA", "ZtyyA"))*

**# Gene ontology (GO) term enrichment analyses**

R package topGO (Alexa *et al.*, 2006) version 2.28.0

**# Protein families (PFAM) enrichment analyses**

Custom python script

*#!/usr/bin/python*

*import os,sys,re,scipy.stats*

*import numpy as np*

*bck=open(os.path.abspath(sys.argv[2])).readlines()*

*subset=[i.rstrip() for i in open(os.path.abspath(sys.argv[1])).readlines()]*

*des=open("./pfam_desc.txt").readlines()*

*pfam_desc={}*

*for i in des:*

*i=i.rstrip()*

*s=i.split("\t")*

*if not pfam_desc.has_key(s[0]):*

*pfam_desc[s[0]]=s[2]*

*pfam_bck={}*

*pfam_subset={}*

*nb_gene_pfam_bck=0*

*nb_gene_pfam_subset=0*

*for line in bck:*

*s=line.rstrip().split()*

*gene=s[0]*

*if len(s)>1:*

*t=s[-1].split(";")*

*nb_gene_pfam_bck+=1*

*if gene in subset:*

*nb_gene_pfam_subset+=1*

*for i in t:*

*if not pfam_bck.has_key(i):*

*pfam_bck[i]=1*

*else:*

*pfam_bck[i]+=1*

*if gene in subset:*

*if not pfam_subset.has_key(i):*

*pfam_subset[i]=1*

*else:*

*pfam_subset[i]+=1*

*pfam_domains=pfam_bck.keys()*

*M=nb_gene_pfam_bck*

*N=nb_gene_pfam_subset*

*print "ACC\tDESC\tNB_PROT_DOMAIN_SET\tNB_PROT_DOMAIN_OTHER\tENRICHMENT\tPVALUE"*

*for domain in pfam_domains:*

*x=0*

*n=pfam_bck[domain]*

*if pfam_subset.has_key(domain):*

*x=pfam_subset[domain]*

*obs = np.array([[x, N-x],[n-x, M-N-(n-x)]])*

*fo=x/float(N)*

*fe2=(n-x)/float(M-N)*

*enrich=0.0*

*if fe2>0 and fo>0:*

*enrich=fo/fe2*

*pvalue_hypergeo=scipy.stats.hypergeom.sf(x,M,n,N)*

*chi2, pvalue_chi2, dof, ex = scipy.stats.chi2_contingency(obs)*

*if x>0 and enrich>1:*

*print "%s\t%s\t%s\t%s\t%.1f\t%.6f" %(domain,re.sub("\s","_",pfam_desc[domain]),x,n-x,enrich,pvalue_chi2)*

**# Calculation of genomic distances between genes and TEs / H3K9me3 and H3K27me3**

BEDtools (Quinlan & Hall, 2010) version 2.26.0

*bedtools closest -D a -t first \*

*-a* */…/Ztxx_genes.bed \*

*-b /…/Ztxx_features.bed \*

*> /…/Ztxx_genes_feature_distances.txt*

**# *De novo* genome assemblies of Zt05 and Zt10 based on PacBio long reads**

SMARTanalysis suite (Chin *et al.*, 2013) version 2.3.0

HGAP version 3.0

Quiver

*source Local_SMRTanalysis/current/etc/setup.sh*

*fofnToSmrtpipeInput.py HGAP.input.fofn > HGAP.input.xml*

*smrtpipe.py -D NPROC=2 -D MAX_THREADS=2 \*

*--output=Results_SMRT –params=HGAP.input.xml xml:HGAP.input.xml*

**# Synteny mapping and analyses of IPO323/Zt09 chromosomes and Zt05 and Zt10 unitigs**

SyMAP (Soderlund *et al.*, 2011) version 4.2

Applying default settings and running NUCmer and PROmer to compute raw hits for anchor clustering.

Minimal contig size: Zt05: 1,000 kb

Zt09: 100,000 kb

Zt10: 10,000 kb

Mugsy (Angiuoli & Salzberg, 2011) version 1.r2.2

Generation of pairwise genome alignments of IPO323 – Zt05 and IPO323 – Zt10 applying default settings of Mugsy.

Custom python script to extract unique DNA blocks with a minimum length of 1 bp and calculate the total amount of unique DNA.

alignment = maf_parse(args.maf)

min_len = int(args.min_len)

count = 0

if args.mode == "normal":

for mga in alignment:

strains_i = args.include.split(",")

for position in xrange(0, len(mga[0]["seq"]), 1):

temp_bin = []

all_there = 0

for record in mga:

if any(strain in record["id"] for strain in strains_i):

all_there += 1

temp_bin.append(record["seq"][position])

if all_there == len(strains_i) and len(temp_bin) == len(strains_i):

if int(mga[0]["len"]) >= min_len:

if "-" in temp_bin:

pass

else:

count += 1

print "%sbp conserved" % count

elif args.mode == "reverse":

for mga in alignment:

strains_i = args.include.split(",")

strains_e = args.exclude.split(",")

exclude_there = 0

all_there_except = 0

for record in mga:

if any(strain in record["id"] for strain in strains_i):

all_there_except += 1

elif any(strain in record["id"] for strain in strains_e):

exclude_there += 1

else:

pass

if all_there_except == len(strains_i) and exclude_there == 0:

if int(mga[0]["len"]) >= min_len:

count += int(mga[0]["len"])

print "%sbp unique" % count

**References**

**Alexa A, Rahnenführer J, Lengauer T**. **2006**. Improved scoring of functional groups from gene expression data by decorrelating GO graph structure. *Bioinformatics* **22**: 1600–1607.

**Allen GC, Flores-Vergara M a, Krasynanski S, Kumar S, Thompson WF**. **2006**. A modified protocol for rapid DNA isolation from plant tissues using cetyltrimethylammonium bromide. *Nature protocols* **1**: 2320–2325.

**Anders S, Pyl PT, Huber W**. **2015**. HTSeq—a Python framework to work with high-throughput sequencing data. *Bioinformatics* **31**: 166–169.

**Angiuoli S V., Salzberg SL**. **2011**. Mugsy: Fast multiple alignment of closely related whole genomes. *Bioinformatics* **27**: 334–342.

**Becker M, Becker Y, Green K, Scott B**. **2016**. The endophytic symbiont Epichloe festucae establishes an epiphyllous net on the surface of Lolium perenne leaves by development of an expressorium, an appressorium-like leaf exit structure. *New Phytologist* **211**: 240–254.

**Bolger AM, Lohse M, Usadel B**. **2014**. Trimmomatic: A flexible trimmer for Illumina sequence data. *Bioinformatics* **30**: 2114–2120.

**van den Burg H a, Harrison SJ, Joosten MHAJ, Vervoort J, de Wit PJGM**. **2006**. Cladosporium fulvum Avr4 protects fungal cell walls against hydrolysis by plant chitinases accumulating during infection. *Molecular plant-microbe interactions : MPMI* **19**: 1420–1430.

**Butler MJ, Day AW**. **1998**. Fungal melanins: a review. *Canadian Journal of Microbiology* **44**: 1115–1136.

**Chin C-S, Alexander DH, Marks P, Klammer AA, Drake J, Heiner C, Clum A, Copeland A, Huddleston J, Eichler EE, *et al.*** **2013**. Nonhybrid, finished microbial genome assemblies from long-read SMRT sequencing data. *Nature Methods* **10**: 563–569.

**Cohen L, Eyal Z, Aviv T**. **1993**. The histology of processes associated with the infection of resistant and susceptible wheat cultivars with Septoria tritici. *Plant Pathology* **42**: 737–743.

**Haas H, Eisendle M, Turgeon BG**. **2008**. Siderophores in Fungal Physiology and Virulence. *Annual Review of Phytopathology* **46**: 149–187.

**Hane JK, Rouxel T, Howlett BJ, Kema GH, Goodwin SB, Oliver RP**. **2011**. A novel mode of chromosomal evolution peculiar to filamentous Ascomycete fungi. *Genome Biology* **12**: R45.

**Haslbeck M, Vierling E**. **2015**. A first line of stress defense: Small heat shock proteins and their function in protein homeostasis. *Journal of Molecular Biology* **427**: 1537–1548.

**Heller J, Tudzynski P**. **2011**. Reactive Oxygen Species in Phytopathogenic Fungi: Signaling, Development, and Disease. *Annual Review of Phytopathology, Vol 49* **49**: 369–390.

**Jones JDG, Dangl JL**. **2006**. The plant immune system. Zig-zag-model. *Nature* **444**: 323–9.

**de Jonge R, Peter van Esse H, Kombrink A, Shinya T, Desaki Y, Bours R, van der Krol S, Shibuya N, Joosten MH a J, Thomma BPHJ**. **2010**. Conserved Fungal LysM Effector Ecp6 Prevents Chitin-Triggered Immunity in Plants. *Science* **329**: 953–955.

**Kellner R, Bhattacharyya A, Poppe S, Hsu TY, Brem RB, Stukenbrock EH**. **2014**. Expression Profiling of the Wheat Pathogen Zymoseptoria tritici Reveals Genomic Patterns of Transcription and Host-Specific Regulatory Programs. *Genome biology and evolution* **6**: 1353–65.

**Kema GHJ, Yu D, Rijkenberg FHJ, Shaw MW, Baayen RP**. **1996**. Histology of pathogenesis of Mycosphaerella graminicola in wheat. *Phytopathology* **7**: 777–786.

**Kim D, Pertea G, Trapnell C, Pimentel H, Kelley R, Salzberg SL**. **2013**. TopHat2: accurate alignment of transcriptomes in the presence of insertions, deletions and gene fusions. *Genome biology* **14**: R36.

**Kubicek CP, Starr TL, Glass NL**. **2014**. Plant Cell Wall-Degrading Enzymes and Their Secretion in Plant-Pathogenic Fungi. *Annual review of phytopathology*: 1–25.

**Laugé R**. **1997**. The in planta-produced extracellular proteins ECP1 and ECP2 of Cladosporium fulvum are virulence factors. *Molecular plant-microbe interactions : MPMI* **10**: 725–734.

**Lee W, Rudd JJ, Hammond-kosack KE, Kanyuka KK**. **2013**. Mycosphaerella graminicola LysM effector-mediated stealth pathogenesis subverts recognition through both CERK1 and CEBiP homologues in wheat. *Molecular plant-microbe interactions : MPMI* **27**: 236–243.

**Lendenmann MH, Croll D, Palma-Guerrero J, Stewart EL, McDonald BA**. **2016**. QTL mapping of temperature sensitivity reveals candidate genes for thermal adaptation and growth morphology in the plant pathogenic fungus Zymoseptoria tritici. *Heredity* **116**: 384–394.

**Li H, Handsaker B, Wysoker A, Fennell T, Ruan J, Homer N, Marth G, Abecasis G, Durbin R**. **2009**. The Sequence Alignment/Map format and SAMtools. *Bioinformatics* **25**: 2078–2079.

**Liu H, Zhang B, Li C, Bao X**. **2010**. Knock down of chitosanase expression in phytopathogenic fungus Fusarium solani and its effect on pathogenicity. *Current Genetics* **56**: 275–281.

**Love MI, Huber W, Anders S**. **2014**. Moderated estimation of fold change and dispersion for RNA-seq data with DESeq2. *Genome Biology* **15**: 550.

**Marshall R, Kombrink A, Motteram J, Loza-Reyes E, Lucas J, Hammond-Kosack KE, Thomma BPHJ, Rudd JJ**. **2011**. Analysis of two in planta expressed LysM effector homologs from the fungus Mycosphaerella graminicola reveals novel functional properties and varying contributions to virulence on wheat. *Plant physiology* **156**: 756–69.

**Mehrabi R, Taga M, Kema GHJ**. **2007**. Electrophoretic and cytological karyotyping of the foliar wheat pathogen Mycosphaerella graminicola reveals many chromosomes with a large size range. *Mycologia* **99**: 868–76.

**Mehrabi R, Zwiers L-H, de Waard M a, Kema GHJ**. **2006**. MgHog1 regulates dimorphism and pathogenicity in the fungal wheat pathogen Mycosphaerella graminicola. *Molecular plant-microbe interactions : MPMI* **19**: 1262–9.

**Ohtaki S, Maeda H, Takahashi T, Hasegawa F, Gomi K, Abe K, Yamagata Y, Nakajima T**. **2006**. Novel Hydrophobic Surface Binding Protein , HsbA , Produced by Aspergillus oryzae Novel Hydrophobic Surface Binding Protein , HsbA , Produced by Aspergillus oryzae. *Applied and environmental microbiology* **72**: 2407–2413.

**Pauw E De, Ghaffari A, Ghasemi V**. **2014**. *Agroclimatic zones map of Iran*.

**Perez-Nadales E, Almeida Nogueira MF, Baldin C, Castanheira S, El Ghalid M, Grund E, Lengeler K, Marchegiani E, Mehrotra PV, Moretti M, *et al.*** **2014**. Fungal model systems and the elucidation of pathogenicity determinants. *Fungal Genetics and Biology* **70**: 42–67.

**Plissonneau C, Stürchler A, Croll D**. **2016**. The evolution of orphan regions in genomes of a fungal pathogen of wheat. *mBio* **7**.

**Poppe S, Dorsheimer L, Happel P, Stukenbrock EH**. **2015**. Rapidly Evolving Genes Are Key Players in Host Specialization and Virulence of the Fungal Wheat Pathogen Zymoseptoria tritici (Mycosphaerella graminicola). *PLOS Pathogens* **11**: e1005055.

**Lo Presti L, Lanver D, Schweizer G, Tanaka S, Liang L, Tollot M, Zuccaro A, Reissmann S, Kahmann R**. **2015**. Fungal Effectors and Plant Susceptibility. *Annual review of plant biology* **66**: 513–545.

**Quaedvlieg W, Kema GHJ, Groenewald JZ, Verkley GJM, Seifbarghi S, Razavi M, Mirzadi Gohari a, Mehrabi R, Crous PW**. **2011**. Zymoseptoria gen. nov.: a new genus to accommodate Septoria-like species occurring on graminicolous hosts. *Persoonia* **26**: 57–69.

**Quinlan AR, Hall IM**. **2010**. BEDTools: A flexible suite of utilities for comparing genomic features. *Bioinformatics* **26**: 841–842.

**Ram A, Klis F**. **2006**. Identification of fungal cell wall mutants using susceptibility assays based on Calcofluor white and Congo red. *Nature protocols* **1**: 2253–2256.

**Robin JB, Arffa RC, Avni I, Rao NA**. **1986**. Rapid visualization of three common fungi using fluorescein-conjugated lectins. *Investigative Ophthalmology and Visual Science* **27**: 500–506.

**Roncero C, Duran A**. **1985**. Effect of Calcofluor White and Congo Red on Fungal Cell Wall Morphogenesis : In Vivo Activation of Chitin Polymerization. **163**: 1180–1185.

**Rounds CM, Lubeck E, Hepler PK, Winship LJ**. **2011**. Propidium Iodide Competes with Ca 2 + to Label Pectin in Pollen Tubes and Arabidopsis Root Hairs 1 [ W ][ OA ]. **157**: 175–187.

**Rudd JJ, Kanyuka K, Hassani-Pak K, Derbyshire M, Andongabo A, Devonshire J, Lysenko A, Saqi M, Desai NM, Powers SJ, *et al.*** **2015**. Transcriptome and Metabolite Profiling of the Infection Cycle of Zymoseptoria tritici on Wheat Reveals a Biphasic Interaction with Plant Immunity Involving Differential Pathogen Chromosomal Contributions and a Variation on the Hemibiotrophic Lifestyle Def. *Plant Physiology* **167**: 1158–1185.

**Sánchez-Vallet A, Mesters JR, Thomma BPHJ**. **2015**. The battle for chitin recognition in plant-microbe interactions. *FEMS Microbiology Reviews* **39**: 171–183.

**Sánchez-Vallet A, Saleem-Batcha R, Kombrink A, Hansen G, Valkenburg DJ, Thomma BPHJ, Mesters JR**. **2013**. Fungal effector Ecp6 outcompetes host immune receptor for chitin binding through intrachain LysM dimerization. *eLife* **2013**: 1–16.

**Schneider C a, Rasband WS, Eliceiri KW**. **2012**. NIH Image to ImageJ: 25 years of image analysis. *Nature Methods* **9**: 671–675.

**Shetty NP, Kristensen BK, Newmana MA, Møller K, Gregersen PL, Jørgensen HJL**. **2003**. Association of hydrogen peroxide with restriction of Septoria tritici in resistant wheat. *Physiological and Molecular Plant Pathology* **62**: 333–346.

**Shetty NP, Mehrabi R, Lütken H, Haldrup A, Kema GHJ, Collinge DB, Jørgensen HJL**. **2007**. Role of hydrogen peroxide during the interaction between the hemibiotrophic fungal pathogen Septoria tritici and wheat. *New Phytologist* **174**: 637–647.

**Soderlund C, Bomhoff M, Nelson WM**. **2011**. SyMAP v3.4: A turnkey synteny system with application to plant genomes. *Nucleic Acids Research* **39**.

**Stewart EL, Hagerty CH, Mikaberidze A, Mundt CC, Zhong Z, McDonald BA**. **2016**. An Improved Method for Measuring Quantitative Resistance to the Wheat Pathogen Zymoseptoria tritici Using High-Throughput Automated Image Analysis. *Phytopathology* **106**: 782–788.

**Stukenbrock EH, Jørgensen FG, Zala M, Hansen TT, McDonald BA, Schierup MH**. **2010**. Whole-genome and chromosome evolution associated with host adaptation and speciation of the wheat pathogen Mycosphaerella graminicola. *PLoS genetics* **6**: e1001189.

**Takahara H, Hacquard S, Kombrink A, Hughes HB, Halder V, Robin GP, Hiruma K, Neumann U, Shinya T, Kombrink E, *et al.*** **2016**. Colletotrichum higginsianum extracellular LysM proteins play dual roles in appressorial function and suppression of chitin-triggered plant immunity. *New Phytologist* **211**: 1323–1337.

**Thordal-Christensen H, Zhang Z, Wei Y, Collinge DB**. **1997**. Subcellular localization of H2O2 in plants. H2O2 accumulation in papillae and hypersensitive response during the barley-powdery mildew interaction. *Plant Journal* **11**: 1187–1194.

**Trapnell C, Hendrickson DG, Sauvageau M, Goff L, Rinn JL, Pachter L**. **2013**. Differential analysis of gene regulation at transcript resolution with RNA-seq. *Nature biotechnology* **31**: 46–53.

**Trapnell C, Roberts A, Goff L, Pertea G, Kim D, Kelley DR, Pimentel H, Salzberg SL, Rinn JL, Pachter L**. **2012**. Differential gene and transcript expression analysis of RNA-seq experiments with TopHat and Cufflinks. *Nature protocols* **7**: 562–78.

**Tucker SL, Talbot NJ**. **2001**. Surface attachment and pre-penetration stage development by plant pathogenic fungi. *Annual Review of Phytopathology* **39**: 385–417.

**Turrà D, El Ghalid M, Rossi F, Di Pietro A**. **2015**. Fungal pathogen uses sex pheromone receptor for chemotropic sensing of host plant signals. *Nature* **527**: 521–524.

**Yun CW, Tiedeman JS, Moore RE, Philpott CC**. **2000**. Siderophore-iron uptake in Saccharomyces cerevisiae: Identification of ferrichrome and fusarinine transporters. *Journal of Biological Chemistry* **275**: 16354–16359.

**Zhan J, McDonald BA**. **2011**. Thermal adaptation in the fungal pathogen Mycosphaerella graminicola. *Molecular Ecology* **20**: 1689–1701.
