# Supplementary material for: Highly flexible infection programs in a specialized wheat pathogen
Source: Ecol Evol. 2018 Dec 26;9(1):275–94. doi: 10.1002/ece3.4724 (PMC6342133; doi:10.1002/ece3.4724)
Supplement: Supplementary file 28 [file ECE3-9-275-s028.docx]

**Supporting Information**

**Text S1. Supplementary Information.**

Supplementary results, material and methods as well as tools and commands used for quantification of leaf area affected by ROS, for genome analyses, and for processing and analyses of *Z. tritici* transcriptome data.

**Table S1. Studied *Zymoseptoria tritici* isolates and overview of analyzed leaf material.**

**Table S2. Isolate-specific sampling schedules for transcriptome sequencing of infection stages.**

Post-inoculation time points were scheduled based on previous plant infection experiments to cover each of the four *Z. tritici* infection stages. Samples for transcriptome sequencing and analyses were collected at one to three different times points and eventually selected based on the results of microscopic analyses of central leaf sections. Selected samples are marked by *.

**Table S3. Detailed overview of stage-specific transcriptomes of *Z. tritici* isolates during wheat infection generated in this study.**

**Table S4. Gene annotation for the *Z. tritici* isolate Zt05.**

Gene annotation in .gff file format for the Zt05 genome assembly based on Illumina short reads (NCBI BioSample: SAMN04494882).

**Table S5. Gene annotation for the *Z. tritici* isolate Zt10.**

Gene annotation in .gff file format for the Zt10 genome assembly based on Illumina short reads (NCBI accession number: GCA_000223645.2).

**Table S6. Transposable element annotation for the *Z. tritici* isolate Zt05.**

Transposable element annotation in .gff file format for the Zt05 genome assembly based on Illumina short reads (NCBI BioSample: SAMN04494882).

**Table S7. Transposable element annotation for the *Z. tritici* isolate Zt10.**

Transposable element annotation in .gff file format for the Zt10 genome assembly based on Illumina short reads (NCBI accession number: GCA_000223645.2).

**Table S8. The three *Z. tritici* isolates vary in tolerance to abiotic stressors.**

**Table S9. SMRT Sequencing-based *de novo* genome assemblies, synteny analyses, and conserved genes.**

The table summarizes basic statistics of *de novo* genome assemblies generated for isolates Zt05 and Zt10 based on SMRT Sequencing long-read data, results of synteny analyses between IPO323/Zt09 chromosomes and Zt05 and Zt10 unitigs, and the presence/absence of IPO323/Zt09 genes and effector candidates in the genomes of Zt05 and Zt10.

**Table S10. *Zymoseptoria tritici* core genes and effector candidates.**

List of 10,426 genes and 370 effector candidate genes that are present in the genomes of the *Z. tritici* isolates Zt05, Zt09, and Zt10 based on nBLAST analyses. Genes are based on the *Z. tritici* genome annotation (Grandaubert *et al.*, 2015) and effector gene candidates were predicted by (Stukenbrock & Dutheil, 2018).

**Table S11. Comparison of expression on core and accessory chromosomes of *Z. tritici* during wheat infection.**

RPKM expression values calculated with Cuffdiff2 (Trapnell *et al.*, 2013) for genes and 1-kb windows located on the core (CC) and accessory (AC) chromosomes.

**Table S12. Genes that are significantly differentially expressed between infection stage A and B across all *Z. tritici* isolates.**

**Table S13. Genes that are significantly differentially expressed between infection stage B and C across all *Z. tritici* isolates.**

**Table S14. Genes that are significantly differentially expressed between infection stage C and D across all *Z. tritici* isolates.**

**Table S15. *Zymoseptoria tritici* biotrophic core effector candidates.**

**Table S16. *Zymoseptoria tritici* necrotrophic core effector candidates.**

**Table S17. Core *Z. tritici* genes that are differentially expressed between the three isolates during wheat infection.**

**Table S18. Core *Z. tritici* genes that are differentially expressed between the three isolates during wheat infection, sorted by up-regulation per isolate.**

**Table S19. *Zymoseptoria tritici* effector candidate genes that are differentially expressed between the three isolates during wheat infection.**

**Table S20. Summary for the analyses of gene distance to closest transposable element.**

**Figure S1. Examples for the six symptom level categories for necrosis and pycnidia coverage on *T. aestivum* cultivar Obelisk leaves infected with *Z. tritici*.**

**Figure S2. Generation of isolate- and stage-specific transcriptomes was enabled by confocal microscopy analyses.**

The schematic drawing illustrates how we selected samples for RNA-seq. Central sections of *Z. tritici*-infected wheat leaves from three independent plants (second leaf of each plant) were stained and analyzed by confocal laser-scanning microscopy while the remaining infected leaf material was pooled and ground in liquid nitrogen for total RNA extraction. RNA samples subjected to sequencing were chosen based on the morphological infection stage that we observed in the central leaf section by microscopy.

**Figure S3. Differences in colony morphology and abiotic stress tolerance between *Z. tritici* isolates.**

Growth of the isolates Zt05, Zt09, and Zt10 was tested under multiple stress conditions in comparison to the standard cultivation condition *in vitro* (solid YMS medium at 18°C, no light): growing conditions of wheat (20/22°C at 16-h day/8-h night rhythm), heat stress (28°C), oxidative stress (2 and 3 mM H_2_O_2_), osmotic stress (1 M sorbitol, 1 M NaCl), and cell wall stress (500 µg/mL Congo red, 200 µg/mL calcofluor white).

**Figure S4. Karyotype variation of *Z. tritici* field isolates.**

Pulsed-field gel electrophoresis shows number and size variations for small chromosomes (~225 to 1,460 kb) of *Z. tritici* isolates Zt05, Zt10, and Zt09. Standard chromosome size marker (M): *Saccharomyces cerevisiae*.

**Figure S5. Synteny plots. (A)** Synteny between Zt05 *de novo* assembled unitigs and IPO323 chromosomes. **(B)** Synteny between Zt10 *de novo* assembled unitigs and IPO323 chromosomes.

**Figure S6. *In-planta* virulence phenotypes of the *Z. tritici* isolates Zt05, Zt09, and Zt10 on leaves of the susceptible wheat cultivar Obelisk.**

Results of the three independent wheat infection experiments **(1-3)**. Quantitative differences in necrosis and pycnidia coverage of inoculated leaf areas were manually assessed at 28 days post inoculation based on six symptom levels: 0 (without visible symptoms), 1 (1% to 20%), 2 (21% to 40%), 3 (41% to 60%), 4 (61% to 80%), and 5 (81% to 100%).

**Figure S7. Disease development on wheat leaves infected with *Z. tritici* isolates Zt05, Zt09, and Zt10.**

**(A)** Photographs of wheat leaves taken at different time points after inoculation with *Z. tritici* isolates Zt05, Zt09, and Zt10. **(B)** Infected wheat leaves contained similar numbers of pycnidia. Scale bars = 500 µm.

**Figure S8. Symptom development on wheat leaves used for the ROS staining assay.**

Photographs of *Triticum aestivum* cv. Obelisk leaves taken at 4, 11, 14, 18, and 21 days post inoculation with *Z. tritici* isolates Zt05, Zt09, and Zt10 and mock treatment. Leaves were subsequently subjected to ROS detection staining.

**Figure S9. RNA-seq data principal component analysis plot based on rlog-transformed read counts for *Z. tritici* core genes.**

PC1 separates datasets from infections stages A and B from stages C and D. Stage-specific datasets from all isolates cluster together.

**Figure S10. Transcriptome data distance matrix based on rlog-transformed read counts for *Z. tritici* core genes.**

Datasets from stages A and B representing biotrophic growth form one cluster as do datasets of stages C and D representing necrotrophic growth.

**Figure S11. MA plots comparing replicates for each RNA-seq dataset.**

Pairwise comparisons of replicates for each wheat infection stage of each *Z. tritici* isolate without normalization. x-axis: mean log_2_ (read count per gene+1), y-axis: log (fold-change). The greatest variation among replicates was between the Zt10 stage C datasets.

**Figure S12. 597 genes are differentially expressed between the infection stages in all three isolates, and 79 genes are differentially expressed between more than two stages.**

The Venn diagram illustrates how genes that are differentially expressed between *Z. tritici* infection stages are shared between stage comparisons. Differential expression analyses were performed with DESeq2. Differentially expressed genes have *P*_adj_ ≤ 0.01 and an absolute log_2_ fold change between infection stages of ≥2. Small arrows (↑) indicate the stage in which genes are significantly up-regulated.

**Figure S13 1-3. Expression profiles of core *Z. tritici* biotrophic effector candidates** **based on normalized read counts per gene.**

Read counts were normalized by applying the regularized log transformation (rlog) function of DESeq2 (Love *et al.*, 2014) across the four core infection stages (A to D) and the three *Z. tritici* isolates Zt05, Zt09, and Zt10 and represent a measure of relative gene expression between infection stages and between isolates.

**Figure S14 1-4. Expression profiles of** ***Z. tritici* core necrotrophic effector candidates based on normalized read counts per gene.**

Read counts were normalized by applying the regularized log transformation (rlog) function of DESeq2 (Love *et al.*, 2014) across the four core infection stages (A to D) and the three *Z. tritici* isolates Zt05, Zt09, and Zt10 and represent a measure of relative gene expression between infection stages and between isolates.

**Figure S15. Expression profiles of three *Z. tritici* genes located on accessory chromosome 19 in Zt09.**

The neighboring genes *Zt09_chr_19_00071*, *Zt09_chr_19_00072,* and *Zt09_chr_19_00073* are significantly higher expressed in Zt10 during all four infection stages. Read counts were normalized across the four core infection stages (A to D) and the three *Z. tritici* isolates (Zt05, Zt09, and Zt10) and represent a measure of relative gene expression between infection stages and between isolates.

**Figure S16. Distribution of transcriptionally act****ive loci on core chromosome 11.**

**(A)** Heatmaps of log_2_-transformed RPKM expression values for 1-kb windows along IPO323/Zt09 core chromosome 11 and unitigs 16 and 10 in Zt05 and Zt10 for the four wheat infection stages. **(B)** Synteny plot comparing IPO323/Zt09 chromosome 11 and unitigs 16 and 10 in Zt05 and Zt10.

**Figure S17. Distribution of transcriptionally active loci on core chromosome 7.**

**(A)** Heatmaps of log_2_-transformed RPKM expression values for 1-kb windows along IPO323/Zt09 core chromosome 7 and unitig 5 in Zt10 and unitigs 70 and 75 in Zt05 for the four wheat infection stages. **(B)** Synteny plots comparing IPO323/Zt09 chromosome 7 and unitigs 70 and 75 of Zt05 and **(C)** unitig 5 of Zt10.

**Figure S18. Distribution of transcriptionally active loci on accessory chromosome 19.**

**(A)** Heatmaps of log_2_-transformed RPKM expression values for 1-kb windows along IPO323/Zt09 accessory chromosome 19 and the syntenic unitigs 24 and 16 in Zt05 and Zt10 for the four wheat infection stages. **(B)** Synteny plot comparing IPO323/Zt09 chromosome 19 and unitigs 24 and 16 of Zt05 and Zt10, respectively.

**Animation S1. *Zymoseptoria tritici* initial wheat infection stage.**

Tomographic animation of confocal image z-stack showing infection hypha of *Z. tritici* isolate Zt09 entering wheat leaf tissue by open stoma at 4 dpi. The hypha grows closely attached to stomatal guard cell. Nuclei and wheat cells are displayed in *purple* and fungal structures in *green*. Reference transmitted images are in *grey*. Scale bar = 25 µm.

**Animation S2. *Zymoseptoria tritici* initial wheat infection stage.**

Tomographic animation of confocal image z-stack showing epiphyllous proliferation, infecting hyphae, and hyphal growth inside wheat sub-stomatal cavity and mesophyll of *Z. tritici* isolate Zt05 at 3 dpi. Nuclei and wheat cells are displayed in *purple* and fungal structures in *green*. Reference transmitted images are in *grey*. Scale bar = 25 µm.

**Animation S3. Biotrophic colonization of wheat mesophyll by *Z. tritici* Zt05.**

Tomographic animation of confocal image z-stack showing epiphyllous hyphae as well as the dense biotrophic intercellular hyphal network of *Z. tritici* isolate Zt05 inside wheat mesophyll at 7 dpi. Long, straight hyphae grow in the interspace of wheat epidermis and mesophyll cells. Hyphae grow in close contact to plant cells. Nuclei and wheat cells are displayed in *purple* and fungal structures in *green*. Scale bar = 50 µm.

**Animation S4. Biotrophic colonization of wheat mesophyll by *Z. tritici* Zt09.**

Tomographic animation of confocal image z-stack showing biotrophic intercellular hyphae of *Z. tritici* isolate Zt09 inside wheat leaf tissue at 11 dpi. Nuclei and wheat cells are displayed in *purple* and fungal structures in *green*. Reference transmitted images are in *grey*. Scale bar = 25 µm.

**Animation S5. *Zymoseptoria tritici* Zt05 pycnidium development.**

Tomographic animation of confocal image z-stack showing the development of primal structures of *Z. tritici* Zt05 pycnidium in the wheat sub-stomatal cavity during the early lifestyle transition stage at 11 dpi. Nuclei and wheat cells are displayed in *purple* and fungal structures in *green*. Reference transmitted images are in *grey*. Scale bar = 25 µm.

**Animation S6. *Zymoseptoria tritici* Zt09 pycnidium development.**

Tomographic animation of confocal image z-stack showing the development of primal structures of *Z. tritici* Zt09 pycnidium in the wheat sub-stomatal cavity during the lifestyle transition stage at 13 dpi. Nuclei and wheat cells are displayed in *purple* and fungal structures in *green*. Reference transmitted images are in *grey*. Scale bar = 25 µm.

**Animation S7. Development of two pycnidium initials of *Z. tritici* Zt10.**

Tomographic animation of confocal image z-stack showing *Z. tritici* Zt10 pycnidium in the wheat sub-stomatal cavity developing from two initial stromata during the early lifestyle transition stage at 13 dpi. Nuclei and wheat cells are displayed in *purple* and fungal structures in *green*. The fluorescence of *Z. tritici* hyphae inside plant tissue is very weak. *Purple* fungal nuclei are mainly visible. Reference transmitted images are in *grey*. Scale bar = 25 µm.

**Animation S8. Mature pycnidia of *Z. tritici* Zt05.**

Tomographic animation of confocal image z-stack showing asexual pycnidia of *Z. tritici* isolate Zt05 with pycnidiospores during necrotrophic infection stage at 21 dpi. The intercellular space of wheat mesophyll is densely colonized by *Z. tritici* hyphae. Nuclei and wheat cells are displayed in *purple* and fungal structures in *green*. The fluorescence of *Z. tritici* hyphae inside the plant tissue is weak. *Purple* fungal nuclei are mainly visible. Reference transmitted images are in *grey*. Scale bar = 50 µm.

**Animation S9. Pycnidium of *Z. tritici* Zt09.**

Tomographic animation of confocal image z-stack showing asexual pycnidium of *Z. tritici* isolate Zt09 at 20 dpi. Hyphae grow in close contact to collapsing wheat mesophyll cells. Nuclei and wheat cells are in *purple* and fungal structures in *green*. Reference transmitted images are in *grey*. Scale bar = 25 µm.
